# A Multipurpose Metallophore and Its Copper Complexes with Diverse Catalytic Antioxidant Properties to Deal with Metal and Oxidative Stress Disorders: A Combined Experimental, Theoretical, and *In Vitro* Study

**DOI:** 10.1021/acs.inorgchem.4c00232

**Published:** 2024-07-30

**Authors:** Lucas B. Menezes, Raquel M. S. N. Sampaio, Lino Meurer, Bruno Szpoganicz, Rodrigo Cervo, Roberta Cargnelutti, Lukun Wang, Jiawen Yang, Rajeev Prabhakar, Christiane Fernandes, Adolfo Horn

**Affiliations:** † Departamento de Química, Universidade Federal de Santa Catarina, 88040-900 Florianópolis, SC, Brazil; ‡ Laboratório de Ciências Químicas, Universidade Estadual do Norte Fluminense Darcy Ribeiro, 28013-602 Campos dos Goytacazes, RJ, Brazil; § Departamento de Química, Universidade Federal de Santa Maria, 97105-900 Santa Maria, RS, Brazil; ∥ Department of Chemistry, University of Miami, Coral Gables, Florida 33146, United States

## Abstract

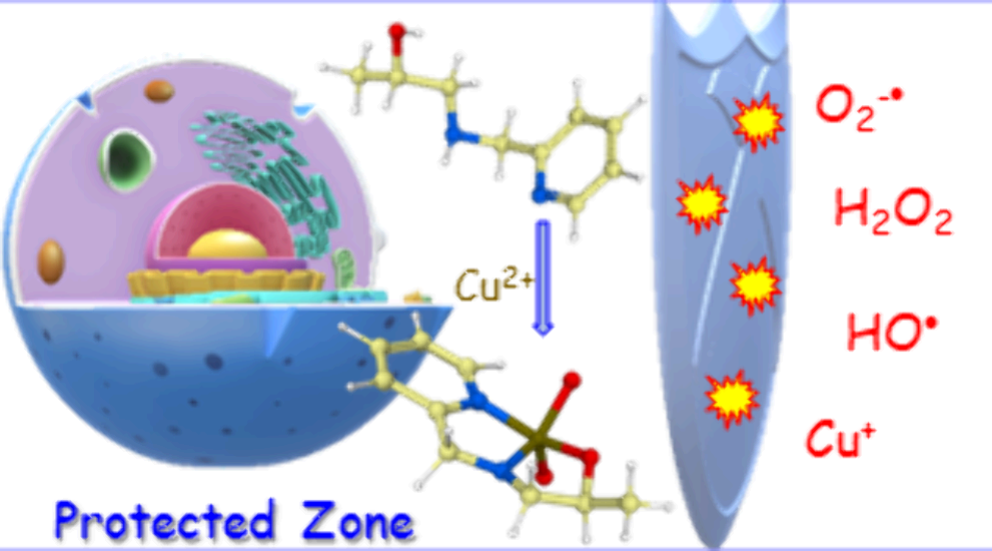

We report the discovery that the molecule 1-(pyridin-2-ylmethylamino)propan-2-ol (HL) can reduce oxidative stress in neuronal C6 glioma cells exposed to reactive oxygen species (O_2_^–•^, H_2_O_2_, and ^•^OH) and metal (Cu^+^) stress conditions. Furthermore, its association with Cu^2+^ generates [Cu(HL)Cl_2_] (**1**) and [Cu(HL)_2_](ClO_4_)_2_ (**2**) complexes that also exhibit antioxidant properties. Potentiometric titration data show that HL can coordinate to Cu^2+^ in 1:1 and 1:2 Cu^2+^:ligand ratios, which was confirmed by monocrystal X-ray studies. The subsequent ultraviolet–visible, electrospray ionization mass spectrometry, and electron paramagnetic resonance experiments show that they can decompose a variety of reactive oxygen species (ROS). Kinetic studies revealed that **1** and **2** mimic the superoxide dismutase and catalase activities. Complex **1** promotes the fastest decomposition of H_2_O_2_ (*k*_obs_ = 2.32 × 10^7^ M^–1^ s^–1^), efficiently dismutases the superoxide anion (*k*_cat_ = 3.08 × 10^7^ M^–1^ s^–1^), and scavenges the hydroxyl radical (RSA_50_ = 25.7 × 10^–6^ M). Density functional theory calculations support the formation of dinuclear Cu-peroxide and mononuclear Cu-superoxide species in the reactions of [Cu(HL)Cl_2_] with H_2_O_2_ and O_2_^•–^, respectively. Furthermore, both **1** and **2** also reduce the oxidative stress of neuronal glioma C6 cells exposed to different ROS, including O_2_^•–^ and ^•^OH.

## Abstract 2

The ligand 1-(pyridin-2-ylmethylamino)propan-2-ol (HL) protected cells stressed with hydrogen peroxides, superoxide anion, hydroxyl radicals, and Cu^+^. HL forms mononuclear compounds in complexes with Cu^2+^, which also show antioxidant activities, mimicking superoxide dismutase and catalase metalloenzymes. The results suggest that HL acts as a metallophore and serves as a promising candidate for therapeutic intervention for various neurodegenerative diseases associated with reactive oxygen species and metal stress disorders.

## Introduction

In nature, molecular oxygen is converted into reactive oxygen species (ROS) such as the superoxide anion (O_2_^•–^) and singlet oxygen (^1^O_2_) in a wide range of biological processes and metabolic functions.^1−3^ In particular, they are required for the destruction of pathogens by white blood cells, intercellular and intracellular signaling, stimulation of cell division, and promotion of DNA replication and cell proliferation.^4−6^ These species are constantly generated as part of the mitochondrial energy production pathway (MEPP).^7,8^ They are also endogenously produced by white blood cells through NADPH-oxidase activity.^9^ Additionally, hypoxia and hyperoxia have been reported to generate different ROS.^10^ Furthermore, their creation can also be induced by different exogenous sources like pollution, alcohol, tobacco smoke, transition metal ions, industrial solvents, pesticides, certain drugs like paracetamol, and ionizing radiation.^11^ The overproduction of ROS can alter the normal redox status of the cells leading to increased oxidative stress.^12−15^

As a primary ROS, the superoxide anion can generate multiple secondary metabolite species, including hydrogen peroxide (H_2_O_2_), peroxynitrite (ONOO^–^), and peroxyl (^•^OOH) and hydroxyl (^•^OH) radicals. Uncontrolled production of these species has been associated with several diseases, including neurodegenerative disorders,^16−18^ various types of cancers (colorectal, prostate, breast, lung, and bladder),^19−21^ cardiovascular,^22−24^ and respiratory conditions.^25,26^ For instance, peroxidation in neuronal membranes has been reported to occur in the brain of Alzheimer’s disease (AD) patients.^27,28^ Furthermore, metal dyshomeostasis of ions such as iron or copper has been described, mainly in regions of amyloid plaque pathology. Amyloid-β (Aβ) aggregates are one of the hallmarks of AD. They are prone to forming Fe/Cu(Aβ) complexes and generate ROS via Fenton and Haber–Weiss reactions,^29,30^ inducing an imbalance in the redox status by generating the extremely reactive ^•^OH radical.^31−36^ The occurrence of oxidative stress induced by the accumulation of metal ions (Fe and Cu) in the brain has been associated with the development of dementia.^37−39^

Aerobic organisms utilize metalloenzymes such as superoxide dismutases (SOD) and catalases (CAT) to combat oxidative stress.^10^ They play an indispensable role in providing antioxidant protection to biological systems against ROS.^10^ SOD is one of the cell’s most powerful antioxidants. It promotes the dismutation reaction in which one superoxide radical is oxidized to oxygen and the second one is reduced to hydrogen peroxide.^40^ On the contrary, CAT, which is abundant in the peroxisomes, breaks down the H_2_O_2_ into water and molecular O_2_.^41,42^ It is known that the levels of SODs decrease with age, resulting in the increase in the level of ROS.^10^ One of the common strategies for dealing with this situation is the consumption of nutraceutical ingredients such as vitamins (A, C, and E) and flavonoids, because they can act as potent antioxidants and metal chelators.^43−45^ Furthermore, they have long been recognized to possess anti-inflammatory, antiallergic, hepatoprotective, antithrombotic, antiviral, and anticarcinogenic activities, showing a direct relation between these diseases and ROS.^46,47^ A more recent approach is the development of synthetic mimics of antioxidant metalloenzymes such as SOD and CAT.^48−50^ Their examples include coordination compounds synthesized with porphyrins, H_2_salen-type ligands, macrocycles, corroles, and polydentate amines.^51−61^ Recently, Policar and co-workers reviewed strategies for the design of efficient SOD mimics and their basic requirements like cellular uptake, speciation, catalytic activities, and distribution in cells.^62^

**Figure 1:**
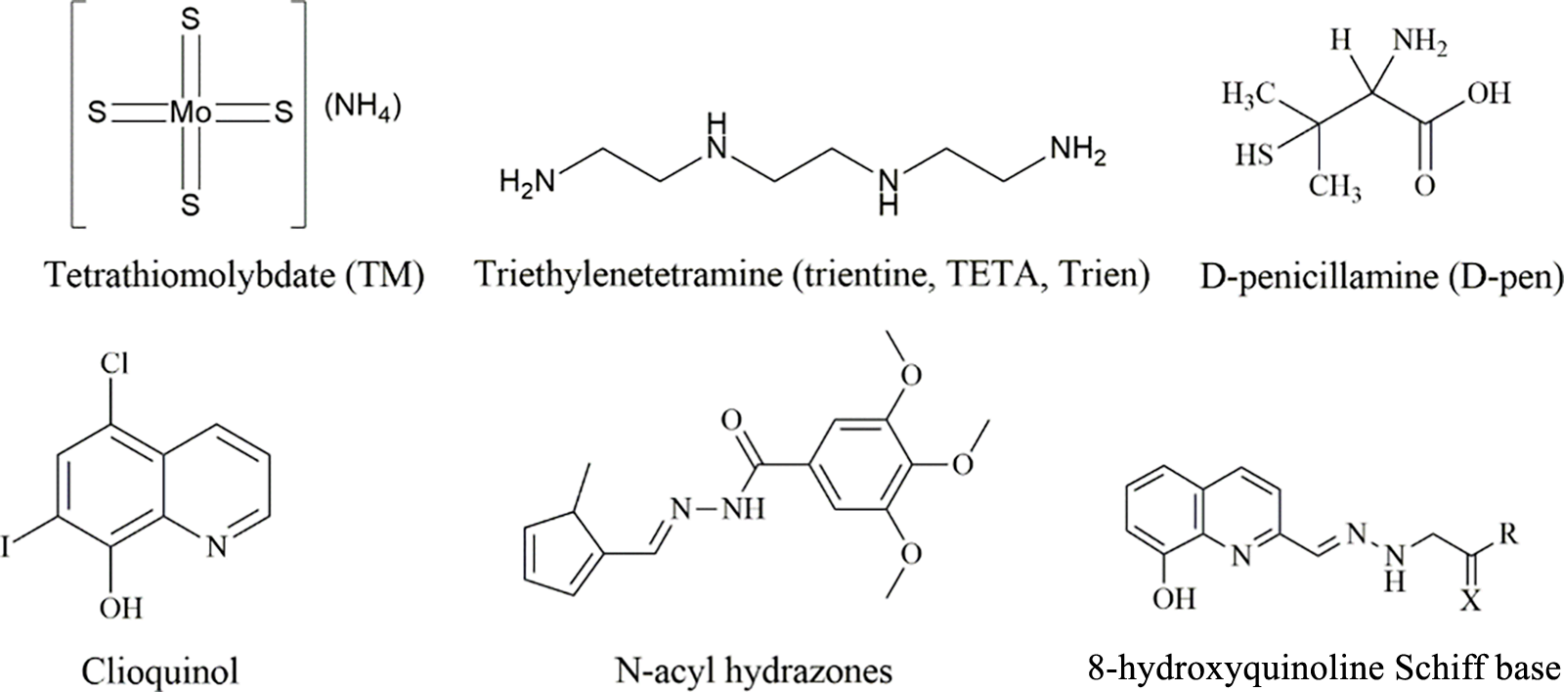
Examples of molecules studied as copper chelators with the aim of treating neurodegenerative diseases.

Oxidative stress is also associated with dyshomeostasis of transition metal ions.^63−65^ Although they are relevant to the functioning of cellular machinery,^66,67^ the disruption of metal homeostasis leads to their abnormal accumulation, resulting in a metal stress situation.^68−70^ For example, an increased concentration of iron and copper inside cells induces an imbalance in the cell’s redox status by reacting with hydrogen peroxide and forming the harmful ^•^OH radical,^31−33^ which subsequently can damage critical macromolecules inside cells.^71−73^ Therefore, there is a great deal of interest in designing small chelating molecules (metallophores) that can scavenge metal ions and mitigate the perilous effects of oxidative stress and inflammation.^74−80^ For instance, chelating ligands such as tetrathiomolybdate (TM), triethylenetetramine (trientine, TETA, or Trien), d-penicillamine (D-pen), and clioquinol (Figure 1) have been utilized to mitigate the toxicity associated with an excess of copper.^81,82^ The latter has been investigated in a phase II clinical trial in AD.^81,82^ Another example of this approach is the synthesis of *N*-acyl hydrazones and their potential use as a metallophore via sequestering Cu(II) ions and decreasing the level of production of ROS by the Cu(Aβ) system.^83^ Beraldo and colleagues have also shown that 8-hydroxyquinoline Schiff base ligands coordinate to Cu(II) and inhibit Aβ agregation and that the ligands exhibit antioxidant properties on the ABTS^•+^ radical cation.^84^ It has also been proposed that metallophores should present a medium formation constant for application in copper chelation therapy.^75^

**Scheme 1:**
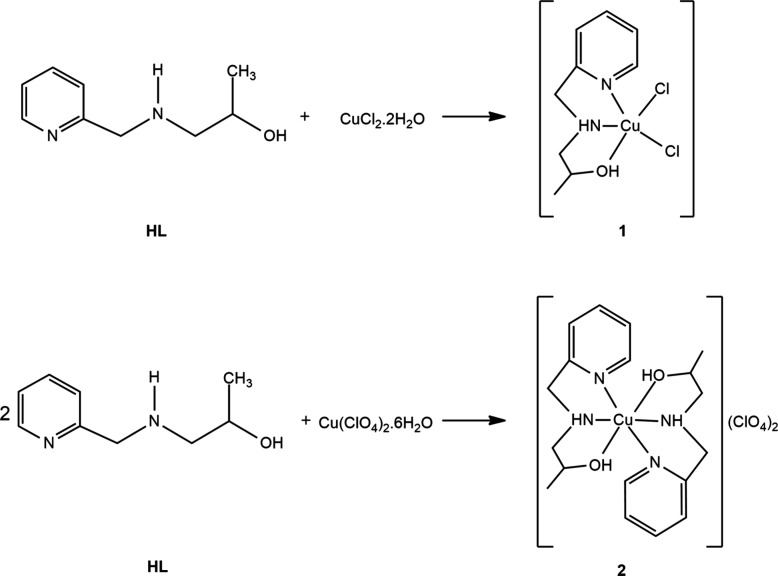
Synthetic Routes Employed to Obtain Complexes **1** and **2**

To develop therapeutic alternatives for the treatment of metal and oxidative stress disorders, we investigated the ability of a tridentate ligand [HL (Scheme 1)] to chelate copper ions using a plethora of experimental and theoretical techniques and cell-based assays. Moreover, the Cu(II)-bound complexes {[Cu(HL)Cl_2_] (**1**) and [Cu(HL)_2_](ClO_4_)_2_ (**2**)} of HL are also found to exhibit intrinsic catalytic antioxidant properties against the superoxide anion, hydrogen peroxide, and hydroxyl radical. In addition to the synthesis and characterization of these complexes, their mechanisms of action of superoxide dismutase and catalase activity are studied through kinetic experiments and density functional theory (DFT) calculations.

## Experimental Section

### Materials and Methods

The HL ligand and its Cu(II) complexes were synthesized using reagents purchased from Sigma-Aldrich. The ultraviolet–visible (UV–vis), electrochemical, and ESI-(+)-MS investigations were carried out by using spectroscopic, high-performance liquid chromatography, and mass spectroscopy (MS) quality solvents. ^1^H and ^13^C nuclear magnetic resonance (NMR) spectra were recorded on an Ascend 500 Advance III HD Bruker instrument operating at 500 MHz for ^1^H and 125 MHz for ^13^C. Chemical shifts (δ) are given in parts per million (with *J* values in hertz), and the spectra were recorded in appropriate deuterated solvents, as indicated. Tetramethylsilane (0 ppm) was employed as the standard. Elemental analyses (CHN) were performed with a PerkinElmer 2400 CHN analyzer, and infrared spectra were recorded with a Shimadzu FT-IR 8300 spectrophotometer. The solid samples were prepared in a KBr pellet, and spectra were recorded over the range of 400–4000 cm^–1^. UV–vis spectra of the ligand and the copper complexes were recorded with a Varian Cary 50 Bio spectrophotometer. Full scan mass spectra (MS mode) were recorded with a MicroTOF LC Bruker Daltonics spectrometer equipped with an electrospray source operating in positive ion mode. Samples were dissolved in a 50:50 MeOH/H_2_O solution and injected into the apparatus by direct infusion. The melting points were determined with a Microquimica MQAPF-301 device. The electrochemical properties of the complexes were evaluated using cyclic voltammetry (CV), conductometry, and potentiometric titration. CV studies were performed with an Autolab PGSTAT 10 potentiostat/galvanostat in a water solution containing 0.1 mol L^–1^ LiClO_4_ as the supporting electrolyte under an argon atmosphere at 25 °C. The electrochemical cell employed was a standard three-electrode configuration: a platinum working electrode, a platinum-wire auxiliary electrode, and a platinum-wire pseudoreference electrode. The potassium ferro-ferricyanide redox couple was used as an external reference (0.426 V vs NHE).^85^ The electrical conductivity of a solution of each complex (1 × 10^–3^ mol L^–1^) was measured with an MS Tecnopon mCA-150 apparatus.

The potentiometric studies were carried out with a Corning 350 digital pH meter fitted with blue-glass and Ag/AgCl reference electrodes calibrated to read −log[H^+^] directly, designated as pH. Doubly distilled water in the presence of KMnO_4_ was used to prepare the solutions. For the ligands, the study was performed in a 70:30 ethanol/water solution, while the copper complexes were studied in water. The electrode was calibrated using the data obtained from the potentiometric titration of a known volume of a standard 0.100 mol L^–1^ HCl solution with a standard 0.100 mol L^–1^ KOH solution. The ionic strength of the HCl solution was maintained at 0.100 mol L^–1^ upon the addition of KCl. The measurements were carried out in a thermostated cell (25 °C) containing the compounds or ligand solution (0.5 mmol/50 mL). The experiments were performed by using an automatic titrator (Titrando 888, Metrohn) under an argon flow to eliminate the presence of CO_2_. The samples were initially acidified to pH 2 and then titrated by the addition of fixed volumes of a standard CO_2_-free KOH solution (0.100 mol L^–1^).

The titration curves were processed with BEST7,^86^ which has as input file: titrant concentration, an initial value for the number of millimoles of the species under investigation, the volume increments of the titrant added, and the corresponding values of pH measured during titration. The program uses the least-squares method to minimize the differences between the calculated pH values and the experimental ones obtained during the titration, keeping fixed the values of the known equilibrium constants, such as those for the hydrolysis of the metal ion,^87^ and varying the values of the constants to be determined, minimizing the error. The quantity of each species is also determined, and the species distribution diagrams were obtained with SPE and SPEPLOT.^86^

Electron paramagnetic resonance (EPR) spectra were recorded using a Bruker EMX micro-9.5/2.7/P/L system using a highly sensitive cylindrical cavity operating in the X-band (∼9.5 GHz) at 120 K. The following experimental settings were used: central magnetic field of 3000 G, sweep width of 1800 G, microwave power of 5 mW, modulation amplitude of 4 G, modulation frequency of 100 kHz, receiver gain of 30 dB, and 16 scans. The spectra were simulated using SpinFit software provided by the manufacturer (Bruker), and MgO:Cr(III) (*g* = 1.9797) was used as a marker. Full scan mass spectra (MS mode) were recorded on an electrospray ionization (ESI-MS) Amazon X Ion Trap MS instrument (Bruker Daltonics), in a positive mode, with a ion-source voltage of 4500 V, a flow injection or flow rate of 180 μL/h, nebulizing gas (0.4 bar) and dry gas (4 L min^–1^), ion-source temperatures of 180 and 200 °C, and a scan range of *m*/*z* 100–2000.

X-ray diffraction data of complexes **1** and **2** were measured with a single-crystal Bruker D8 Venture diffractometer equipped with a Photon 100 detector and Incoatec microfocus Montel optic X-ray tube with Mo Kα radiation (λ = 0.71073 Å). The measurements were performed at 100(2) and 300(2) K for complexes **1** and **2**, respectively. The frames were recorded with the φ and ω scan method by frame using APEX4 software. The structures were determined and refined with the SHELX program package.^88,89^ All non-hydrogen atoms were refined with anisotropic displacement parameters. The hydrogen atoms of the OH groups were successfully located from difference Fourier maps and refined by using a riding model. The molecular structure of complexes was drawn with Diamond (version 4.6.0).^90^ The crystallographic information files (CIF) for complexes **1** and **2** were deposited at the Cambridge Crystallographic Data Centre (CCDC) under identification numbers 2312762 and 2312763, respectively. These data can be obtained free of charge via http://www.ccdc.cam.ac.uk/conts/retrieving.html (or from the Cambridge Crystallographic Data Centre, 12, Union Road, Cambridge CB2 1EZ, UK; fax: + 44 1223 336033). Selected crystallographic data are presented in Table S1.

### Syntheses

#### Synthesis of the Ligand 1-[(Pyridin-2-ylmethyl)amino]propan-2-ol (HL)

The ligand HL was obtained via a condensation between 2-pyridine carboxaldehyde and 2-amino-1-propanol, following reduction with NaBH_4_, as described previously,^91,92^ resulting in a yellowish oil: yield 71%; ^1^H NMR (CD_3_Cl) δ 8.54 (d, *J* = 5.13, 1H), 7.63 (dd, *J*_1_ = 4.76, *J*_2_ = 7.69, 1H), 7.26 (d, *J* = 7.69, 1H), 7.15 (d, *J* = 7.69, 1H), 3.91 (s, 2H), 3.81 (m, 1H), 2.74 (dd, *J*_1_ = 12.08, *J*_2_ = 2.23, 1H), 2.41 (dd, *J*_1_ = 12.08, *J*_2_ = 9.52, 1H), 1.13 (d, *J* = 6.22, 3H).

#### Syntheses of Complexes [Cu(HL)Cl_2_] (**1**) and [Cu(HL)_2_](ClO_4_)_2_ (**2**)

Complex **1** was synthesized by reacting equimolar amounts of ligand HL (1 mmol, 166 mg) and CuCl_2_·2H_2_O (1 mmol, 170 mg) in methanol under reflux for 30 min (Scheme 1). After the solution was allowed to stand for a few days, blue crystals with a parallelepiped shape, suitable for crystallographic studies, were obtained: yield 52%; IR (KBr, cm^–1^) 2934 (νCH_3_), 1588, 1575, 1478, 1441 (νC=N, νC=C), 761 (δCH_pyridine_); UV–vis [λ_max_/nm(ε/L mol^–1^ cm^–1^)]: deionized water 595 (28), phosphate buffer 602 (54), DMSO 716 (71). Elemental analysis (CHN) found/calcd for C_9_H_14_Cl_2_N_2_OCu (MW = 300.66 g mol^–1^): C, 35.83%/35.83%; H, 4.62%/4.68%; N, 9.19%/9.29%.

Complex **2** was synthesized using a protocol similar to that utilized for **1**. The difference was that copper perchlorate was used instead of copper chloride and a 1:2 metal:ligand ratio was employed. After a few days, a blue powder was isolated, which was recrystallized in a 1:1 methanol/acetonitrile solvent, resulting in blue crystals with a rhombus shape: yield 37%; IR (KBr, cm^–1^) 2934 (νCH_3_), 1607, 1568, 1477, and 1441 (νC=N and νC=C), 772 (δCH_pyridine_); UV–vis [λ_max_/nm(ε/L mol^–1^ cm^–1^)] deionized water 602 (54), phosphate buffer 600 (19), DMSO 710 (19). Elemental analysis (CHN) found/calcd for C_18_H_28_Cl_2_N_4_O_10_Cu (MW = 594.88 g mol^–1^): C, 36.51%/36.34%; H, 4.69%/4.74%; N, 9.27%/9.41%.

### *In Vitro* Oxidative Stress Mitigation in Rat Glioma C6 Cells

#### Cell Culture

Rat C6 glioma cells were grown in a 25 cm^2^ culture flask containing Dulbecco’s modified Eagle medium (DMEM) supplemented with 10% (v/v) fetal bovine serum (FBS) at 37 °C in a 5% CO_2_ atmosphere.

#### Cell Viability Assays

The possible toxic effects of the compounds on the cells were evaluated on the basis of the reduction of 3-(4,5-dimethylthiazol-2-yl)-2,5-diphenyltetrazolium bromide (MTT). For these assays, 1 × 10^5^ cells per well were seeded in 96-well plates and cultured with DMEM supplemented with 10% (v/v) FBS at 37 °C in a 5% CO_2_ atmosphere. After 24 h, the cells were washed and directly subjected to treatment with a solution of the compounds (25 μmol L^–1^) prepared from dilution in DMEM supplemented with 10% (v/v) FBS. As a negative control, the cells were cultured in DMEM supplemented with 10% (v/v) FBS in the absence of the compound. As a positive control, the cells were cultured with 10 μmol L^–1^ rotenone, as previously described.^93^ After being treated for 24 h, the culture supernatant was removed, and 15 μL of MTT (5 mg/mL) in phosphate-buffered saline (PBS, pH 7.2) was added to each well for 1 h. The formazan crystals were subsequently solubilized by the addition of 100 μL of dimethyl sulfoxide (DMSO), and the data were recorded at 570 nm in a model M200 Tecan Infinite microplate reader.

#### Evaluation of ROS Mitigation on Rat Glioma C6 Cells Using DCFH-DA

To evaluate redox homeostasis, the level of ROS was assessed using the redox-sensitive dye 2′,7′-dichlorodihydrofluorescein diacetate (DCFH-DA). Rat C6 glioma cells were seeded at a density of 1 × 10^5^ cells/well in a 96-well plate and cultured in high-glucose DMEM supplemented with 10% (v/v) FBS at 37 °C in a 5% CO_2_ atmosphere. The cells were pretreated with the compounds (25 μmol L^–1^) and incubated for 24 h. The cells were washed and subjected to different oxidative stress models for 1 h, including (i) metallic stress via the addition of 25 μmol L^–1^ CuCl, (ii) direct addition of a 25 μmol L^–1^ hydrogen peroxide solution, (iii) superoxide anion (O_2_^•–^) generated by the xanthine (120 mmol L^–1^)/xanthine oxidase (0.1 unit/mL) enzymatic system, and (iv) hydroxyl radical (^•^OH) generated by the Fenton reaction using 0.4 μmol L^–1^ CuCl and 4 μmol L^–1^ H_2_O_2_. The concentrations refer to the final concentrations of each well. After exposure to the oxidative stress conditions, the cells were washed and incubated with DCFH-DA (10 μmol L^–1^) for 45 min. The fluorescence was measured using the model M200 Tecan Infinite microplate reader with excitation and emission wavelengths of 450 and 535 nm, respectively.

### *In Vitro* Antioxidant Activity

#### Kinetics and Spectroscopic Investigation of the Catalase (CAT) Activity

Kinetic studies to evaluate the CAT activity of the copper compounds were carried out by quantifying the oxygen produced using an oxygraph containing a Clark electrode (model Oxygraph+ from Hansatech Instruments Ltd.) during the reaction of solutions containing different concentrations of H_2_O_2_ and the copper complexes. The study was performed in triplicate, using a phosphate buffer solution (0.050 mol L^–1^, pH 7.8), at 25 °C under stirring. Hydrogen peroxide solutions of different concentrations were prepared using a stock solution whose concentration was previously determined by titration with sodium thiosulfate (0.100 mol L^–1^).^94^ Pseudo-first-order treatment was employed for the evaluation of the kinetic parameters of the reaction and to establish the rate law. To determine the reaction order concerning the complexes, the final concentration of H_2_O_2_ was kept constant (428 mmol L^–1^) while the concentration of the complexes was changed (71.4, 95.2, 119, 143, 167, and 190 μmol L^–1^), resulting in a final volume of 2.1 mL. On the contrary, to determine the reaction order for H_2_O_2_, the final concentrations of the complexes were fixed at 119 μmol L^–1^ and the final concentration of H_2_O_2_ was 14.3, 21.4, 28.6, 35.7, 42.8, or 50 mmol L^–1^.

The interaction between H_2_O_2_ and copper complexes was also investigated by EPR spectroscopy. Fresh solutions of the complexes (1 × 10^–3^ mol L^–1^) and H_2_O_2_ (1 × 10^–3^ mol L^–1^) were prepared in PBS. Aliquots of each complex and hydrogen peroxide (150 μL each) were added to an EPR tube. After reaction for 30 s, the solutions were frozen (120 K) and spectra were recorded. The tube was removed from the cavity, left at room temperature until a greenish-blue solution was obtained (∼5 min), and then frozen again to measure a new spectrum. This procedure (thawing and freezing) was carried out several times until no more changes in the spectrum were observed. This study was repeated in duplicate. The interaction between copper(II) complexes and hydrogen peroxide was also evaluated by UV–vis spectroscopy. Complexes and H_2_O_2_ solutions were prepared in PBS at concentrations of 1 and 100 mmol L^–1^, respectively. Aliquots of the complexes and H_2_O_2_, with equal volumes, were mixed in a cuvette, and the spectra were recorded after reaction for 1, 24, and 48 h at room temperature.

The interactions between the complexes and hydrogen peroxide were also investigated by ESI-(+)-MS. Spectra of the reactions were recorded in MeOH at a 1:10 complex:H_2_O_2_ ratio.

#### Kinetics and Spectroscopic Investigation of the Superoxide Dismutase (SOD) Activity

The SOD activity of the complexes was evaluated in triplicate employing the nitroblue tetrazolium (NBT) method, using the xanthine/xanthine oxidase system as a source of the superoxide anion, as described previously.^61^ Stock solutions of xanthine (4.5 × 10^–4^ mol L^–1^), NBT (5.6 × 10^–5^ mol L^–1^), xanthine oxidase (0.2 unit mL^–1^), and the complexes (1.5 × 10^–6^ mol L^–1^) were prepared in phosphate buffer (PBS, 0.05 mol L^–1^, pH 7.8). The control reaction was followed by the addition to a quartz cuvette (1 cm path length) of 0.4 mL of PBS, 0.4 mL of the xanthine solution, and 1.0 mL of NBT. The absorption reading started immediately after the addition of 0.2 mL of the solution containing xanthine oxidase, reaching a final volume of 2 mL. The SOD activity of the compounds was evaluated by adding 50, 100, 200, 300, or 400 μL of the stock solution of the complexes to the solution containing xanthine, PBS, and NBT, before the addition of xanthine oxidase (0.2 mL). The final volume was kept at 2.0 mL by decreasing the volume of PBS added, which is dependent on the volume of the complex employed in the experiment. The increase in the absorption at 560 nm was followed over time. The IC_50_ was determined as described by Novotná et al.^95^ by plotting *A*/*a* versus the concentration of the complex, where *A* is the maximum absorption reached in the absence of the complex and *a* is the maximum absorption obtained with a determined concentration of the compound under evaluation. The IC_50_ is the concentration at which *A*/*a* = 2. The obtained IC_50_ was transformed into *k*_cat_ by using the equation proposed by McCord and Fridovich: *k*_cat_ = *k*_NBT_ × [NBT]/IC_50_, where *k*_NBT_ = 5.94 × 10^4^ M^–1^ s^–1^.^96,97^

The SOD-like activity of the complexes was also studied by EPR. A solution containing the superoxide anion radical was generated using the procedure described by Valentine et al.^98^ Briefly, 7 mg of KO_2_ were stirred overnight in 1 cm^3^ of dry DMSO, resulting in a pale-yellow solution (∼0.1 mol L^–1^). The KO_2_ concentration was quantified by electronic spectroscopy (λ_max_ = 260 nm, and ε = 2686 L mol^–1^ cm^–1^).^98,99^

EPR spectra for each complex and for the superoxide radical were recorded at 120 K. Different concentrations of the KO_2_ solution were added to an EPR tube containing 150 μL of a solution (4 × 10^–3^ mol L^–1^) containing the complex under investigation. The experiment was performed using various complex:KO_2_ ratios. EPR spectra were recorded after reaction for 30 s at 120 K. This study was also repeated in duplicate.

The interaction between KO_2_ and complexes **1** and **2** was also evaluated by UV–vis spectroscopy. Complexes and KO_2_ solutions were prepared in dry DMSO at a concentration of 10 mmol of L^–1^. An aliquot of 500 μL of KO_2_ was added to a cuvette (800 μL) containing 100 μL of the Cu(II) complex. UV–vis spectra were recorded every 3 s until there were no more changes in the spectrum.

#### Kinetic and Spectroscopic Investigation of Antioxidant Activity against the Hydroxyl Radical

The generation of the hydroxyl radical was performed using an established protocol available in the literature.^33,100^ Stock solutions of the spin trap DMPO (400 mmol L^–1^), H_2_O_2_ (4 mmol L^–1^), [Fe(NH_4_)_2_(SO_4_)_2_]·6H_2_O (0.4 mmol L^–1^), and complexes **1** and **2** (0.4 mmol L^–1^) were prepared in Milli-Q water. Initially, 10 μL of each complex solution (in the concentration range of 100–1000 μmol L^–1^) was mixed with 10 μL of DMPO. Then, the ^•^OH radical was generated by the Fenton reaction through the concomitant addition of 10 μL of H_2_O_2_ and an aqueous solution of [Fe(NH_4_)_2_(SO_4_)_2_]·6H_2_O, resulting in a DMPO:H_2_O_2_:Fe ratio of 100:1:0.1.^33,100^ The reaction mixture was transferred to a glass tube capillary (inside diameter of 1.0 mm), and the spectra were recorded at 291 K after reaction for 3 min.

### Computational Procedure

The X-ray structures of **1** and **2** were used as starting models for DFT calculations. The geometry optimizations of all reactants, intermediates, transition states, and products were carried out using Gaussian 16^101^ utilizing the MPW1PW91 functional.^102^ The LANL2DZ basis set, including the Hay–Wadt effective core potential was used for Cu, while all other atoms were treated using the 6-31G(d,p) basis set.^103−105^ To improve the accuracy of the computed energies, single-point calculations were performed using a basis set of triple-ζ quality. For the Cu atoms, the LANL2TZ basis set was utilized, while the remaining atoms were treated using the 6-311G+(d,p) basis set.^103,106^ The solvation effects were computed using the integral equation formalism variant polarizable continuum model (IEFPCM).^107,108^ In this approach, the ligand and substrate environments were simulated using DMSO with a dielectric constant of 46.7. The transition states were verified to exhibit a single imaginary frequency specifically associated with the reaction coordinates. To account for thermal and zero-point vibrational effects, corrections were applied to the final energies at a temperature of 298 K and a pressure of 1 atm. In these calculations, dispersion effects were incorporated utilizing the D3 version of Grimme’s empirical dispersion with the Becke–Johnson damping (GD3BJ) package.^109,110^

## Results and Discussion

### Synthesis and Structures of Complexes **1** and **2**

The coordination of Cu(II) with the ligand HL was previously reported by Reedijk and co-workers.^92^ They observed the formation of a mononuclear copper(II) complex. We have observed that depending on the ligand:Cu(II) ratio, different mononuclear copper(II) complexes are formed. In the reaction between HL and CuCl_2_·2H_2_O employing a 1:1 ligand:metal ratio, a mononuclear pentacoordinated Cu(II) compound (**1**) is isolated in which one molecule of HL and two labile ligands (chloro) are bound to the Cu(II) center. In comparison, using a 2:1 ligand:metal ratio, a mononuclear hexacoordinated Cu(II) complex (**2**) that possesses two molecules of the ligand HL is generated. Both complexes are highly soluble in acetonitrile, DMSO, water, and even phosphate buffer (pH 7.0–8.0), a very useful feature in terms of biological activity. UV–vis spectrometry stability studies indicate that both compounds show the same spectra at a concentration of 10^–3^ mol L^–1^ in a PBS solution at physiological pH and remain stable when monitored for 48 h at 37 °C (Figure S1). The elemental analyses support the chemical composition provided by the X-ray diffraction, and the conductivity measurement reveals that **1** and **2** are neutral and dicationic species in MeOH, respectively.

### Molecular Structure of Complexes 1 and 2

**Figure 2:**
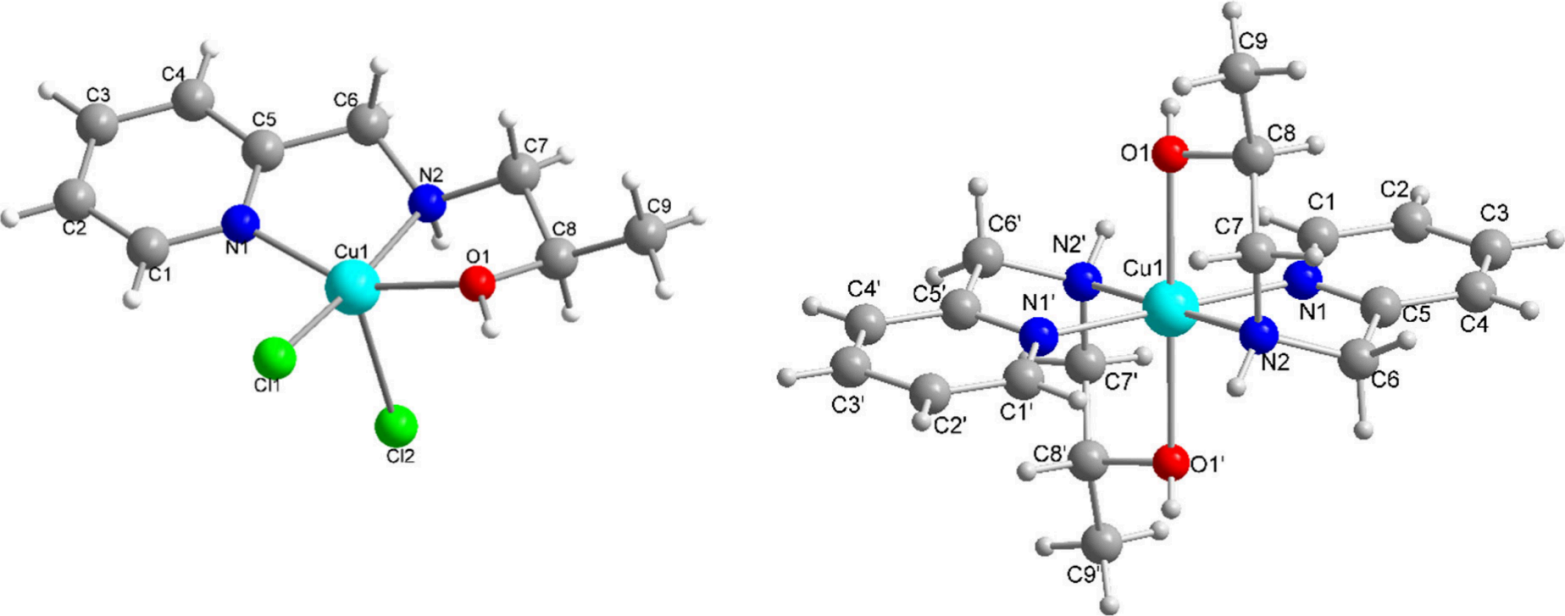
X-ray structures of complexes **1** (left) and **2** (right) and the corresponding atom labeling scheme. Perchlorate anions have been omitted from compound **2**. Symmetry operation is used to generate equivalent atoms: prime = −*x*, 1 – *y*, 1 – *z*.

Monocrystal X-ray investigations reveal that both **1** and **2** are mononuclear Cu(II) complexes (Figure 2). The coordination environment of the copper center in compound **1** is (N, N, O, Cl, and Cl) and in **2** is (N, N, N, N, O, and O). This difference is due to the presence of only one tridentate ligand in **1** and two ligands in **2**. However, the alcohol group remains protonated in both compounds. Compound **1** shows a geometry (square pyramidal) similar to that reported for the [Cu(HL)(NO_3_)_2_] complex that also contains the HL ligand and nitrate anions.^92^ A copper complex containing a ligand (*N*-2-ethanolpicolylamine) similar to HL was also reported recently and shows a square pyramidal structure similar to that observed in **1**.^111^

In **1**, both HL and a chlorido ligand occupy the basal plane while the second chlorido ligand is present in the apical position (Figure 2). The HL ligand binds in a meridional fashion to the Cu(II) ion via the pyridine (N12), amine (N1), and alcohol (O1) groups as observed for a Zn(II) complex synthesized with the same ligand.^112^ This coordination environment differs from that observed in a compound with the similar ligand HBPA [(2-hydroxybenzyl)(2-pyridylmethyl)amine], in which a phenol group replaces the alcohol group of HL. In the [Cu(HBPA)Cl_2_] compound,^113^ the phenol group occupies the apical position. This difference may be caused by the formation of a six-membered ring in [Cu(HBPA)Cl_2_] and a five-membered ring in **1**. The Cu(1)–Cl(2) bond distance is longer than the Cu(1)–Cl(1) distance due to Jahn–Teller distortion.

On the contrary, complex **2** adopts a hexacoordinated Cu(II) center with a distorted octahedral geometry (Figure 2). The Cu(II) ion is coordinated to two molecules of HL with two perchlorate anions present as counterions. In comparison to **1**, the ligand HL in **2** shows a facial coordination mode. The pyridine nitrogen atoms (N1 and N1′) and amine nitrogen atoms (N2 and N2′) form the equatorial plane. The axial axes are occupied by the alcohol groups (O1 and O1′) similar to the locations of phenol groups observed in [Cu(HBPA)_2_]Cl_2_.^114^ The Cu–O bonds in **2** are ∼0.3 Å longer than that in **1** due to the Jahn–Teller effect (Table 1). In comparison to the phenol groups containing the [Cu(HBPA)_2_]Cl_2_ complex, the Cu–O distance in **2** is ∼0.22 Å shorter.^114^ This difference may be attributed again to the formation of a five-membered ring in the former and a six-membered ring in the latter.

**Table 1:** Main Bond Distances (angstroms) and Angles (degrees) for Compounds **1** and **2**

complex **1**		complex **2**	
bond lengths			
Cu(1)–N(1)	1.9956(10)	Cu(1)–N(1)	2.034(2)
Cu(1)–N(2)	2.0139(10)	Cu(1)–N(2)	2.041(2)
Cu(1)–O(1)	2.0155(9)	Cu(1)–O(1)	2.322(2)
Cu(1)–Cl(1)	2.2251(4)	Cu(1)–N(1′)	2.034(2)
Cu(1)–Cl(2)	2.5969(5)	Cu(1)–N(2′)	2.041(2)
		Cu(1)–O(1′)	2.322(2)
angles			
N(1)–Cu(1)–N(2)	82.79(4)	N(1)–Cu(1)–N(2)	82.09(9)
N(1)–Cu(1)–O(1)	152.39(4)	N(1′)–Cu(1)–N(1)	180.0
N(2)–Cu(1)–O(1)	82.13(4)	N(1′)–Cu(1)–N(2)	97.91(9)
N(1)–Cu(1)–Cl(1)	97.92(3)	N(1′)–Cu(1)–N(2′)	82.09(9)
N(2)–Cu(1)–Cl(1)	169.59(3)	N(1)–Cu(1)–N(2′)	97.91(9)
O(1)–Cu(1)–Cl(1)	92.94(3)	N(2)–Cu(1)–N(2′)	180.00(9)
N(1)–Cu(1)–Cl(2)	103.96(3)	N(1′)–Cu(1)–O(1)	88.74(9)
N(2)–Cu(1)–Cl(2)	85.40(3)	N(1)–Cu(1)–O(1)	91.26(9)
O(1)–Cu(1)–Cl(2)	97.73(3)	N(2)–Cu(1)–O(1)	79.27(9)
Cl(1)–Cu(1)–Cl(2)	104.415(16)	N(2′)–Cu(1)–O(1)	100.73(9)
		N(1′)–Cu(1)–O(1′)	91.26(9)
		N(1)–Cu(1)–O(1′)	88.74(9)
		N(2)–Cu(1)–O(1′)	100.73(9)
		N(2′)–Cu(1)–O(1′)	79.27(9)
		O(1)–Cu(1)–O(1′)	180.0

### Characterization of Cu(II) Complexes in Solution: Potentiometric Titration and ESI-(+)-MS and ESI-(+)-MS/MS

The crystallographic data for complex **1** reveal the presence of just one ligand coordinated to the Cu(II) ion. In comparison, there are two ligands coordinated to Cu(II) in complex **2**. To determine the chemical nature of the species in solution, mainly at physiological pH, potentiometric titration studies are performed for the ligand HL and complexes **1** and **2**.

The data for the ligand HL (Table 2) in the pH range of 3.0–11.0 show only one protonation/deprotonation equilibrium involving the amine group with a p*K*_a_ of 8.11. It is almost 1 pH unit higher than that observed in the tridentate ligand bis(2-pyridylmethyl)amine (p*K*_a_ = 7.28).^115^ The p*K*_a_ of the pyridine group is <3.^115^ Thus, in the pH range of 3.0–8.0, the majority of the ligand is in the cationic form (H_2_L^+^).^116^

**Table 2:** Data (p*K*_a_ values) of the Titrated Compounds

quotient^a^	log *K*
Ligand HL	
[H_2_L^+^]/[HL][H^+^]	8.11
Compound **1**	
[[Cu(HL)(H_2_O)*_n_*]^2+^]/[Cu^2+^][HL]	6.91
[[Cu(H_2_L)(H_2_O)*_n_*]^3+^]/[[Cu(HL)(H_2_O)_*n*_]^2+^][H^+^]	4.72
[[Cu(L)(H_2_O)*_n_*]^+^][H^+^]/[[Cu(HL)(H_2_O)*_n_*]^2+^]	8.43
[[Cu(L)(H_2_O)_*n*−1_(OH)]][H^+^]/[[Cu(L1)(H_2_O)_2_]^+^]	9.73
Compound **2**	
[[Cu(HL)_2_]^2+^]/[[Cu(HL)(H_2_O)_*n*_]^2+^][HL]	4.56
[[Cu(HL)(H_2_L)]^3+^]/[[Cu(HL)_2_]^2+^][H^+^]	7.87
[[Cu(HL)(L)]^+^][H^+^]/[[Cu(HL)_2_]^2+^]	9.49
[[Cu(L)_2_]][H^+^]/[[Cu(HL)(L)]^+^]	9.76

a *n* is the number of water molecules that can be coordinated to copper(II) in a water solution that is proposed to be 2 or 3.

In the literature, there are copper(II) compounds coordinated to tridentate ligands along with two water molecules.^117,118^ Furthermore, the UV–vis and electrochemical data support the presence of copper(II) ions with a pseudo-octahedral geometry. On the basis of these data, it is proposed that in a water solution, two or three water molecules interact with the copper center and replace the chlorido ligands.

For **1**, the measured formation constant (*K*_f_ = 8.13 × 10^6^) is lower than the value (*K*_f_ = 2.51 × 10^14^) reported for the compound [Cu(BMPA)],^116^ whose ligand contains one amine and two pyridine groups. Its further interaction with a second molecule of HL provides **2** with a *K*_f_ of 3.63 × 10^4^ and a log β of 11.5. These results show that the formation of **2** is more difficult than the creation of **1**. This behavior is attributed to the Jahn–Teller effect, a feature also observed for the compound [Cu(BMPA)_2_]^116^ (*K*_f_ = 3.98 × 10^4^). With regard to protonation–deprotonation equilibria, the p*K*_a_ associated with the amine group of the ligand HL (8.11) decreases significantly to 4.72 in the presence of Cu(II) ions for **1** and 7.87 for **2**. This implies that for **2** the amine group of the second ligand is primarily protonated below pH 7.87, which would result in the hydrolysis of the ligand (see below).

**Figure 3:**
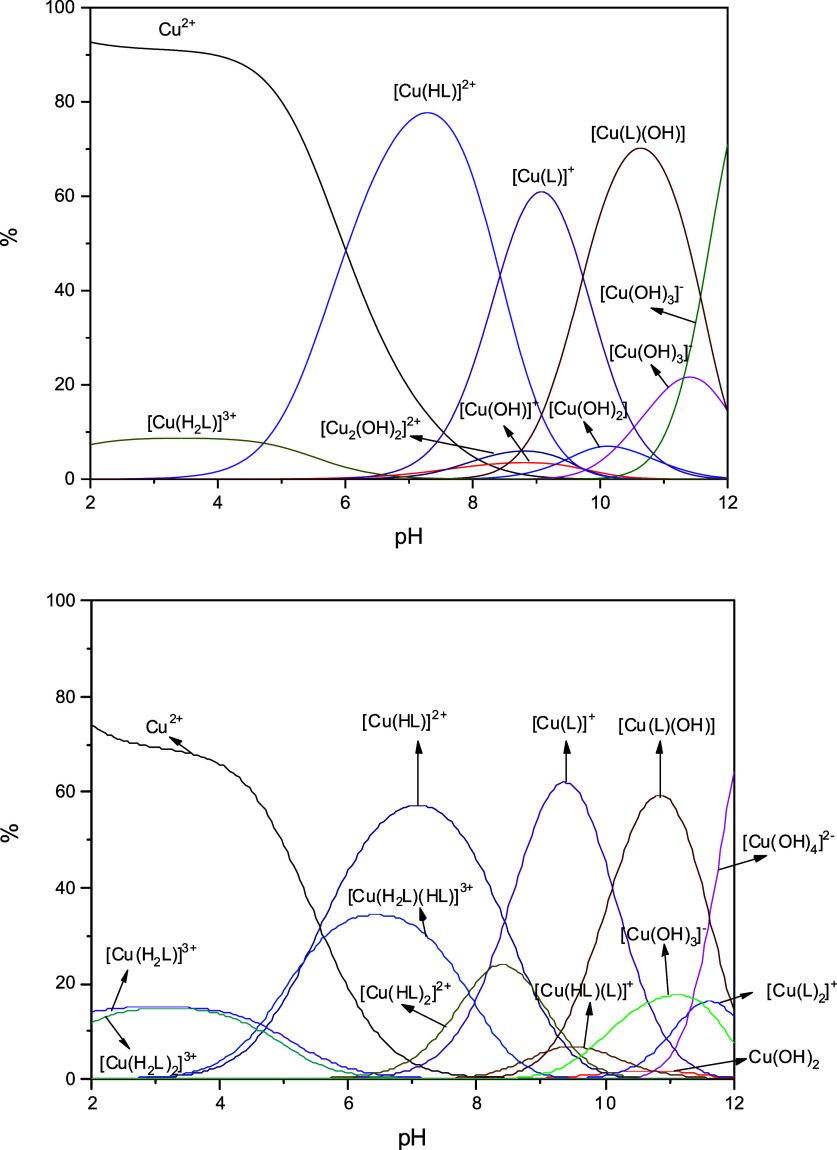
Relative concentrations of chemical species observed in solution in the pH range of 2–12 calculated from potentiometric titration data for complex **1** (top) and complex **2** (bottom). Water molecules coordinated to copper have been omitted for the sake of clarity. Ionic strength of KCl of 0.001 mol L^–1^, compound concentration of 0.01 mol L^–1^, and temperature of 25 °C.

The distribution of the species as a function of pH is shown in Figure 3. For **1**, between pH 6.9 and 8.4, the major species is [Cu(HL)(H_2_O)*_n_*]^2+^ (*n* = 1–3). At higher pH values, species [Cu(L)(H_2_O)*_n_*]^+^ (p*K*_a_ = 8.43) and [Cu(L)(H_2_O)_*n*−1_(OH)] (p*K*_a_ = 9.73) are formed. Above pH 8, a small amount of the Cu(II)-hydroxo species is also observed, as indicated in Figure 3 (top). On the contrary, the complex is unstable at acidic pH (<6). Thus, the data clearly show that the complex [Cu(HL)(H_2_O)_*n*_]^2+^ is stable and is the main species responsible for the catalytic activity (see below) at physiological pH.

The potentiometric titration of **2** shows a large number of chemical species in solution, which is explained by the equilibrium [Cu(HL)_2_]^2+^ + *n*H_2_O ⇌ [Cu(HL)(H_2_O)_*n*_]^2+^ + HL (bottom of Figure 3). In the pH range of 6.0–8.0, complex **1** (∼60%) is the main species present in solution and not the one observed in the solid state. Therefore, any biological activity in this pH range must be interpreted with caution.

The analysis of a 1:1 MeOH/H_2_O solution containing **1** by ESI-(+)-MS shows peaks at *m*/*z* 201, 264, 493, 529, and 830 (Figure S2). The base peak is at *m*/*z* 264, and its isotopic pattern agrees with that of the cation [Cu(HL)Cl]^+^. The peaks at *m*/*z* 493 and 529 refer to dinuclear species, whose isotopic patterns agree with the [Cu_2_(L)_2_Cl]^+^ and [Cu_2_(HL)(L)Cl_2_]^+^ compositions. The signal with the highest *m*/*z* (830) supports a trinuclear copper(II) cation, [Cu_3_(HL)_2_(L)Cl_4_]^+^ (see Figures S3–S6). It is proposed that the dinuclear and trinuclear species are clusters formed in the gas phase. MS/MS investigation of the peak at *m*/*z* 830 shows the formation of the signals at *m*/*z* 529 and 493 by the loss of neutral [Cu(HL)Cl_2_] and HCl molecules, respectively.

The ESI-(+)-MS investigation performed with **2** allowed us to identify four different species in the gas phase (Figure SI7). The base peak is at *m*/*z* 167 and refers to the protonated form of the ligand ([H_2_L]^+^). The peaks at *m*/*z* 394 and 494 are also relevant; i.e., the former is associated with [Cu(HL)(L)]^+^, while the latter with the [Cu(HL)_2_ClO_4_]^+^ cation (Figures S9 and S10). The MS/MS data show that it originates in the signal at *m*/*z* 394 {[Cu(HL)(L)]^+^} by the loss of a HClO_4_ molecule.

### Spectroscopic and Electrochemical Characterization

The IR spectra of **1** and **2** are compared to that of the free ligand in the region of 400–4000 cm^–1^ (Figure S11). For the ligand HL, characteristic bands of the aromatic group are observed at 1588, 1575, 1478, and 1441 cm^–1^ (νC=N and νC=C) and 761 cm^–1^ (δC–H). For **1** and **2**, these bands are located at 1607, 1568, 1477, 1441, and 772 cm^–1^ and 1613, 1574, 1491, 1429, and 772 cm^–1^, respectively. For complex **1**, a band at 2934 cm^–1^ is attributed to the methyl group. This band is observed at 2930 cm^–1^ for complex **2**. It also shows bands in the range of 1080–1120 cm^–1^ attributed to the perchlorate anion that acts as a counteranion.

Electronic spectra of the complexes were recorded in water, phosphate buffer (pH 7.4), and DMSO. The bands observed in the visible range of the two complexes are attributed to d–d transitions (Figure S12). The similarity between the spectra obtained in water and phosphate buffer (see Table 3) indicates that the coordination sphere of Cu(II) is similar in both solvents, although there is an appreciable amount of HPO_4_^2–^ and H_2_PO_4_^–^ ions in the buffered solution. The UV–vis data suggest a pseudo-octahedral geometry with a N_2_O_2_ coordination environment in the equatorial plane.^119^ A significant bathochromic effect is observed in DMSO for **1** (∼116 nm) and **2** (∼110 nm). The similarity between the spectra of **1** and **2** reinforces the suggestion that **2** transforms into **1** through ligand dissociation, in agreement with the potentiometric data.

**Table 3:** Yields, Elemental Analyses (C, H, and N), Conductivity Data (in methanol), UV–Vis Data, and Melting Points (mp) for Compounds **1** and **2**

	**1**	**2**
composition	C_9_H_14_Cl_2_N_2_OCu	C_18_H_28_Cl_2_N_4_O_10_Cu
yield (%) [mass (mg)]	52 (186)	37 (329)
%C found (calcd)	35.83 (35.83)	36.51 (36.34)
%H found (calcd)	4.62 (4.68)	4.69 (4.74)
%N found (calcd)	9.19 (9.29)	9.27 (9.41)
Λ_M_ (cm^2^ Ω^–1^ mol^–1^)	78.3^a^	189.7^a^
λ_max_ (nm) [ε (L mol^–1^ cm^–1^)]	595 (28)^b^	599 (15)^b^
	602 (11)^c^	600 (19)^c^
	716 (55)^d^	710 (19)^d^
mp (°C)	190	235

a With methanol as the solvent.

b With deionized water as the solvent.

c With a PBS solution (pH 7.4) as the solvent.

d With DMSO as the solvent.

**Figure 4:**
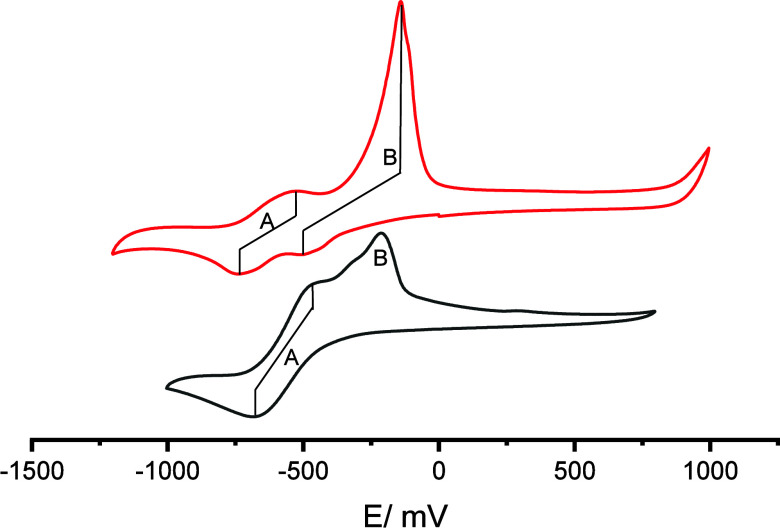
Cyclic voltammograms of complex **1** (red) and **2** (black) in H_2_O containing KCl (0.1 mol L^–1^) at 25 mV/s.

The cyclic voltammetry technique was employed to investigate the redox characteristics of compounds **1** and **2** (Figure 4). Compound **1** showed a quasi-reversible redox process at −0.628 V versus NHE associated with the Cu^2^/Cu^+^ redox couple (A in Figure 4). Another process (B in Figure 4) characterized by a low-intensity cathodic wave (*E*_pc_ = −0.506 V) and a high-intensity anodic current (*E* = −0.140 V) was also observed. According to the literature, it is attributed to the formation of a copper metallic film on the electrode surface (Cu^2+^ + 2e^–^ ⇌ Cu°).^120−122^

Complex **2** shows a quasi-reversible redox couple Cu^2+^/Cu^+^ at −0.566 V versus NHE. The anodic process related to the Cu°/Cu^2+^ couple is also occurring, but with lower current intensity when compared to that of **1**. As opposed to **1**, the cathodic wave associated with the formation of the copper film was not detected, probably due to the overlay of the redox process of complex **2**. The redox potentials observed for **1** and **2** are in the range observed for other copper complexes showing pseudo-octahedral geometry,^119^ in agreement with the UV–vis data.

### Copper and ROS Scavenging Properties of the Ligand (HL), **1**, and **2** with Respect to Rat Glioma C6 Cells

An increase in the concentration of transition metal ions such as copper and/or iron inside cells has been associated with metallic and/or oxidative stress disorders.^65,125,126^ They may result in the development of neurodegenerative disorders such as Parkinson’s disease and Alzheimer’s disease.^65,74,127^ To deal with these conditions, the development of organic molecules (metallophore) that can scavenge toxic metals has been pursued.^128,129^ An example of such a molecule is deferiprone (Figure 1), an iron chelator currently undergoing a phase 2 clinical trial for AD.^129,130^

As discussed above, the ligand HL can form different copper complexes, and their formation constants {*K*_f_ = 8.13 × 10^6^ for [Cu(HL)(H_2_O)*_n_*]^2+^, and *K*_f_ = 3.98 × 10^4^ for [Cu(HL)_2_]^2+^} show that they are thermodynamically stable. This observation prompted us to examine the efficacy of this simple organic molecule in reducing the metal-induced oxidative stress in neuronal glioma cells (C6). We explored whether the ligand and its complexes could mitigate oxidative stress when the cells are exposed to the following oxidative stress models: (i) Cu(I), (ii) hydrogen peroxide, (iii) superoxide anion, and (iv) hydroxyl radical.

**Figure 5:**
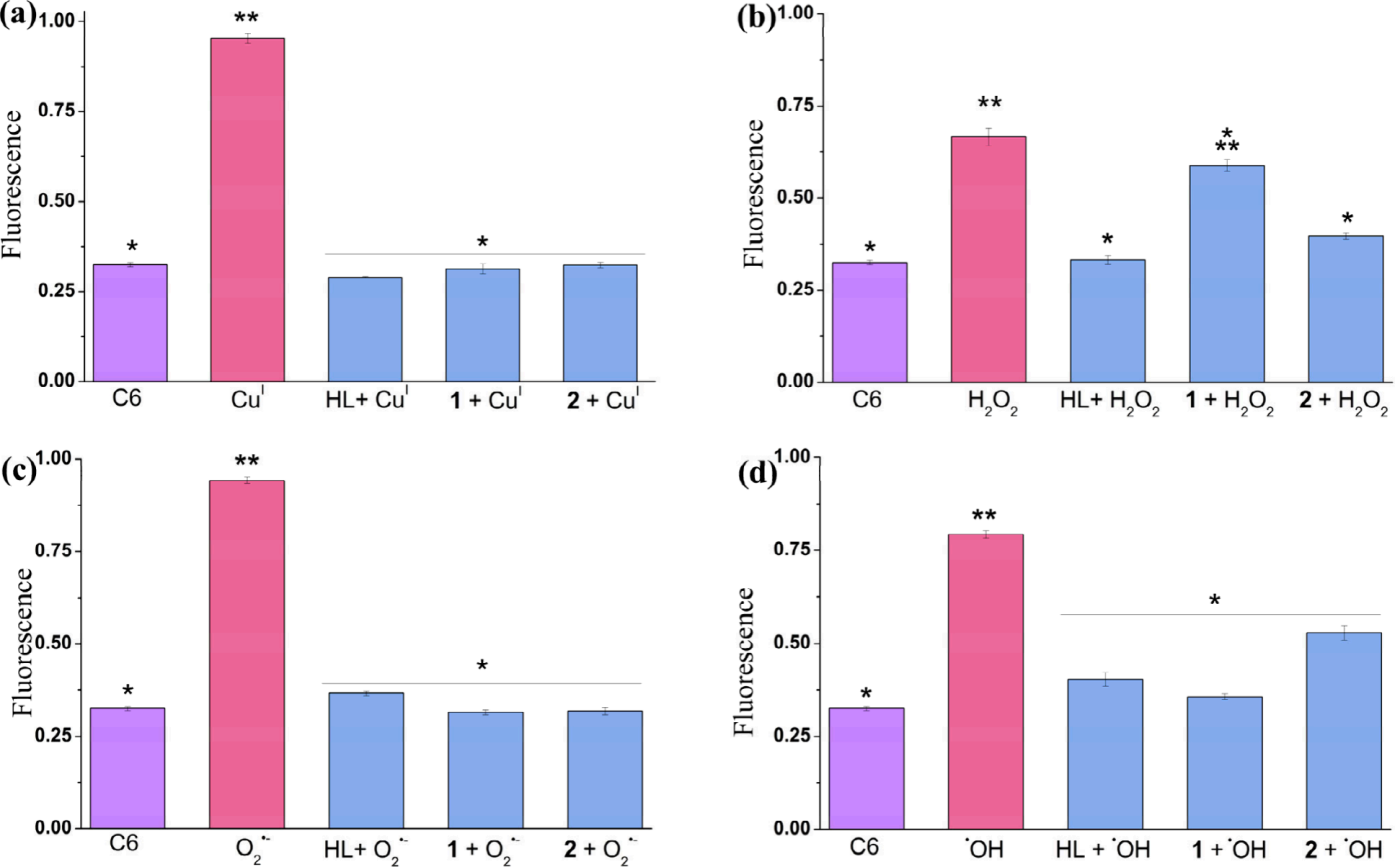
Change in the redox status of C6 cells (1 × 10^5^ cells/well) with or without pretreatment with the ligand HL, **1**, or **2** (as indicated) at 25 μmol L^–1^ and incubation for 24 h at 37 °C. To induce oxidative stress, the cells were washed and exposed to 25 μmol L^–1^ solutions of (a) CuCl, (b) H_2_O_2_, (c) O_2_^•–^, and (d) ^•^OH (0.4 μmol L^–1^ CuCl and 4 μmol L^–1^ H_2_O_2_) for 1 h. The control group (C6 cells) consisted of cells that were not subjected to any treatment. The results represent the mean ± the standard error of the mean of at least four independent experiments. Statistical analysis was performed using a one-way analysis of variance (ANOVA) with a *p* < 0.05 significance level. The fluorescence was normalized to 1.

Figure 5 illustrates the redox state of glioma C6 cells exposed to a 25 μmol L^–1^ CuCl solution. An analysis employing the probe dichlorodihydrofluorescein diacetate (DCFH-DA) shows that the treatment with Cu(I) changes the internal redox status of the cells. The fluorescence of the cells increased 3-fold after treatment with Cu(I), confirming an increase in ROS levels compared to the control. In contrast, no change in the cell redox status is observed when the cells are pretreated with the ligand HL, **1**, or **2**, prior to being exposed to Cu(I). Copper can be toxic to cells in several ways, i.e., by altering protein structure and function or generating ROS through the Fenton reaction.^131,132^ The latter involves the production of hydroxyl radicals as a result of the reaction of Cu(I) with hydrogen peroxide. Because the intrinsic hydrogen peroxide concentration inside mammalian cells can reach 10^–11^ to 10^–8^ mol L^–1^,^133^ it is proposed that the change in the redox status of cells is caused by the Cu(I)-induced Fenton reaction.^134^ Thus, the protection offered by the HL ligand can be associated with its capability to chelate Cu(I) and, thereby, reduce its toxicity by inhibiting ROS production. However, the underlying mechanisms for the protective effects of **1** and **2** are not trivial, because the ligand is already coordinated to copper. A plausible explanation could be that both complexes show antioxidant activities by decomposing ROS (H_2_O_2_, O_2_^•–^, and OH^•^), in a manner similar to that of specialized metalloenzymes such as superoxide dismutase (SOD) and catalase (CAT).^43^ The Cu–Zn SOD decomposes the superoxide anion inside the cytosol, and several copper complexes have been designed to mimic its activity.^62,135^ To the best of our knowledge, no natural copper metalloenzyme can decompose hydrogen peroxide (catalase activity) or the hydroxyl radical. However, a few synthetic copper complexes have been reported to exhibit such activities.^136,137^ In this study, the antioxidant properties of **1** and **2** are attributed to their ability to promote superoxide (SOD activity) and hydrogen peroxide (CAT activity) disproportionation reactions. Additionally, as discussed below, they can scavenge hydroxyl radicals.

The redox level of the C6 cells is also analyzed upon exposure to exogenous H_2_O_2_ (Figure 5b). HL and its complexes (**1** and **2**) provided distinct protection against H_2_O_2_, i.e., HL > **2** > **1**. Interestingly, the free HL ligand exhibited the best protective properties by maintaining the redox state at the basal level. It may be due to acting as a metallophore by coordinating to metal ions involved in the Fenton reactions inside the cell. In biological systems, the hydroxyl radical (^•^OH) formed via the Fenton reaction in the presence of H_2_O_2_ and transition metals (Fe, Cu, etc.) is widely recognized as one of the most potent radical species that induce oxidative modifications in proteins such as carbonylation, cross-linking, aggregation, and functional group loss.^72^ The scavenging of metal ions in biological systems could inhibit the Fenton reaction. This property is supported by the observation that the ligand can protect cells stressed with Cu(I).

Organic molecules have been reported to act as antioxidant agents through various mechanisms, such as proton/electron transfer and metal chelation.^138−140^ This property depends on the chemical structures of the antioxidant and oxidizing agents. For instance, gallic acid can neutralize reactive species by the proton-coupled electron transfer mechanism, in which the received electron is stabilized by the phenol group.^141^ Segat and co-workers have demonstrated that the ligand (2-hydroxybenzyl-2-pyridylmethyl)amine (HBPA) decomposes the DPPH radical in EtOH due to the presence of phenol groups, which can donate a proton to the DPPH radical.^142^ We have observed that both **1** and **2** exhibit catalase activity, i.e., **1** > **2** (see below). However, the results with the cells demonstrate the opposite effect (**2** > **1**), as shown in Figure 5B. This surprising observation can be explained by the potentiometric titration data, which suggest that at the cell pH (7.4) complex **2** transforms into **1** and releases a ligand molecule to create a more potent antioxidant system composed of **1** and HL.

The antioxidant protection in neuronal glioma C6 cells is also evaluated in the presence of a superoxide radical anion. As shown in Figure 5C, the ligand HL and complexes **1** and **2** display comparable activities by maintaining the redox state at the basal level. The protection exhibited by compounds **1** and **2** may be related to their SOD-like activity (see below).

The cells are also exposed to a fourth stress condition using a hydroxyl radical (Figure 5d). Even in the presence of this potent oxidant, the compounds show a significant antioxidant effect (HL ∼ **1** > **2**). EPR experiments demonstrate that **1** and **2** can decrease the concentration of hydroxyl radicals, which explains their protective effect on the cells (see below).

Collectively, the data indicate that **1** and **2** and even the free HL ligand can mitigate the change in the redox status of the C6 cells exposed to Cu(I), H_2_O_2_, O_2_^•–^, and ^•^OH species. As shown below, the antioxidant properties of **1** and **2** are attributed to their catalase and superoxide dismutase activities and their ability to scavenge the hydroxyl radical.

With regard to the protective effect of the HL ligand, its coordination with metal ions inside the cells decreases the availability of such species and therefore decreases the likelihood of the Fenton reactions. Additionally, as observed with copper, HL can form coordination compounds with antioxidant activity, providing double protection by simultaneously avoiding metal and oxidative stress conditions.

### Antioxidant Activities of Complexes **1** and **2** against H_2_O_2_, O_2_^•–^, and ^•^OH

The protection of rat glioma C6 cells against oxidative stress offered by compounds **1** and **2** can be attributed to their CAT and/or SOD-like activities. Thus, kinetic, spectroscopic, and theoretical studies are performed to test their activities.

#### Superoxide dismutase (SOD) Activity: Kinetic, EPR, and Theoretical Studies

Due to the antioxidant protection shown by compounds **1** and **2** on C6 cells, we investigated their intrinsic SOD mimicking activities. Initially, interactions of complexes **1** and **2** with the superoxide anion are studied kinetically by employing an NBT assay. In this assay, these compounds compete with NBT to react with the superoxide anion generated by the xanthine/xanthine oxidase system.^61^Table 4 presents the kinetic data for SOD activities of different Cu(II) compounds, including **1** and **2** (Figures S13 and S14). According to Green and colleagues,^143^ Cu(II) compounds that exhibit the highest SOD-like activity have shown *k*_cat_ values of ∼10^7^ M^–1^ s^–1^. Therefore, compound **1** with a *k*_cat_ of 3.1 × 10^7^ is among the most active compounds reported in the literature. In comparison to [Cu_2_(indo)_4_(DMF)_2_] (IC_50_ ∼ 2–25 μmol L^–1^), which is employed as an anti-inflammatory drug in veterinary medicine,^144,145^ compound **1** is ∼20 times better.

**Table 4:** Superoxide Dismutase Activities of Compounds **1** and **2**, CuCl_2_, Other Copper Complexes, and the Natural Superoxide Dismutase (SOD) Enzyme

compound^b^	IC_50_ (μmol L^–1^)	*k*_cat_^a^ (M^–1^ s^–1^)	log *k*_cat_	ref
**1**	0.108 ± 0.065	3.1 × 10^7^	7.5	this work
**2**	0.326 ± 0.042	1.0 × 10^7^	7.0	this work
CuCl_2_·2H_2_O	0.910 ± 0.191	ND^e^	ND^e^	(148)
[Cu(BCEN)]Cl_2_·2H_2_O	0.179 ± 0.017	ND^e^	ND^e^	(148)
[Cu(BPAP)H_2_O]Cl_2_	0.168 ± 0.030	ND^e^	ND^e^	(148)
[Cu(BPAH)H_2_O]Cl_2_	0.129 ± 0.033	ND^e^	ND^e^	(148)
[Cu^II^(H_2_BPClNOL)(Cl)]^2+^	0.181 ± 0.016	7.07 × 10^6^	ND^e^	(150)
[Cu(L2)_2_](ClO_4_)_2_	0.150	ND^e^	ND^e^	(151)
[Cu(HPClNOL)]	0.430	7.7 × 10^6^	ND^e^	(61)
[Cu(salH)_2_(H_2_O)_2_]	1.230	ND^e^	ND^e^	(152)
[CuCF_3_PyN_3_]^2+^	0.133(1)	2.0 × 10^7^	7.3	(143)
[CuPuPhePy]^2+^	0.270	5.28 × 10^7^	7.7	(153)
[Cu(PuPy)]^2+^	0.603	2.6 × 10^7^	7.4	(154)
[Cu_2_(indo)_4_(H_2_O)_2_]	1.31	ND^e^	ND^e^	(145)
BeSOD	0.042 ± 0.010			(146)
CuZn-SOD^c^	0.15			(153)
CuZn-SOD^c^	0.03	1.3 × 10^9^	9.1	(155)
CuZn-SOD^d^	0.0026			(156)

a *k*_cat_ = *k*_NBT_ × [NBT]/IC_50_, where *k*_NBT_ = 5.94 × 10^4^ M^–1^ s^–1^ and [NBT] = 5.6 × 10^–5^.

b BCEN = 3,3′-[ethane-1,2-diylbis(azanediyl)]dipropanamide. BPAP = 3,3′-(piperazine-1,4-diyl)dipropanamide. BPAH = 3,3′-(1,4-diazepane-1,4-diyl)dipropanamide.

c IC_50_ was determined by employing the xanthine/xanthine oxidase-mediated reduction of NBT.

d IC_50_ was determined by measuring the inhibition of the photoreduction of NBT.

e Not determined.

Some key features of Cu(II) compounds that allow them to attain high SOD-like activity include (a) the Cu(I)/Cu(II) redox potential (850 to −160 mV vs NHE), (b) flexible ligands that can facilitate the change in the metal geometry (square planar/tetrahedral), (c) an accessible coordination site, (d) an equatorial ligand field of medium strength, and (e) stabilization of the Cu–superoxide interaction through hydrogen bonding.^56,146^

Although the redox potential shown by **1** is out of the range for the promotion of superoxide reduction/oxidation, a direct correlation cannot be established, because the redox potential is relevant when considering the outer sphere electron transfer process. When superoxide coordinates to the metal ion, an inner electron transfer can occur, changing the redox potentials of the metal/superoxide ions and facilitating the redox process.^147^ Additionally, several SOD mimics have been reported whose redox potentials are not thermodynamically suitable for promoting the superoxide redox reaction under the same conditions.^148,149^

With regard to the structural features, the bipodal ligand HL is flexible enough to allow the change in the geometry around the metal center. Furthermore, the alcohol group can form a hydrogen bond and stabilize the Cu–superoxide interaction. The formed compound also shows an unsaturated coordination environment or labile ligands (H_2_O) that allow direct interaction of the superoxide anion with the metal center. This mode of binding is also proposed by the DFT calculations discussed below. This feature is not present in **2**, but the potentiometric titration data show that it undergoes hydrolysis of one of its ligands and exists mainly as complex **1** (∼60%) at physiological pH. Thus, the SOD activity shown by **2** is actually due to the generation of **1** in solution.

**Figure 6:**
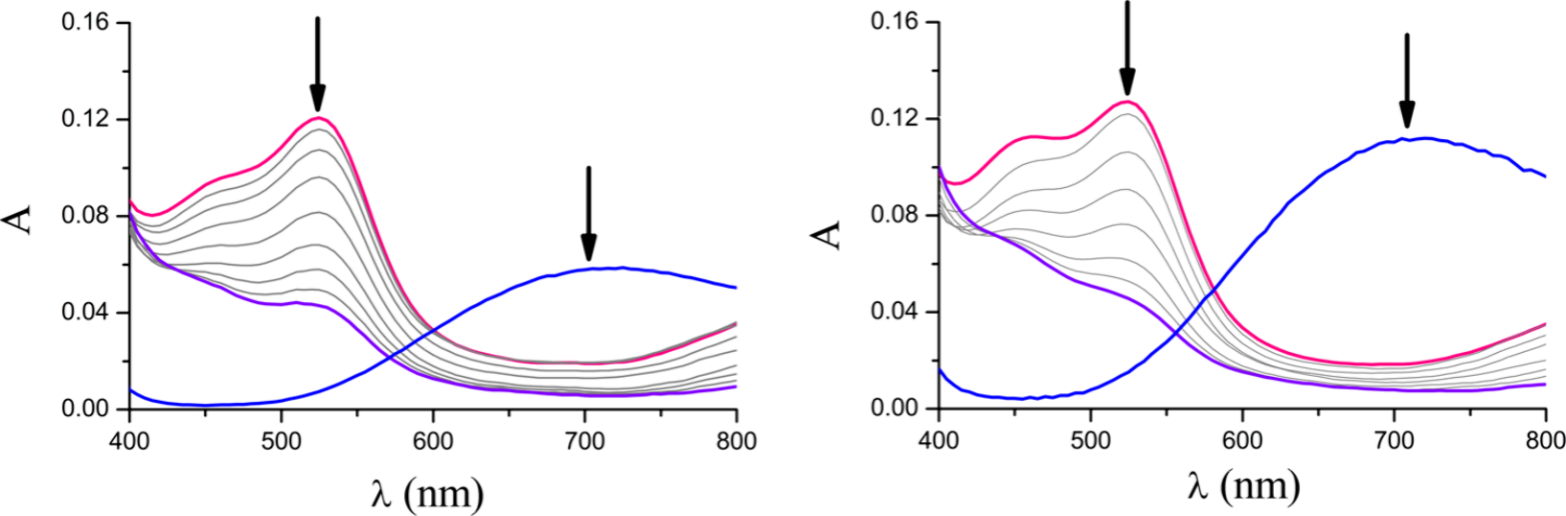
UV–vis measurements of the interaction between KO_2_ (no absorbance in this range) and complex **1** (left) or **2** (right). Blue lines refer to the isolated complexes, which show d–d bands in the visible range. The red line refers to the initial interaction between KO_2_ and Cu(II) complexes, which decreases over time.

**Figure 7:**
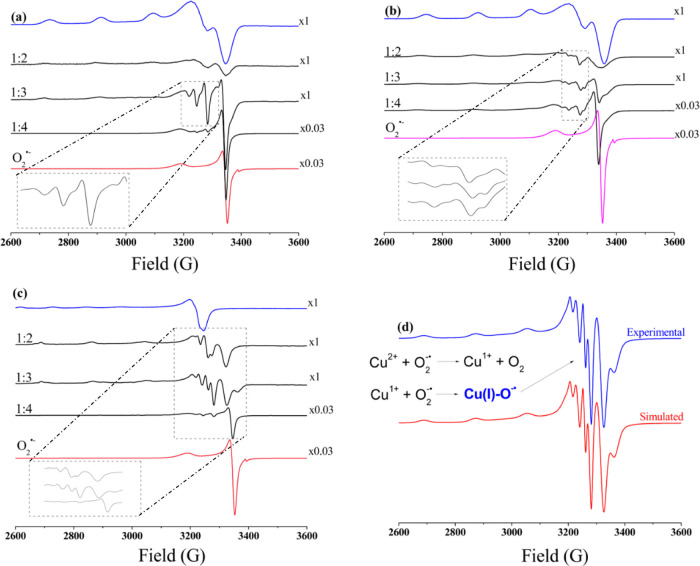
EPR studies of the interaction of (a) **1**, (b) **2**, and (c) CuCl_2_ with the superoxide anion (O_2_^•–^) in DMSO at 120 K employing different Cu:O_2_^•–^ ratios, as indicated. (d) Experimental and simulated EPR spectra considering a mixture of paramagnetic species observed in the CuCl_2_ + O_2_^•–^ reaction at a 1:3 ratio in DMSO. EPR parameters for species A [Cu(II)]: *g*_*x*_ = 2.06136, *g_y_* = 2.04877, *g*_*z*_ = 2.2636, *A*_*x*_ = 14.6601 G, *A*_*y*_ = 8.47669 G, and *A*_*z*_ = 182.331 G. EPR parameters for species B [Cu(I)–O_2_^•–^]: *g*_*x*_ = 2.24698, *g*_*y*_ = 1.99616, *g*_*z*_ = 2.08677, *A*_*x*_ = 0.0 G, *A*_*y*_ = 0.0 G, and *A*_*z*_ = 21.0538 G.

The interaction between the metal center and the superoxide ion is investigated by performing UV–vis (Figure 6) and EPR (Figure 7) experiments in a DMSO solution. The spectra of the complexes in DMSO display a bathochromic shift in relation to that measured in a water/buffer solution. They show a maximum absorption around 710 nm that reveals that the degree of splitting of the d orbitals in DMSO is lower than in water. Upon the addition of O_2_^•–^, the original spectrum related to the copper complexes changed immediately (Figure 6), i.e., a decrease in the d–d band in the visible range. Concomitantly, a new band appears close to the UV region (∼430 nm). On the basis of the previously reported data, the new band around 430 nm is attributed to a superoxide → Cu^2+^ ligand-to-metal charge transfer (LMCT) process.^147,157−159^ However, this band is not stable and decreases rapidly. Because these compounds exhibit SOD activity, this spectral behavior may be a result of an electron transfer process in which Cu^2+^ is reduced to Cu^+^ and the superoxide anion is oxidized to molecular oxygen.

To confirm this redox process, EPR experiments are performed by freezing the solution at 120 K after reaction for 30 s. Panels a and b of Figure 7 show the EPR spectra of the pure complexes and superoxide as well as of mixtures at different Cu:O_2_^•–^ ratios. While the EPR spectrum of the pure superoxide anion shows a typical axially distorted signal with a *g*_∥_ of 2.11 and a *g*_⊥_ of 2.01 as described in the literature,^160^ the spectra of the copper species are typical of mononuclear Cu(II) species showing rhombic symmetry with the following values: *g*_*x*_ = 2.063, *g*_*y*_ = 2.095, and *g*_*z*_ = 2.263; *A*_*x*_ = 0.447 G (1.147 × 10^–8^ cm^–1^), *A*_*y*_ = 0.466 G (1.196 × 10^–8^ cm^–1^), and *A*_*z*_ = 177.251 G (165.7 × 10^–4^ cm^–1^) for complex **1**; *g*_*x*_ = 2.058, *g*_*y*_ = 2.090, and *g*_*z*_ = 2.254; *A*_*x*_ = 0.482 G (1.237 × 10^–8^ cm^–1^), *A*_*y*_ = 1.262 G (2.239 × 10^–8^ cm^–1^), and *A*_*z*_ = 178.816 G (167.2 × 10^–4^ cm^–1^) for complex **2** (see Figures S15 and S16). Considering the *g*_∥_ and *A*_∥_ values, the EPR parameters indicate Cu(II) centers with square-based pyramidal structures.^119^ The distortion of the equatorial bonds from the planar arrangement has been evaluated by the *g*_∥_/*A*_∥_ ratio. Values between 105 and 135 cm^–1^ indicate a square planar arrangement of the equatorial plane, and values between 150 and 250 suggest a tetrahedral distortion.^161,162^ The values for **1** and **2** are 136.5 and 134.8, respectively, agreeing with a planar arrangement of the equatorial plane.

At a 1:2 Cu:O_2_^•–^ ratio, the intense signal associated with the superoxide anion disappears completely and the intensity of the Cu^2+^ signal decreases significantly in both parallel and perpendicular regions, indicating that the Cu(II) ion is being reduced to Cu(I), in agreement with the results of the UV–vis experiments. Such behavior is also supported by theoretical calculations (see below). When the ratio reaches 1:3, signals associated with the superoxide anion begin to dominate the spectrum, but a new pattern of narrow lines appears at *g* ∼ 2.0. This new set of lines is tentatively associated with a superoxide anion with a symmetry lower than that of the axial one, which can be generated by the coordination of the superoxide anion to Cu(I). This interaction creates the paramagnetic species Cu(I)-superoxide, an intermediate species proposed in the catalytic cycle of the SOD enzyme.^163^ This signature remains even at a ratio of 1:4, although at this proportion the spectrum is dominated by the axially distorted superoxide signal. This behavior was observed for both copper complexes but is clearer for compound **1**. Several attempts to simulate such spectra were unsuccessful, probably due to the contribution of diverse species for the spectral characteristics. This intriguing pattern was reported previously by Kimura^164^ and Lippard^165^ in reactions involving Cu(salicylate)_2_ complexes and superoxide. While Kimura and co-workers proposed the formation of the paramagnetic equilibrium complex Cu(I)–O_2_^•–^ ⇌ Cu(II)–O_2_^2–^ in the superoxide decomposition catalytic cycle promoted by [Cu(salicylate)_2_], Lippard and co-workers suggested that the EPR spectrum was actually a mixture of those of Cu(Sal) and [Cu(Sal)_2_]^2–^. Recently, we have described a similar behavior between two other copper complexes and the superoxide anion and assigned the new signature around *g* ∼ 2.0 as an intermediate species Cu(I)–O_2_^•–^.^137^ It is important to highlight that there is a relation between the time in which these new signals appear in the EPR spectra and the decrease in the intensity of the UV–vis signal attributed to the superoxide → Cu(II) LMCT. Surprisingly, when doing a background study using CuCl_2_·2H_2_O salt instead of complex **1** or **2**, we found a very well-defined spectrum containing narrower lines arose, simulated (Figure 7d) as containing Cu(I)–O_2_^•–^ (major amount, species B) and Cu(II) (minor amount, species A), and resembles that observed for the interaction between complexes **1** and **2** and O_2_^•–^.

**Figure 8:**
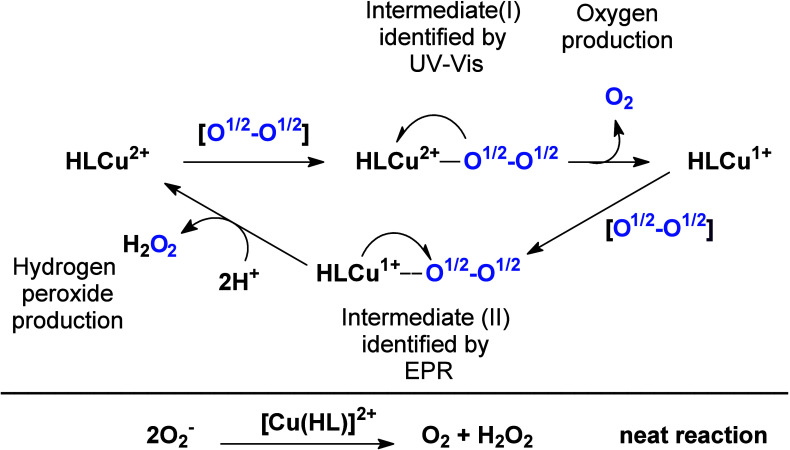
Proposed mechanism for the SOD activity shown by compound **1** and indication of intermediate species identified by UV–vis and EPR spectroscopies.

Thus, both electronic and EPR spectroscopies indicate the coordination of the superoxide anion to the copper ion associated with an electron transfer process. A plausible mechanism for the reaction of **1** with O_2_^•–^ is shown in Figure 8. According to the results of the UV–vis, EPR, and kinetic studies, the superoxide anion coordinates to the Cu(II) center (intermediate I). An electron transfer from the superoxide to the Cu(II) ion generates Cu(I) and molecular oxygen, O_2_. In the next step, a second superoxide anion coordinates to the Cu(I) species (intermediate II) that was observed by EPR spectroscopy. This interaction results in another electron transfer process [Cu(I) → O_2_^•–^], forming Cu^2+^ and peroxide species.

**Figure 9:**
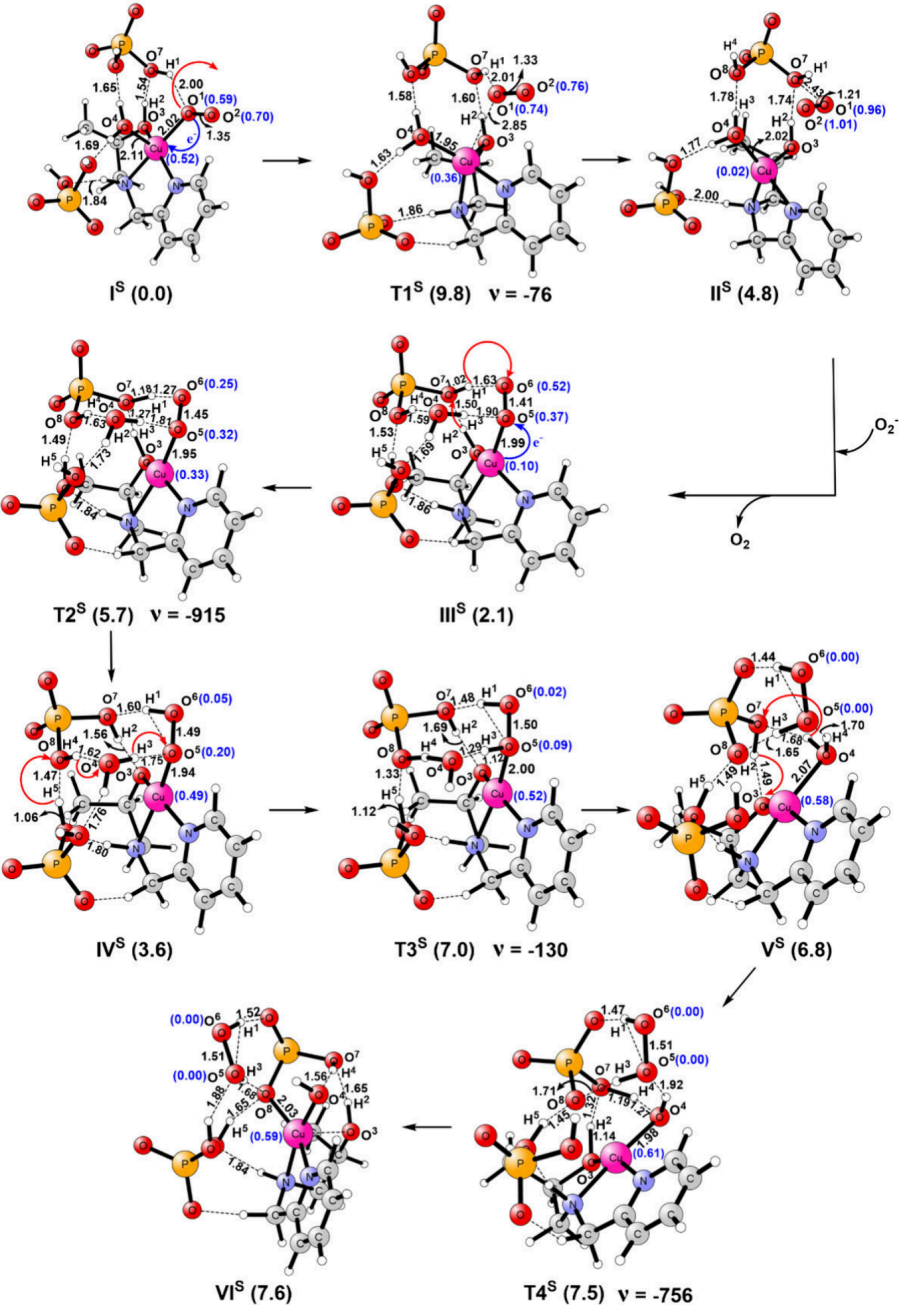
Structures of the SOD mechanism investigated using DFT calculations with key distances (in angstroms), electronic spins (blue), relative energies (in kilocalories per mole), and imaginary frequencies (in inverse centimeters).

This mechanism is also investigated using DFT calculations for complex **1** by modeling the experimental conditions (phosphate buffer and pH 7.8). In the reactant (**I**^**S**^), Cu(II) is in the pentacoordinated state and bound to the ligand, the O_2_^•–^ ion, and a water molecule in a distorted square pyramidal geometry (Figure 9). Additionally, two H_2_PO_4_^–^ ions and one water molecule form a hydrogen bonding network with the [Cu(II)(HL)]^2+^ complex. **I**^**S**^ is the lowest-energy structure among five different optimized conformations shown in Figure S17a. All other conformations with different modes of O_2_^•–^ and water coordination and hydrogen bonding patterns are higher in energy by 2.0–13.0 kcal/mol in the gas phase. In **I**^**S**^, the O_2_^•–^ ion (electron spin of 1.29*e*) is coordinated to the Cu(II) ion in an end-on manner (Cu–O^1^ bond length of 2.02 Å in Figure 9). Such a coordination mode has been proposed by structural, spectroscopic, and theoretical methods^157,166−168^ and considered to be an important intermediate in oxidation reactions promoted by copper.^159,166,169−171^ As suggested experimentally, the Cu(II)–O_2_^–^ interaction triggers an inner sphere electron transfer from O_2_^•–^ to the Cu(II) ion through the transition state (**T1**^**S**^). This electron transfer occurs with a barrier of 9.8 kcal/mol in the rate-determining step (RDS). It is clearly indicated by an increase in Mulliken spin densities to 0.74*e* and 0.76*e* on O^1^ and O^2^, respectively, and a reduction in electron density to 0.36*e* on the Cu ion in **T1**^**S**^. The computed barrier is in excellent agreement with the measured value of 10.2 kcal/mol converted from the first-order rate constant of 3.1 × 10^7^ using the Arrhenius equation. This process leads to the generation and release of dioxygen molecules in **II**^**S**^. In **II**^**S**^, the vacant coordination site is occupied by the water molecule, and it is 4.8 kcal/mol endothermic from **I**^**S**^. In the **I**^**S**^ → **II**^**S**^ transformation, the geometry of the Cu complex changes from square planar to tetrahedral. In the next step, the second O_2_^•–^ ion replaces the water molecule and coordinates to the previous [Cu(I)(HL)]^+^ complex in an end-on fashion to form a new complex (**III**^**S**^). The electronic spins of 0.10*e* on Cu and 0.89*e* on O_2_^•–^ indicate the Cu(I)–O_2_^•–^ nature of the complex. The generation of **III**^**S**^ is exothermic by 2.7 kcal/mol from **II**^**S**^; i.e., 2.1 kcal/mol is endothermic from **I**^**S**^. From **III**^**S**^, an electron-coupled proton transfer created the [Cu(II)(L1)(OOH)]^+^ intermediate (**IV**^**S**^) through a transition state (**T2**^**S**^) with a barrier of 5.7 kcal/mol. The proton (H^2^) in this process is donated through phosphate buffer by the -O^3^H^2^ group of the ligand to the terminal O^6^ atom of the Cu(I)-superoxide moiety. The interaction of this group with Cu(I) enhanced its acidity. From **IV**^**S**^, a proton transfer from the buffer to the Cu(II)–OOH^–^ species through a network of hydrogen bonds produced H_2_O_2_ (**V**^**S**^). This process took place with a barrier of 7.0 kcal/mol. From **V**^**S**^, a proton transfer from the Cu(II)-bound water molecule to [Cu(II)(L)]^+^ via the phosphate buffer regenerated the active complex [Cu(II)(HL)]^2+^ (**VI**^**S**^) with a small barrier of 0.7 kcal/mol, i.e., 7.5 kcal/mol from **I**^**S**^. The overall reaction was endothermic by 7.6 kcal/mol.

#### Catalase Activity: Kinetic, UV–Vis, ESI-(+)-MS, EPR, and Theoretical Studies

The metal–peroxide interaction has been reported in natural and synthetic (bio)chemistry.^172−175^ In natural copper-containing systems, relevant mono/multi-copper (hydro)peroxide intermediates have been spectroscopically and structurally characterized in reactions between dioxygen and Cu(I). Synthetic systems have revealed that Cu(I) species react with O_2_ to generate Cu(II)-superoxide, which can undergo protonation/complexation or even oxidation to produce Cu(II)-(hydro)peroxide, Cu(III)-peroxide, Cu(II)-peroxide-Cu(II), or Cu(III)-dioxide-Cu(III) species.^175−179^ However, instead of stabilizing them, copper centers can undergo subsequent reduction to release molecular oxygen and form Cu(I) species. If several oxidation/reduction steps occur, a typical catalase behavior can be established, although there are no such Cu-containing natural systems.

**Figure 10:**
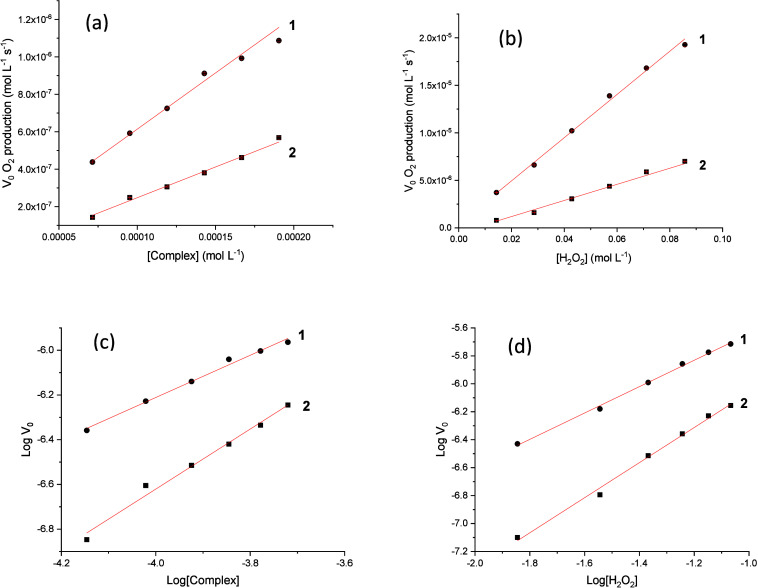
Plots of the initial rates of oxygen production catalyzed by complexes **1** (●) and **2** (■) as a function of the concentration of (a) the complex and (b) H_2_O_2_ and (c) log *V*_0_ vs log[complex] and (d) log *V*_0_ vs log[H_2_O_2_].

Because complexes **1** and **2** had a protective effect on the neuronal C6 cells exposed to hydrogen peroxide, their catalase activities are investigated through kinetic measurements (Figure S18) using the pseudo-first-order treatment. Figure 10a shows the rates of oxygen production as a function of the concentrations of **1** and **2**, while Figure 10b shows the dependence of oxygen production on H_2_O_2_ concentration. It is observed that the level of oxygen production is higher for complex **1**. Panels c and d of Figure 10 are used to obtain the reaction order with respect to the complex and H_2_O_2_ concentrations, respectively. The data show that the kinetics is first-order in relation to **1** (order of 0.94 ± 0.04) and H_2_O_2_ (order of 0.94 ± 0.02), resulting in a rate law *k*_obs_[**1**][H_2_O_2_], where *k*_obs_ = 0.061 ± 0.008 L mol^–1^ s^–1^.

**Scheme 2:**
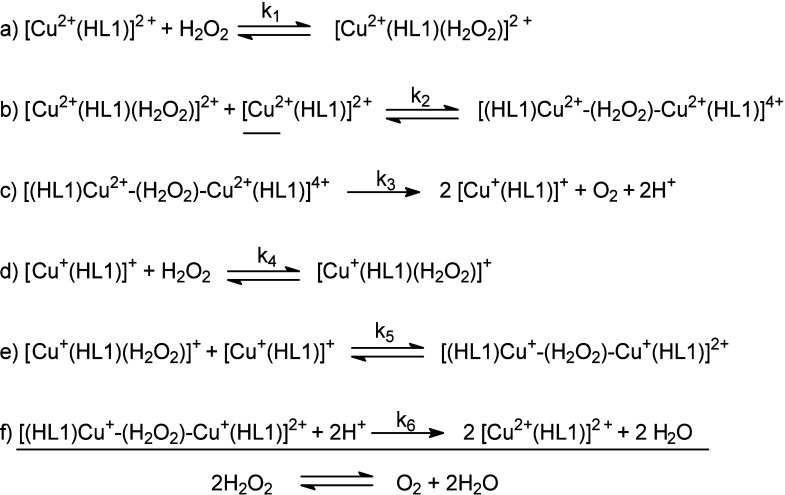
Proposed Elementary Steps Involved in H_2_O_2_ Disproportionation Catalyzed by [Cu(HL)Cl_2_] (**1**)

In comparison to the SOD activity, the CAT activity involves the transfer of two electrons to form O_2_ and H_2_O (2H_2_O_2_ → O_2_ + 2H_2_O). Thus, hydrogen peroxide needs to either accept or donate two electrons. On the contrary, the redox chemistry associated with copper complexes usually involves only one electron related to the Cu^2+^/Cu^+^ transformation. The kinetic data show that the reaction is first-order with respect to the copper complex and peroxide. Thus, the reaction mechanism should be more complex than a 1:1 direct reaction involving **1** and H_2_O_2_ due to the fact that H_2_O_2_ requires two electrons and a molecule of **1** can provide only one electron. A plausible mechanism for O_2_ production involving elementary reactions (Scheme 2) is proposed using a steady-state approach (details in the Supporting Information). On the basis of this mechanism, *k*_obs_ = *k*_1_; therefore, reaction a is the rate-limiting reaction.

In comparison to those of **1**, kinetic data for **2** indicate a non-integer reaction order with respect to the complex (order of 1.34 ± 0.07) and H_2_O_2_ (order of 1.25 ± 0.05), resulting in the rate law *k*_obs_[**2**]^1.3^[H_2_O_2_]^1.2^. The fractional order suggests that the concentration of the active species is not the initial concentration. The potentiometric titration study supports this possibility because at pH 7.4 there are at least three different species in solution with complex **1** as the major contributor (∼60%).

**Figure 11:**
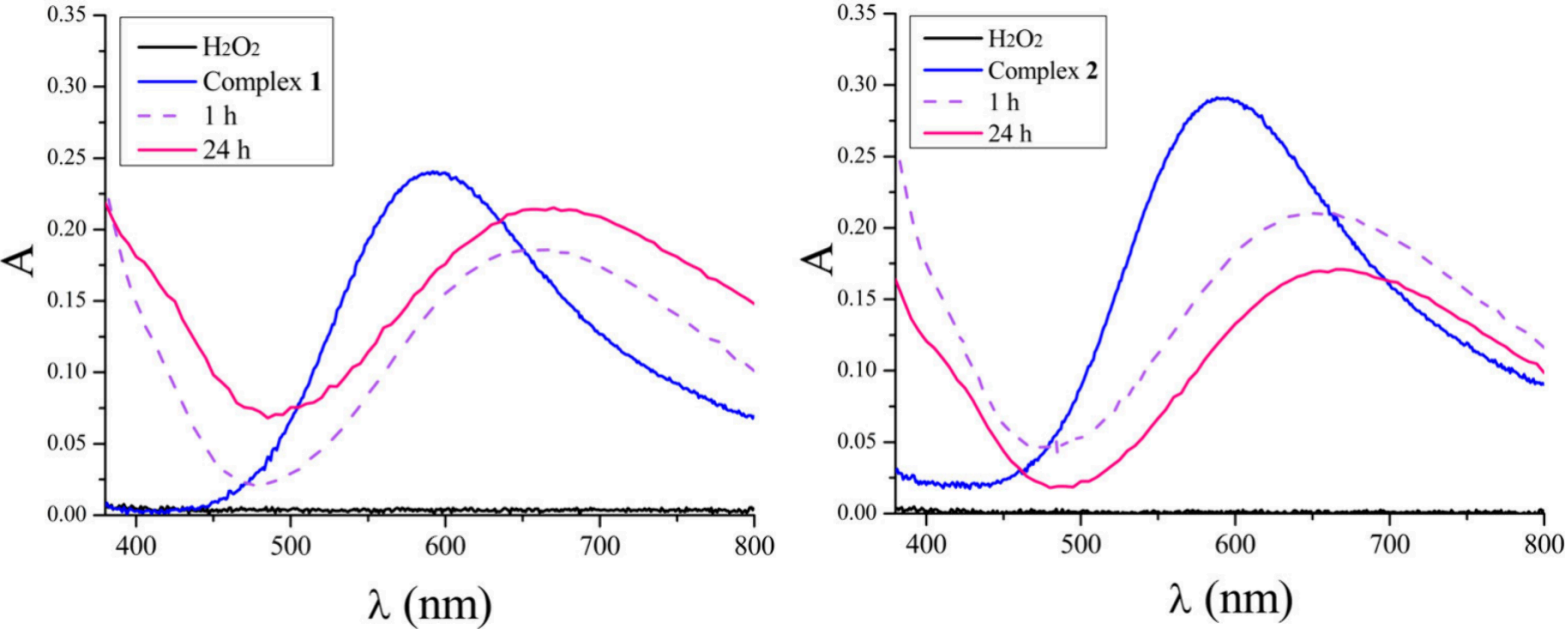
Interactions between complexes **1** (left) and **2** (right) and H_2_O_2_ monitored by UV–vis spectroscopy during 24 h of reaction time.

The interactions between the copper complexes and hydrogen peroxide are studied by using UV–vis and EPR spectroscopies. As H_2_O_2_ is EPR silent and does not exhibit any absorbance within the range of 380–800 nm of the UV–vis spectrum, the interactions were evaluated by monitoring changes related to the copper center by following the addition of H_2_O_2_ to a PBS solution of each complex. The left and right panels of Figure 11 display the typical d–d transitions of **1** and **2** (blue line), respectively. During 24 h, the λ_max_ values of complexes **1** and **2** are red-shifted from 600 to 655 nm, indicating that a similar species is formed after the reaction with H_2_O_2_.

**Figure 12:**
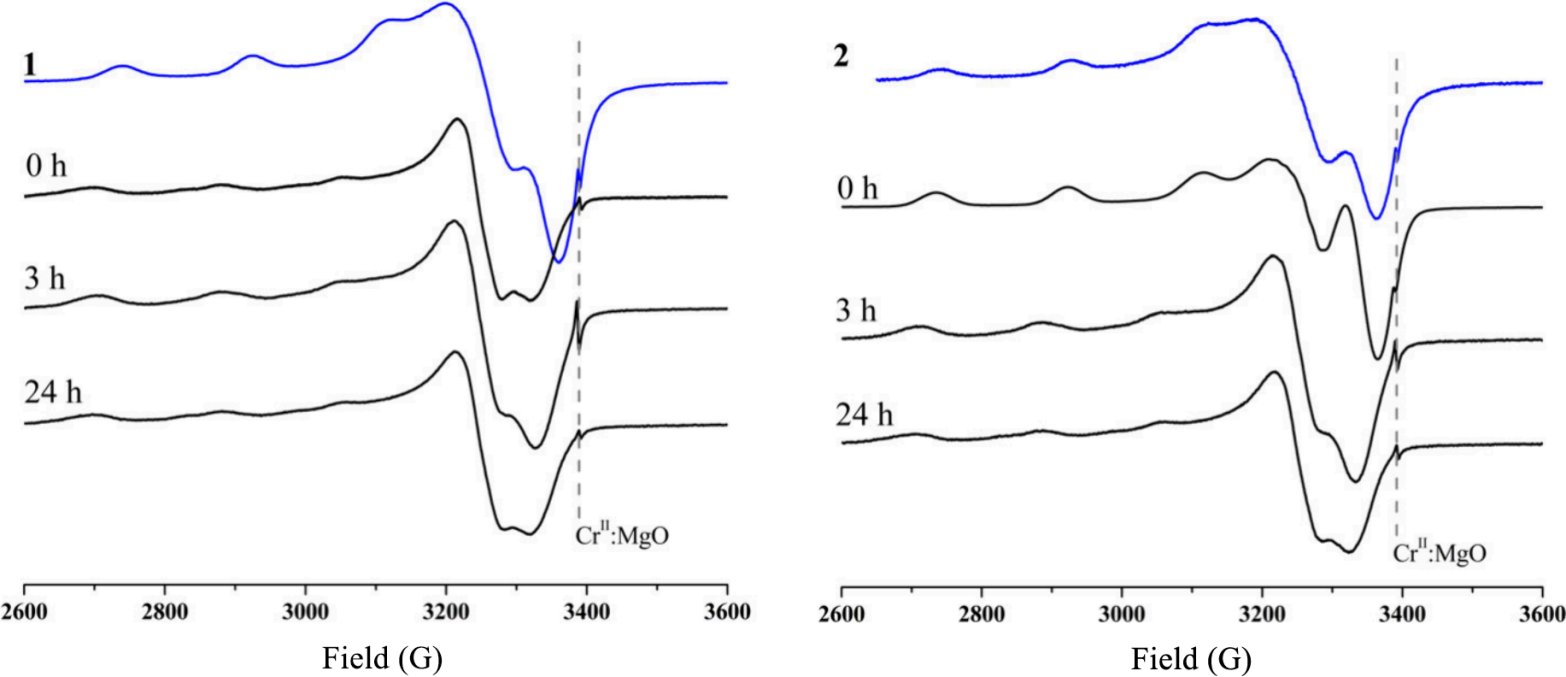
EPR spectra of complexes **1** (left) and **2** (right) before and after the interaction with H_2_O_2_ in a PBS solution (pH 7.4) at 120 K. The small signal closer to 3500 G is related to the standard MgO:Cr^3+^.

Similar to the UV–vis spectra, the EPR spectra of the complexes are comparable (Figure 12), showing a rhombic symmetry (*g_x_* = 2.067, *g_y_* = 2.098, *g_z_* = 2.25, *A_x_* = 10.078 G, *A_y_* = 8.86 G, and *A_z_* = 180.973 G for complex **1**, and *g_x_* = 2.102, *g_y_* = 2.069, *g_z_* = 2.244, *A_x_* = 0.867, *A_y_* = 0.701, and *A_z_* = 170.750 for complex **2**). After the reaction with H_2_O_2_, a broadening of the EPR lines is observed, with the following different *g* values: *g_x_* = 2.091, *g_y_* = 2.089, *g_z_* = 2.298, *A_x_* = 10.734, *A_y_* = 10.182, and *A_z_* = 171.954 G for complex **1**, and *g_x_* = 2.098, *g_y_* = 2.089, *g_z_* = 2.287, *A_x_* = 10.771, *A_y_* = 10.182, and *A_z_* = 170.889 G for complex **2**. They present a more axially distorted profile.

**Figure 13:**
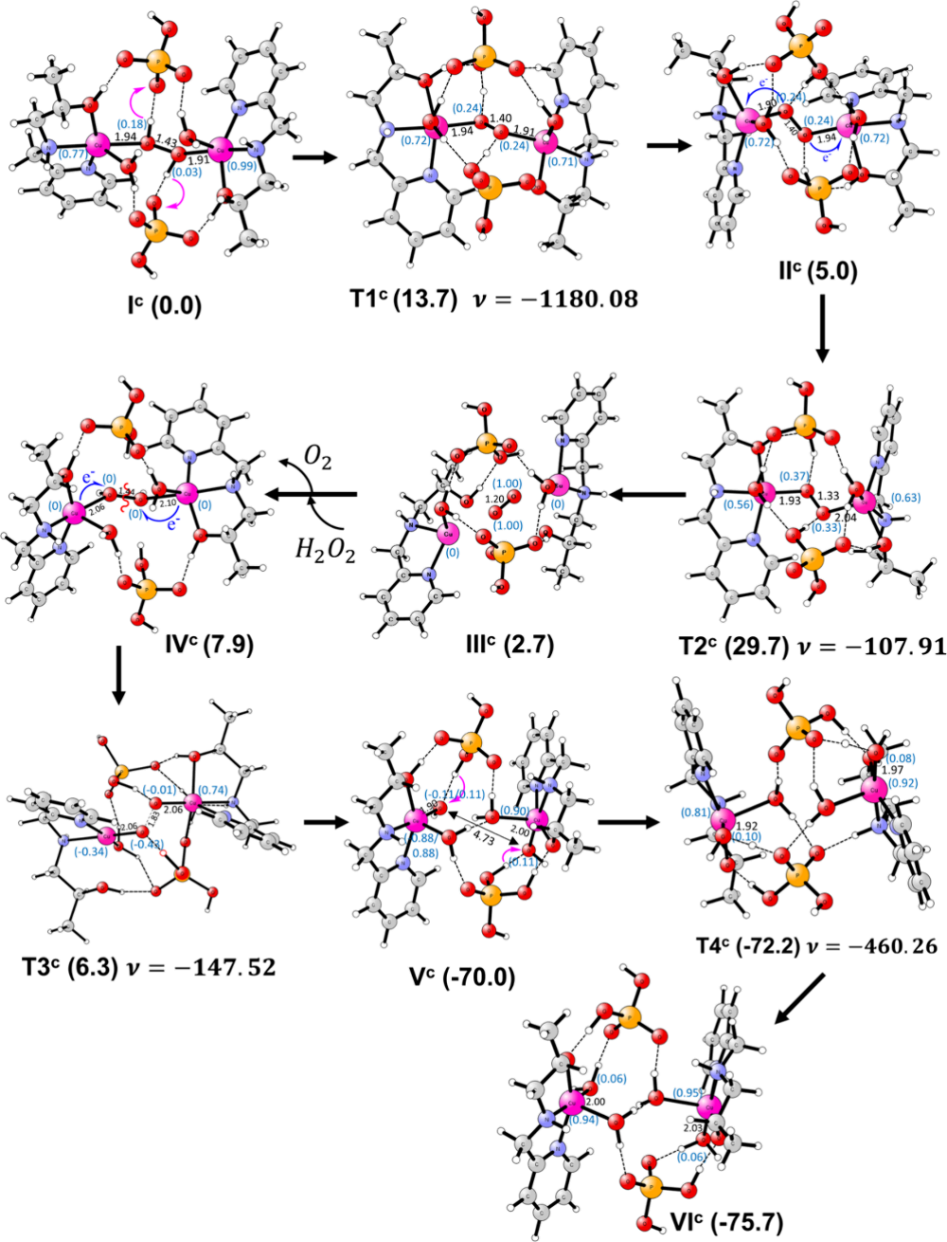
Structures of the CAT mechanism investigated using DFT calculations with key distances (in angstroms), electronic spins (blue), relative energies (in kilocalories per mole), and imaginary frequencies (in inverse centimeters).

The elementary steps presented in Scheme 2 are also investigated through DFT calculations. According to the proposed mechanism for the CAT activity (reaction b in Scheme 2), a H_2_O_2_ molecule forms a bridge between two [Cu(II)(HL)]^2+^ species. The four plausible optimized conformations for the reactant are shown in Figure S17b. They can be divided into *trans* and *cis* forms, i.e., three *trans* conformations (R^1^, R^2^, and R^3^) and one *cis* conformation (R^4^) in Figure S17b. The main differences among R^1^–R^3^ are the N^1^Cu^1^Cu^2^N^2^ dihederal angles of 105.22°, 130.68°, and −64.02°, respectively (Figure S17b). Among all four conformations, R^1^ (labeled as **I**^**C**^ in Figure 13) is the lowest in energy, and the remaining structures are higher in energy by 1.2–6.3 kcal/mol in the gas phase. Some Cu(II)-peroxo-Cu(II) complexes with the **I**^**C**^-like conformation have also been reported in the literature.^180−183^ In **I**^**C**^, the [(HL)Cu(II)-(H_2_O_2_)-Cu(II)(HL)]^4+^ species exists in the triplet state and is stabilized by a network of hydrogen bonds formed by two water and two phosphate molecules in a symmetric manner (Figure 13).^180−183^ From **I**^**C**^, the abstraction of both protons from H_2_O_2_ in a concerted fashion by phosphate occurs through a barrier of 13.7 kcal/mol (**T1**^**C**^). The [(HL)Cu(II)-(O-O)-Cu(II)(HL)]^2+^ complex (**II**^**C**^) created in this process is endothermic by 5.0 kcal/mol. From **II**^**C**^, two electrons from the bridging peroxide are synchronously transferred to both Cu(II) ions, with a barrier of 29.7 kcal/mol. In the transition state (**T2**^**C**^), the electron density of ∼0.60*e* on both Cu ions and 0.37*e* and 0.33*e* on the O^1^ and O^2^ atoms, respectively, indicate the concomitant transfer of both electrons. Due to the symmetric nature of both metal centers in **II**^**C**^, a stepwise electron transfer in this step is not feasible. This process leads to the formation of dioxygen and a binuclear [Cu(I)(HL)]_2_^2+^ complex stabilized by phosphate groups (**III**^**C**^). **III**^**C**^ is endothermic (2.7 kcal/mol) from **I**^**C**^. In the next step, dioxygen generated in the previous step is released, and another H_2_O_2_ molecule coordinates to **III**^**C**^ to form [(HL)Cu(I)-(H_2_O_2_)-Cu(I)(HL)]^2+^ (**IV**^**C**^). As a result, this species exists in the singlet state. The formation of **IV**^**C**^ is endothermic by 5.2 kcal/mol from **III**^**C**^, i.e., 7.9 kcal/mol endothermic from **I**^**C**^. From **IV**^**C**^, two electrons are concomitantly donated by both Cu(I) ions to H_2_O_2_. In the transition state (**T3**^**C**^) for this step, rather surprisingly, the transfer of electrons is nonequivalent. The electron densities of 0.34*e* and 0.74*e* on Cu^1^ and Cu^2^, respectively, suggest that the latter possesses more Cu(II) character than the former. The O^1^–O^2^ bond (1.83 Å) in this structure is broken. The cleavage of H_2_O_2_ takes place in a barrierless process, with **T3**^**C**^ being located 1.6 kcal/mol lower in energy than **IV**^**C**^. In the product (**V**^**C**^) of this step, both hydroxyl ions are coordinated to the Cu(II) ions. In **V**^**C**^, both open shell singlet and triplet states are degenerate, and it can switch to the triplet surface due to spin–orbit coupling. Due to the cleavage of the O–O bond of H_2_O_2_, the formation of **V**^**C**^ is highly exothermic by 70.0 kcal/mol. From **V**^**C**^, the donation of one proton each by two phosphate molecules to the hydroxyl ions creates water molecules (**VI**^**C**^) in a barrierless process through **T4**^**C**^. In **T4**^**C**^, both Cu^1^ and Cu^2^ ions are in the +2 oxidation state with spins of 0.81*e* and 0.92*e*, respectively. The formation of **VI**^**C**^ in the catalase activity is highly exothermic by 75.7 kcal/mol from **I**^**C**^.

#### Antioxidant Activity toward the Hydroxyl Radical

Due to the significant CAT and SOD-like activities of complexes **1** and **2** and the protection of C6 cells stressed with the hydroxyl radical shown by such compounds, their antioxidant activities against ^•^OH are also investigated using the EPR spin-trapping (DMPO) method.

Although the superoxide anion and hydrogen peroxide are formed under biological conditions, they are competently decomposed by natural metalloenzymes such as SOD and CAT.^184^ On the contrary, there is no enzymatic natural protection against one of the most dangerous ROS, the hydroxyl radical (^•^OH). The main reason for this may be its high reaction rate constant (10^9^ mol^–1^ L^–1^ s^–1^).^185^ Such species may be formed by different natural or synthetic processes (Fenton reaction, Haber–Weiss reaction, TiO_2_ photocatalytic process, ozone irradiation, hydrogen peroxide photolysis, etc.).^185^ From a biological point of view, the ^•^OH radical can readily oxidize DNA, lipids, and proteins.^72,186−188^ This process is directly associated with its high redox potential (2.8 V for ^•^OH/OH^–^).^189^ In living systems, the formation of ^•^OH species is related to the reaction of O_2_^•–^ or H_2_O_2_ with metal ions such as iron and copper,^190,191^ via Fenton or Haber–Weiss-like reactions.^192^

**Figure 14:**
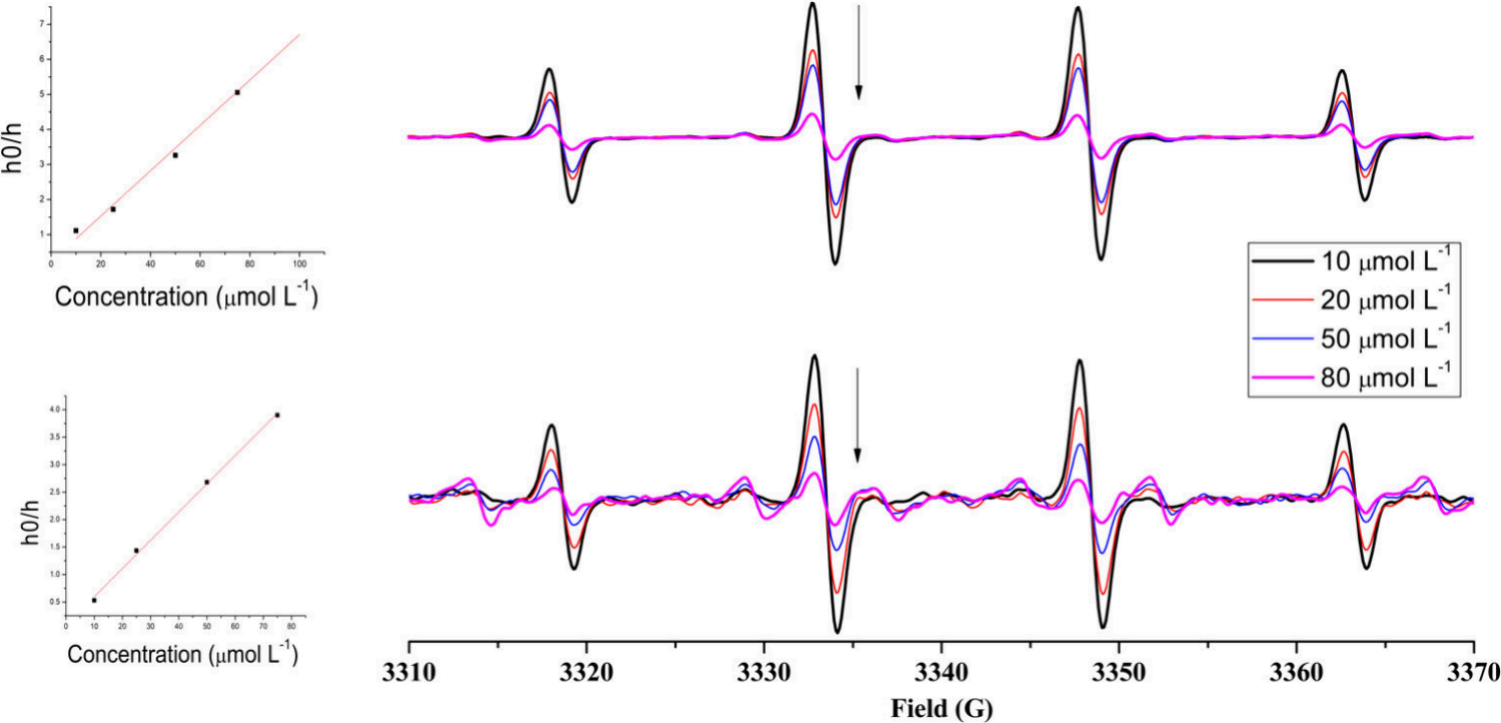
Effect of compounds **1** (top) and **2** (bottom) at different concentrations on the EPR signal intensity of the DMPO–OH adduct and plot (inset) of the signal intensity ratio in the absence and presence of the compounds employed in the calculation of RSA_50_.

As shown in Figure 14, upon addition of **1** and **2** at different concentrations, the intensity of the signal associated with adduct DMPO–OH (*a*_N_ = 15.0 G, and *a*_H_ = 14.7 G) decreases, indicating that the copper complexes react faster with the hydroxyl radical than DMPO. DMPO–OH signal depletion is linearly dependent on the copper concentration. Its plot (inset of Figure 14) shows RSA_50_ (radical scavenging activity 50%) values of 25.7 ± 0.5 and 37.2 ± 0.7 μmol L^–1^ for **1** and **2**, respectively.

There have been limited studies on the antioxidant activities of coordination complexes against ^•^OH, with most of the research focused on the antioxidant properties of organic molecules containing phenolic and polycyclic backbones and natural extracts.^193−197^ Although RSA_50_ can be dependent on the initial ^•^OH and DMPO concentration, the data employed here may be compared with those of compound [Cu(bmpa)Cl_2_] [bmpa = bis(pyridine-2-ylmethyl)amine] studied under the same conditions, which showed a RSA_50_ (1068 μmol L^–1^) much higher than those observed for compounds **1** and **2**.^137^

## Conclusions

This work describes the full characterization of two versatile copper complexes (**1** and **2**) synthesized with the bipodal tridentate ligand 1-(pyridin-2-ylmethylamino)propan-2-ol (HL). As observed by potentiometric titration and X-ray analysis, HL can form 1:1 {[Cu(HL)Cl_2_]} and 1:2 {[Cu(HL_2_)](ClO_4_)_2_} metal:ligand complexes. It is shown that this simple ligand exhibits antioxidant activity in protecting neuronal cells against metal (copper) and ROS (O_2_^–•^, H_2_O_2_, and ^•^OH) stress conditions. Furthermore, copper complexes also show antioxidant activities. The Cu(I)- and ROS-induced changes in the redox status of C6 cells are inhibited when the cells are treated with either the ligand HL, complex **1**, or complex **2**. The antioxidant activity of the ligand can be explained by its copper chelating capability, which usually induces damage via the Fenton reaction. The complexation of copper by HL results in the depletion of reactive metal inside the cells, avoiding the Fenton reaction. Thus, the cell’s redox status is kept at or closer to the basal level than in the untreated cells. Furthermore, it is shown that the complexation of HL to copper generates copper complexes that can catalytically decompose the superoxide anion and hydrogen peroxide. Therefore, the coordinated compounds formed are rare examples of copper complexes with dual antioxidant activities (SOD and CAT mimics). Additionally, the complexes also decompose hydroxyl radicals. Kinetic studies reveal that the SOD activity of complex **1** is better than that of **2** (IC_50_ values of 0.108 ± 0.065 and 0.326 ± 0.042, respectively). The same pattern is also observed in the investigation of CAT activity. The rate laws for CAT activities are *k*_obs_[**1**][H_2_O_2_], were *k*_obs_ = 0.061 ± 0.008 dm^3^ mol^–1^ s^–1^, and *k*_obs_[**2**]^1.3^[H_2_O_2_]^1.2^ for **1** and **2**, respectively. With regard to the antioxidant activity against the ^•^OH radical, the complexes show RSA_50_ values of 25.7 ± 0.5 and 37.2 ± 0.7 μmol L^–1^ for **1** and **2**, respectively. It is important to highlight that potentiometric titration data show that complex **2** is not stable at physiological pH and undergoes dissociation of one ligand molecule to generate **1**. UV–vis and EPR spectroscopies identified intermediate species such as Cu(II)-O_2_^•–^ and Cu(I)-O_2_^•–^ formed in the reactions of the compounds with O_2_^•–^_._ Together with theoretical calculations, a plausible mechanism is proposed by combining experimental and theoretical results. The DFT calculations suggest that the mechanism of superoxide oxidation and reduction is catalyzed by a mononuclear copper species. With respect to CAT activity, a reaction mechanism and the elementary reactions are proposed for **1** on the basis of the rate law. DFT calculations also supported the formation of a dinuclear-peroxide intermediate.

When compared with other molecules used in chelating therapies for copper depletion, it is noteworthy that the formed copper complexes (**1** and **2**) exhibit antioxidant activities. The discovery of a protective effect from a basic organic molecule (HL) against metal stress and its ability to form coordination compounds that exhibit inherent antioxidant activity against three distinct ROS are significant breakthroughs in the search for metallophores designed for treating diseases associated with copper accumulation and oxidative stress conditions. Therefore, the ligand HL emerges as a prototype of molecules whose activity can help in the development of new pharmaceuticals to treat diseases related to metal and ROS stress, including Alzheimer’s disease and Wilson’s disease.

## Supporting Information Available

UV–vis, ESI-(+)-MS, infrared, and EPR spectra of the compounds, experimental kinetic data related to SOD and CAT activities, initial conformations evaluated for the theoretical studies, crystallographic data, and steady-state treatment employed to determine the elementary steps involved in the CAT-like activity (PDF)

## References

[ref1] Sies H.; Jones D. P. Reactive Oxygen Species (ROS) as Pleiotropic Physiological Signalling Agents. Nat. Rev. Mol. Cell Biol. 2020, 21 (7), 363–383. 10.1038/s41580-020-0230-3. 32231263

[ref2] Andreyev A. Yu.; Kushnareva Yu. E.; Starkov A. A. Mitochondrial Metabolism of Reactive Oxygen Species. Biochemistry (Moscow) 2005, 70 (2), 200–214. 10.1007/s10541-005-0102-7. 15807660

[ref3] Lennicke C.; Cochemé H. M. Redox Metabolism: ROS as Specific Molecular Regulators of Cell Signaling and Function. Mol. Cell 2021, 81 (18), 3691–3707. 10.1016/j.molcel.2021.08.018. 34547234

[ref4] Matés J. M.; Segura J. A.; Alonso F. J.; Márquez J. Intracellular Redox Status and Oxidative Stress: Implications for Cell Proliferation, Apoptosis, and Carcinogenesis. Arch. Toxicol. 2008, 82 (5), 273–299. 10.1007/s00204-008-0304-z. 18443763

[ref5] Thannickal V. J.; Fanburg B. L. Reactive Oxygen Species in Cell Signaling. American Journal of Physiology-Lung Cellular and Molecular Physiology 2000, 279 (6), L1005–L1028. 10.1152/ajplung.2000.279.6.L1005. 11076791

[ref6] Schieber M.; Chandel N. S. ROS Function in Redox Signaling and Oxidative Stress. Curr. Biol. 2014, 24 (10), R453–R462. 10.1016/j.cub.2014.03.034. 24845678 PMC4055301

[ref7] Raimondi V.; Ciccarese F.; Ciminale V. Oncogenic Pathways and the Electron Transport Chain: A DangeROS Liaison. Br. J. Cancer 2020, 122 (2), 168–181. 10.1038/s41416-019-0651-y. 31819197 PMC7052168

[ref8] Nohl H.; Gille L.; Staniek K. Intracellular Generation of Reactive Oxygen Species by Mitochondria. Biochem. Pharmacol. 2005, 69 (5), 719–723. 10.1016/j.bcp.2004.12.002. 15710349

[ref9] Ursini F.; Maiorino M.; Forman H. J. Redox Homeostasis: The Golden Mean of Healthy Living. Redox Biol. 2016, 8, 205–215. 10.1016/j.redox.2016.01.010. 26820564 PMC4732014

[ref10] Ighodaro O. M.; Akinloye O. A. First Line Defence Antioxidants-Superoxide Dismutase (SOD), Catalase (CAT) and Glutathione Peroxidase (GPX): Their Fundamental Role in the Entire Antioxidant Defence Grid. Alexandria Journal of Medicine 2018, 54 (4), 287–293. 10.1016/j.ajme.2017.09.001.

[ref11] Sies H. Oxidative Stress: Oxidants and Antioxidants. Exp Physiol 1997, 82 (2), 291–295. 10.1113/expphysiol.1997.sp004024. 9129943

[ref12] Sies H. On the History of Oxidative Stress: Concept and Some Aspects of Current Development. Curr. Opin Toxicol 2018, 7, 122–126. 10.1016/j.cotox.2018.01.002.

[ref13] Sies H. Oxidative Stress. In Stress: Physiology, Biochemistry, and Pathology; Elsevier, 2019; pp 153–163. 10.1016/B978-0-12-813146-6.00013-8

[ref14] Sies H. Oxidative Stress: Concept and Some Practical Aspects. Antioxidants 2020, 9 (9), 852 10.3390/antiox9090852. 32927924 PMC7555448

[ref15] Sies H.; Berndt C.; Jones D. P. Oxidative Stress. Annu. Rev. Biochem. 2017, 86 (1), 715–748. 10.1146/annurev-biochem-061516-045037. 28441057

[ref16] Simpson D. S. A.; Oliver P. L. ROS Generation in Microglia: Understanding Oxidative Stress and Inflammation in Neurodegenerative Disease. Antioxidants 2020, 9 (8), 743 10.3390/antiox9080743. 32823544 PMC7463655

[ref17] Uddin Md. S.; Al Mamun A.; Kabir Md. T.; Ahmad J.; Jeandet P.; Sarwar Md. S.; Ashraf G. M.; Aleya L. Neuroprotective Role of Polyphenols against Oxidative Stress-Mediated Neurodegeneration. Eur. J. Pharmacol. 2020, 886, 173412 10.1016/j.ejphar.2020.173412. 32771668

[ref18] Cenini G.; Lloret A.; Cascella R. Oxidative Stress in Neurodegenerative Diseases: From a Mitochondrial Point of View. Oxid Med. Cell Longev 2019, 2019, 1–18. 10.1155/2019/2105607. PMC6532273 31210837

[ref19] Saikolappan S.; Kumar B.; Shishodia G.; Koul S.; Koul H. K. Reactive Oxygen Species and Cancer: A Complex Interaction. Cancer Lett. 2019, 452, 132–143. 10.1016/j.canlet.2019.03.020. 30905813

[ref20] Hayes J. D.; Dinkova-Kostova A. T.; Tew K. D. Oxidative Stress in Cancer. Cancer Cell 2020, 38 (2), 167–197. 10.1016/j.ccell.2020.06.001. 32649885 PMC7439808

[ref21] Arfin S.; Jha N. K.; Jha S. K.; Kesari K. K.; Ruokolainen J.; Roychoudhury S.; Rathi B.; Kumar D. Oxidative Stress in Cancer Cell Metabolism. Antioxidants 2021, 10 (5), 642 10.3390/antiox10050642. 33922139 PMC8143540

[ref22] Xu T.; Ding W.; Ji X.; Ao X.; Liu Y.; Yu W.; Wang J. Oxidative Stress in Cell Death and Cardiovascular Diseases. Oxid Med. Cell Longev 2019, 2019, 1–11. 10.1155/2019/9030563. PMC6875219 31781356

[ref23] Steven S.; Frenis K.; Oelze M.; Kalinovic S.; Kuntic M.; Bayo Jimenez M. T.; Vujacic-Mirski K.; Helmstädter J.; Kröller-Schön S.; Münzel T.; Daiber A. Vascular Inflammation and Oxidative Stress: Major Triggers for Cardiovascular Disease. Oxid Med. Cell Longev 2019, 2019, 1–26. 10.1155/2019/7092151. PMC6612399 31341533

[ref24] Ndrepepa G. Myeloperoxidase - A Bridge Linking Inflammation and Oxidative Stress with Cardiovascular Disease. Clin. Chim. Acta 2019, 493, 36–51. 10.1016/j.cca.2019.02.022. 30797769

[ref25] Michaeloudes C.; Abubakar-Waziri H.; Lakhdar R.; Raby K.; Dixey P.; Adcock I. M.; Mumby S.; Bhavsar P. K.; Chung K. F. Molecular Mechanisms of Oxidative Stress in Asthma. Mol. Aspects Med. 2022, 85, 101026 10.1016/j.mam.2021.101026. 34625291

[ref26] Dua K.; Malyla V.; Singhvi G.; Wadhwa R.; Krishna R. V.; Shukla S. D.; Shastri M. D.; Chellappan D. K.; Maurya P. K.; Satija S.; Mehta M.; Gulati M.; Hansbro N.; Collet T.; Awasthi R.; Gupta G.; Hsu A.; Hansbro P. M. Increasing Complexity and Interactions of Oxidative Stress in Chronic Respiratory Diseases: An Emerging Need for Novel Drug Delivery Systems. Chem. Biol. Interact 2019, 299, 168–178. 10.1016/j.cbi.2018.12.009. 30553721

[ref27] Butterfield D. A. Brain Lipid Peroxidation and Alzheimer Disease: Synergy between the Butterfield and Mattson Laboratories. Ageing Res. Rev. 2020, 64, 101049 10.1016/j.arr.2020.101049. 32205035 PMC7502429

[ref28] Tsaluchidu S.; Cocchi M.; Tonello L.; Puri B. K. Fatty Acids and Oxidative Stress in Psychiatric Disorders. BMC Psychiatry 2008, 8 (S1), S5 10.1186/1471-244X-8-S1-S5. 18433515 PMC2330073

[ref29] Greenough M. A.; Camakaris J.; Bush A. I. Metal Dyshomeostasis and Oxidative Stress in Alzheimer’s Disease. Neurochem. Int. 2013, 62 (5), 540–555. 10.1016/j.neuint.2012.08.014. 22982299

[ref30] Smith D. G.; Cappai R.; Barnham K. J. The Redox Chemistry of the Alzheimer’s Disease Amyloid β Peptide. Biochimica et Biophysica Acta (BBA) - Biomembranes 2007, 1768 (8), 1976–1990. 10.1016/j.bbamem.2007.02.002. 17433250

[ref31] Winterbourn C. C. Toxicity of Iron and Hydrogen Peroxide: The Fenton Reaction. Toxicol. Lett. 1995, 82–83, 969–974. 10.1016/0378-4274(95)03532-X. 8597169

[ref32] Biliński T.; Krawiec Z.; Liczmański A.; Litwińska J. Is Hydroxyl Radical Generated by the Fenton Reaction in Vivo?. Biochem. Biophys. Res. Commun. 1985, 130 (2), 533–539. 10.1016/0006-291X(85)90449-8. 2992473

[ref33] Lloyd R. V.; Hanna P. M.; Mason R. P. The Origin of the Hydroxyl Radical Oxygen in the Fenton Reaction. Free Radic Biol. Med. 1997, 22 (5), 885–888. 10.1016/S0891-5849(96)00432-7. 9119257

[ref34] Li J.; Cao F.; Yin H.; Huang Z.; Lin Z.; Mao N.; Sun B.; Wang G. Ferroptosis: Past, Present and Future. Cell Death Dis 2020, 11 (2), 88 10.1038/s41419-020-2298-2. 32015325 PMC6997353

[ref35] Halliwell B.; Adhikary A.; Dingfelder M.; Dizdaroglu M. Hydroxyl Radical Is a Significant Player in Oxidative DNA Damage *in Vivo*. Chem. Soc. Rev. 2021, 50 (15), 8355–8360. 10.1039/D1CS00044F. 34128512 PMC8328964

[ref36] Du J.; Gebicki J. M. Proteins Are Major Initial Cell Targets of Hydroxyl Free Radicals. Int. J. Biochem. Cell Biol. 2004, 36 (11), 2334–2343. 10.1016/j.biocel.2004.05.012. 15313477

[ref37] Hosseinpour Mashkani S. M.; Bishop D. P.; Raoufi-Rad N.; Adlard P. A.; Shimoni O.; Golzan S. M. Distribution of Copper, Iron, and Zinc in the Retina, Hippocampus, and Cortex of the Transgenic APP/PS1Mouse Model of Alzheimer’s Disease. Cells 2023, 12 (8), 1144 10.3390/cells12081144. 37190053 PMC10136451

[ref38] Huat T. J.; Camats-Perna J.; Newcombe E. A.; Valmas N.; Kitazawa M.; Medeiros R. Metal Toxicity Links to Alzheimer’s Disease and Neuroinflammation. J. Mol. Biol. 2019, 431 (9), 1843–1868. 10.1016/j.jmb.2019.01.018. 30664867 PMC6475603

[ref39] Bush A. Metals and Neuroscience. Curr. Opin Chem. Biol. 2000, 4 (2), 184–191. 10.1016/S1367-5931(99)00073-3. 10742195

[ref40] Chelikani P.; Fita I.; Loewen P. C. Diversity of Structures and Properties among Catalases. Cell. Mol. Life Sci. 2004, 61 (2), 192–208. 10.1007/s00018-003-3206-5. 14745498 PMC11138816

[ref41] Sandalio L. M.; Rodríguez-Serrano M.; Romero-Puertas M. C.; del Río L. A. Role of Peroxisomes as a Source of Reactive Oxygen Species (ROS) Signaling Molecules. Subcell. Biochem. 2013, 69, 231–255. 10.1007/978-94-007-6889-5_13. 23821152

[ref42] McNew J. A.; Goodman J. M. An Oligomeric Protein Is Imported into Peroxisomes in Vivo. J. Cell Biol. 1994, 127 (5), 1245–1257. 10.1083/jcb.127.5.1245. 7962087 PMC2120261

[ref43] Krishnamurthy P.; Wadhwani A. Antioxidant Enzymes and Human Health. In Antioxidant Enzyme; InTech, 2012. 10.5772/48109

[ref44] Banjarnahor S. D. S.; Artanti N. Antioxidant Properties of Flavonoids. Medical Journal of Indonesia 2015, 23 (4), 239–244. 10.13181/mji.v23i4.1015.

[ref45] Wang X.; Li Y.; Han L.; Li J.; Liu C.; Sun C. Role of Flavonoids in the Treatment of Iron Overload. Front. Cell Dev. Biol. 2021, 9, 685364 10.3389/fcell.2021.685364. 34291050 PMC8287860

[ref46] Tapas A.; Sakarkar D.; Kakde R. Flavonoids as Nutraceuticals: A Review. Tropical Journal of Pharmaceutical Research 2008, 7 (3), 1089 10.4314/tjpr.v7i3.14693.

[ref47] Panche A. N.; Diwan A. D.; Chandra S. R. Flavonoids: An Overview. J. Nutr Sci. 2016, 5, e47 10.1017/jns.2016.41. 28620474 PMC5465813

[ref48] Yang W.; Yang X.; Zhu L.; Chu H.; Li X.; Xu W. Nanozymes: Activity Origin, Catalytic Mechanism, and Biological Application. Coord. Chem. Rev. 2021, 448, 214170 10.1016/j.ccr.2021.214170.

[ref49] Signorella S.; Hureau C. Bioinspired Functional Mimics of the Manganese Catalases. Coord. Chem. Rev. 2012, 256 (11–12), 1229–1245. 10.1016/j.ccr.2012.02.003.

[ref50] Signorella S.; Palopoli C.; Ledesma G. Rationally Designed Mimics of Antioxidant Manganoenzymes: Role of Structural Features in the Quest for Catalysts with Catalase and Superoxide Dismutase Activity. Coord. Chem. Rev. 2018, 365, 75–102. 10.1016/j.ccr.2018.03.005.

[ref51] Deawati Y.; Onggo D.; Mulyani I.; Hastiawan I.; Kurnia D.; Lönnecke P.; Schmorl S.; Kersting B.; Hey-Hawkins E. Synthesis, Crystal Structures, and Superoxide Dismutase Activity of Two New Multinuclear Manganese(III)-Salen-4,4′-Bipyridine Complexes. Inorg. Chim. Acta 2018, 482, 353–357. 10.1016/j.ica.2018.06.018.

[ref52] Deawati Y.; Onggo D.; Mulyani I.; Hastiawan I.; Utami R. A.; Kurnia D. Synthesis of [Mn(Salen)Cl] Complex Compound and Superoxide Dismutase Activity Determination through Non-Enzymatic Method. Key Eng. Mater. 2019, 811, 22–27. 10.4028/www.scientific.net/KEM.811.22.

[ref53] Kupershmidt L.; Okun Z.; Amit T.; Mandel S.; Saltsman I.; Mahammed A.; Bar-Am O.; Gross Z.; Youdim M. B. H. Metallocorroles as Cytoprotective Agents against Oxidative and Nitrative Stress in Cellular Models of Neurodegeneration. J. Neurochem 2010, 113 (2), 363–373. 10.1111/j.1471-4159.2010.06619.x. 20096090

[ref54] Eckshtain M.; Zilbermann I.; Mahammed A.; Saltsman I.; Okun Z.; Maimon E.; Cohen H.; Meyerstein D.; Gross Z. Superoxide Dismutase Activity of Corrole Metal Complexes. Dalton Transactions 2009, (38), 7879 10.1039/b911278b. 19771348

[ref55] Doctrow S. R.; Huffman K.; Marcus C. B.; Tocco G.; Malfroy E.; Adinolfi C. A.; Kruk H.; Baker K.; Lazarowych N.; Mascarenhas J.; Malfroy B. Salen-Manganese Complexes as Catalytic Scavengers of Hydrogen Peroxide and Cytoprotective Agents: Structure-Activity Relationship Studies. J. Med. Chem. 2002, 45 (20), 4549–4558. 10.1021/jm020207y. 12238934

[ref56] Batinić-Haberle I.; Rebouças J. S.; Spasojević I. Superoxide Dismutase Mimics: Chemistry, Pharmacology, and Therapeutic Potential. Antioxid Redox Signal 2010, 13 (6), 877–918. 10.1089/ars.2009.2876. 20095865 PMC2935339

[ref57] Day B. J. Catalase and Glutathione Peroxidase Mimics. Biochem. Pharmacol. 2009, 77 (3), 285–296. 10.1016/j.bcp.2008.09.029. 18948086 PMC2657365

[ref58] Baudry M.; Etienne S.; Bruce A.; Palucki M.; Jacobsen E.; Malfroy B. Salen-Manganese Complexes Are Superoxide Dismutase-Mimics. Biochem. Biophys. Res. Commun. 1993, 192 (2), 964–968. 10.1006/bbrc.1993.1509. 8484797

[ref59] Friedel F. C.; Lieb D.; Ivanović-Burmazović I. Comparative Studies on Manganese-Based SOD Mimetics, Including the Phosphate Effect, by Using Global Spectral Analysis. J. Inorg. Biochem 2012, 109, 26–32. 10.1016/j.jinorgbio.2011.12.008. 22366231

[ref60] Batinic-Haberle I.; Rajic Z.; Tovmasyan A.; Reboucas J. S.; Ye X.; Leong K. W.; Dewhirst M. W.; Vujaskovic Z.; Benov L.; Spasojevic I. Diverse Functions of Cationic Mn(III) N-Substituted Pyridylporphyrins, Recognized as SOD Mimics. Free Radic Biol. Med. 2011, 51 (5), 1035–1053. 10.1016/j.freeradbiomed.2011.04.046. 21616142 PMC3178885

[ref61] Ribeiro T. P.; Fernandes C.; Melo K. V.; Ferreira S. S.; Lessa J. A.; Franco R. W. A.; Schenk G.; Pereira M. D.; Horn A. Iron, Copper, and Manganese Complexes with in Vitro Superoxide Dismutase and/or Catalase Activities That Keep Saccharomyces Cerevisiae Cells Alive under Severe Oxidative Stress. Free Radic Biol. Med. 2015, 80, 67–76. 10.1016/j.freeradbiomed.2014.12.005. 25511255

[ref62] Policar C.; Bouvet J.; Bertrand H. C.; Delsuc N. SOD Mimics: From the Tool Box of the Chemists to Cellular Studies. Curr. Opin Chem. Biol. 2022, 67, 102109 10.1016/j.cbpa.2021.102109. 35066373

[ref63] Spiers J. G.; Cortina Chen H.-J.; Barry T. L.; Bourgognon J.-M.; Steinert J. R. Redox Stress and Metal Dys-Homeostasis Appear as Hallmarks of Early Prion Disease Pathogenesis in Mice. Free Radic Biol. Med. 2022, 192, 182–190. 10.1016/j.freeradbiomed.2022.09.025. 36170956

[ref64] Gromadzka G.; Tarnacka B.; Flaga A.; Adamczyk A. Copper Dyshomeostasis in Neurodegenerative Diseases—Therapeutic Implications. Int. J. Mol. Sci. 2020, 21 (23), 9259 10.3390/ijms21239259. 33291628 PMC7730516

[ref65] Greenough M. A.; Camakaris J.; Bush A. I. Metal Dyshomeostasis and Oxidative Stress in Alzheimer’s Disease. Neurochem. Int. 2013, 62 (5), 540–555. 10.1016/j.neuint.2012.08.014. 22982299

[ref66] Alfano M.; Cavazza C. Structure, Function, and Biosynthesis of Nickel-dependent Enzymes. Protein Sci. 2020, 29 (5), 1071–1089. 10.1002/pro.3836. 32022353 PMC7184782

[ref67] Ghosh A. C.; Duboc C.; Gennari M. Synergy between Metals for Small Molecule Activation: Enzymes and Bio-Inspired Complexes. Coord. Chem. Rev. 2021, 428, 213606 10.1016/j.ccr.2020.213606.

[ref68] Nuran Ercal B. S. P.; Hande Gurer-Orhan B. S. P.; Nukhet Aykin-Burns B. S. P. Toxic Metals and Oxidative Stress Part I: Mechanisms Involved in Me-Tal Induced Oxidative Damage. Curr. Top Med. Chem. 2001, 1 (6), 529–539. 10.2174/1568026013394831. 11895129

[ref69] Leonard S. S.; Harris G. K.; Shi X. Metal-Induced Oxidative Stress and Signal Transduction. Free Radic Biol. Med. 2004, 37 (12), 1921–1942. 10.1016/j.freeradbiomed.2004.09.010. 15544913

[ref70] Jomova K.; Valko M. Advances in Metal-Induced Oxidative Stress and Human Disease. Toxicology 2011, 283 (2–3), 65–87. 10.1016/j.tox.2011.03.001. 21414382

[ref71] Li J.; Cao F.; Yin H.; Huang Z.; Lin Z.; Mao N.; Sun B.; Wang G. Ferroptosis: Past, Present and Future. Cell Death Dis 2020, 11 (2), 88 10.1038/s41419-020-2298-2. 32015325 PMC6997353

[ref72] Halliwell B.; Adhikary A.; Dingfelder M.; Dizdaroglu M. Hydroxyl Radical Is a Significant Player in Oxidative DNA Damage *in Vivo*. Chem. Soc. Rev. 2021, 50 (15), 8355–8360. 10.1039/D1CS00044F. 34128512 PMC8328964

[ref73] Du J.; Gebicki J. M. Proteins Are Major Initial Cell Targets of Hydroxyl Free Radicals. Int. J. Biochem. Cell Biol. 2004, 36 (11), 2334–2343. 10.1016/j.biocel.2004.05.012. 15313477

[ref74] Fasae K. D.; Abolaji A. O.; Faloye T. R.; Odunsi A. Y.; Oyetayo B. O.; Enya J. I.; Rotimi J. A.; Akinyemi R. O.; Whitworth A. J.; Aschner M. Metallobiology and Therapeutic Chelation of Biometals (Copper, Zinc and Iron) in Alzheimer’s Disease: Limitations, and Current and Future Perspectives. Journal of Trace Elements in Medicine and Biology 2021, 67, 126779 10.1016/j.jtemb.2021.126779. 34034029

[ref75] Andersen O. Principles and Recent Developments in Chelation Treatment of Metal Intoxication. Chem. Rev. 1999, 99 (9), 2683–2710. 10.1021/cr980453a. 11749497

[ref76] Hegde M. L.; Bharathi P.; Suram A.; Venugopal C.; Jagannathan R.; Poddar P.; Srinivas P.; Sambamurti K.; Rao K. J.; Scancar J.; Messori L.; Zecca L.; Zatta P. Challenges Associated with Metal Chelation Therapy in Alzheimer’s Disease. J. Alzheimer's Dis. 2009, 17 (3), 457–468. 10.3233/JAD-2009-1068. 19363258 PMC2931820

[ref77] Budimir A. Metal Ions, Alzheimer’s Disease and Chelation Therapy. Acta Pharmaceutica 2011, 61 (1), 1–14. 10.2478/v10007-011-0006-6. 21406339

[ref78] Chaudhari V.; Bagwe-Parab S.; Buttar H. S.; Gupta S.; Vora A.; Kaur G. Challenges and Opportunities of Metal Chelation Therapy in Trace Metals Overload-Induced Alzheimer’s Disease. Neurotox Res. 2023, 41 (3), 270–287. 10.1007/s12640-023-00634-7. 36705861

[ref79] Esmieu C.; Guettas D.; Conte-Daban A.; Sabater L.; Faller P.; Hureau C. Copper-Targeting Approaches in Alzheimer’s Disease: How To Improve the Fallouts Obtained from in Vitro Studies. Inorg. Chem. 2019, 58 (20), 13509–13527. 10.1021/acs.inorgchem.9b00995. 31247877

[ref80] Savelieff M. G.; Nam G.; Kang J.; Lee H. J.; Lee M.; Lim M. H. Development of Multifunctional Molecules as Potential Therapeutic Candidates for Alzheimer’s Disease, Parkinson’s Disease, and Amyotrophic Lateral Sclerosis in the Last Decade. Chem. Rev. 2019, 119 (2), 1221–1322. 10.1021/acs.chemrev.8b00138. 30095897

[ref81] Perez D. R.; Sklar L. A.; Chigaev A. Clioquinol: To Harm or Heal. Pharmacol Ther 2019, 199, 155–163. 10.1016/j.pharmthera.2019.03.009. 30898518 PMC6571072

[ref82] Finkelstein D. I.; Hare D. J.; Billings J. L.; Sedjahtera A.; Nurjono M.; Arthofer E.; George S.; Culvenor J. G.; Bush A. I.; Adlard P. A. Clioquinol Improves Cognitive, Motor Function, and Microanatomy of the Alpha-Synuclein HA53T Transgenic Mice. ACS Chem. Neurosci. 2016, 7 (1), 119–129. 10.1021/acschemneuro.5b00253. 26481462

[ref83] Carvalho A.; Barbosa B. M.; Flores J. S.; do Carmo Gonçalves P.; Diniz R.; Cordeiro Y.; Fernández C. O.; Cukierman D. S.; Rey N. A. New Mescaline-Related N-Acylhydrazone and Its Unsubstituted Benzoyl Derivative: Promising Metallophores for Copper-Associated Deleterious Effects Relief in Alzheimer’s Disease. J. Inorg. Biochem 2023, 238, 112033 10.1016/j.jinorgbio.2022.112033. 36396525

[ref84] Gomes L. M. F.; Vieira R. P.; Jones M. R.; Wang M. C. P.; Dyrager C.; Souza-Fagundes E. M.; Da Silva J. G.; Storr T.; Beraldo H. 8-Hydroxyquinoline Schiff-Base Compounds as Antioxidants and Modulators of Copper-Mediated Aβ Peptide Aggregation. J. Inorg. Biochem 2014, 139, 106–116. 10.1016/j.jinorgbio.2014.04.011. 25019963

[ref85] O'Reilly J. E. Oxidation-Reduction Potential of the Ferro-Ferricyanide System in Buffer Solutions. Biochim. Biophys. Acta 1973, 292, 509–515. 10.1016/0005-2728(73)90001-7. 4705442

[ref86] Arthur E.; Martell R. J. Motekaitis. Determination and Use of Stability Constants, 2nd ed.; VCH Publishers: New York, 1992.

[ref87] Baes C. F.; Messmer R. E. Hydrolysis of Cations; 1976.

[ref88] Sheldrick G. M. A Short History of *SHELX*. Acta Crystallogr. A 2008, 64 (1), 112–122. 10.1107/S0108767307043930. 18156677

[ref89] Sheldrick G. M. Crystal Structure Refinement with *SHELXL*. Acta Crystallogr. C Struct Chem. 2015, 71 (1), 3–8. 10.1107/S2053229614024218. 25567568 PMC4294323

[ref90] DIAMOND-Crystal and Molecular Structure Visualization, ver. 4; Crystal Impact, 2006.

[ref91] Striegler S.; Dittel M. A Sugar’s Choice: Coordination to a Mononuclear or a Dinuclear Copper(II) Complex?. Inorg. Chem. 2005, 44 (8), 2728–2733. 10.1021/ic048724p. 15819559

[ref92] GUIEU S.; LANFREDI A.; MASSERA C.; PACHON L.; GAMEZ P.; REEDIJK J. New,-Containing Ligands for the Biomimetic Copper-Catalyzed Polymerization of 2,6-Dimethylphenol. Catal. Today 2004, 96 (4), 259–264. 10.1016/j.cattod.2004.06.149.

[ref93] Swarnkar S.; Singh S.; Goswami P.; Mathur R.; Patro I. K.; Nath C. Astrocyte Activation: A Key Step in Rotenone Induced Cytotoxicity and DNA Damage. Neurochem. Res. 2012, 37 (10), 2178–2189. 10.1007/s11064-012-0841-y. 22846965

[ref94] de Menezes M. N.; Kogawa A. C.; Salgado H. R. N. Status of Hydrogen Peroxide Solution 10 V in Commercialized Samples. Pharm. Anal. Acta 2017, 8, 10 10.4172/2153-2435.1000567.

[ref95] Novotná R.; Trávníček Z.; Herchel R. SOD-Mimic Cu(II) Dimeric Complexes Involving Kinetin and Its Derivative: Preparation and Characterization. Bioinorg Chem. Appl. 2012, 2012, 1–8. 10.1155/2012/704329. PMC3433123 22966218

[ref96] Ledesma G. N.; Eury H.; Anxolabéhère-Mallart E.; Hureau C.; Signorella S. R. A New Mononuclear Manganese(III) Complex of an Unsymmetrical Hexadentate N3O3 Ligand Exhibiting Superoxide Dismutase and Catalase-like Activity: Synthesis, Characterization, Properties and Kinetics Studies. J. Inorg. Biochem 2015, 146, 69–76. 10.1016/j.jinorgbio.2015.02.012. 25771435

[ref97] Grau M.; Rigodanza F.; White A. J. P.; Sorarù A.; Carraro M.; Bonchio M.; Britovsek G. J. P. Ligand Tuning of Single-Site Manganese-Based Catalytic Antioxidants with Dual Superoxide Dismutase and Catalase Activity. Chem. Commun. 2014, 50 (35), 4607–4609. 10.1039/C4CC00758A. 24667888

[ref98] Valentine J. S.; Miksztal A. R.; Sawyer D. T. [7] Methods for the Study of Superoxide Chemistry in Nonaqueous Solutions. Oxygen Radicals in Biological Systems 1984, 105, 71–81. 10.1016/S0076-6879(84)05010-2. 6328207

[ref99] Seyb E.; Kleinberg J. Determination of Superoxide Oxygen. Anal. Chem. 1951, 23 (1), 115–117. 10.1021/ac60049a021.

[ref100] Burgos Castillo R. C.; Fontmorin J.-M.; Tang W. Z.; Dominguez-Benetton X.; Sillanpää M. Towards Reliable Quantification of Hydroxyl Radicals in the Fenton Reaction Using Chemical Probes. RSC Adv. 2018, 8 (10), 5321–5330. 10.1039/C7RA13209C. 35542446 PMC9078104

[ref101] Frisch M. J.; Trucks G. W.; Schlegel H. B.; Scuseria G. E.; Robb M. A.; Cheeseman J. R.; Scalmani G.; Barone V.; Petersson G. A.; Nakatsuji H.; Li X.; Caricato M.; Marenich A. V.; Bloino J.; Janesko B. G.; Gomperts R.; Mennucci B.; Hratchian H. P.; Ortiz J. V.; Izmaylov A. F.; Sonnenberg J. L.; Williams-Young D.; Ding F.; Lipparini F.; Egidi F.; Goings J.; Peng B.; Petrone A.; Henderson T.; Ranasinghe D.; Zakrzewski V. G.; Gao J.; Rega N.; Zheng G.; Liang W.; Hada M.; Ehara M.; Toyota K.; Fukuda R.; Hasegawa J.; Ishida M.; Nakajima T.; Honda Y.; Kitao O.; Nakai H.; Vreven T.; Throssell K.; Montgomery J. A. Jr.; Peralta J. E.; Ogliaro F.; Bearpark M. J.; Heyd J. J.; Brothers E. N.; Kudin K. N.; Staroverov V. N.; Keith T. A.; Kobayashi R.; Normand J.; Raghavachari K.; Rendell A. P.; Burant J. C.; Iyengar S. S.; Tomasi J.; Cossi M.; Millam J. M.; Klene M.; Adamo C.; Cammi R.; Ochterski J. W.; Martin R. L.; Morokuma K.; Farkas O.; Foresman J. B.; Fox D. J. Gaussian 16, rev. C.01; Gaussian, Inc.: Wallingford, CT, 2016.

[ref102] Adamo C.; Barone V. Exchange Functionals with Improved Long-Range Behavior and Adiabatic Connection Methods without Adjustable Parameters: The MPW and MPW1PW Models. J. Chem. Phys. 1998, 108 (2), 664–675. 10.1063/1.475428.

[ref103] Hay P. J.; Wadt W. R. *Ab Initio* Effective Core Potentials for Molecular Calculations. Potentials for the Transition Metal Atoms Sc to Hg. J. Chem. Phys. 1985, 82 (1), 270–283. 10.1063/1.448799.

[ref104] Krishnan R.; Binkley J. S.; Seeger R.; Pople J. A. Self-Consistent Molecular Orbital Methods. XX. A Basis Set for Correlated Wave Functions. J. Chem. Phys. 1980, 72 (1), 650–654. 10.1063/1.438955.

[ref105] Clark T.; Chandrasekhar J.; Spitznagel G. W.; Schleyer P. V. R. Efficient Diffuse Function-augmented Basis Sets for Anion Calculations. III. The 3-21+G Basis Set for First-row Elements, Li-F. J. Comput. Chem. 1983, 4 (3), 294–301. 10.1002/jcc.540040303.

[ref106] Roy L. E.; Hay P. J.; Martin R. L. Revised Basis Sets for the LANL Effective Core Potentials. J. Chem. Theory Comput 2008, 4 (7), 1029–1031. 10.1021/ct8000409. 26636355

[ref107] Cancès E.; Mennucci B.; Tomasi J. A New Integral Equation Formalism for the Polarizable Continuum Model: Theoretical Background and Applications to Isotropic and Anisotropic Dielectrics. J. Chem. Phys. 1997, 107 (8), 3032–3041. 10.1063/1.474659.

[ref108] Tomasi J.; Mennucci B.; Cammi R. Quantum Mechanical Continuum Solvation Models. Chem. Rev. 2005, 105 (8), 2999–3094. 10.1021/cr9904009. 16092826

[ref109] Grimme S.; Ehrlich S.; Goerigk L. Effect of the Damping Function in Dispersion Corrected Density Functional Theory. J. Comput. Chem. 2011, 32 (7), 1456–1465. 10.1002/jcc.21759. 21370243

[ref110] Grimme S.; Antony J.; Ehrlich S.; Krieg H. A Consistent and Accurate *Ab Initio* Parametrization of Density Functional Dispersion Correction (DFT-D) for the 94 Elements H-Pu. J. Chem. Phys. 2010, 132 (15), 154104 10.1063/1.3382344. 20423165

[ref111] Nadarkhani S.; Golchoubian H.; Shirvan A. Synthesis, Crystal Structure, and Chromotropism Properties of Dihalo N-2-Ethanolpicolylamine Copper(II). J. Mol. Struct. 2023, 1276, 134801 10.1016/j.molstruc.2022.134801.

[ref112] Fernandes C.; Horn A.; Vieira-da-Motta O.; Kanashiro M. M.; Rocha M. R.; Moreira R. O.; Morcelli S. R.; Lopes B. F.; Mathias L. da S.; Borges F. V.; Borges L. J. H.; Freitas W. R.; Visentin L. C.; Almeida J. C. de A.; Schenk G. Synthesis, Characterization, Antibacterial and Antitumoral Activities of Mononuclear Zinc Complexes Containing Tridentate Amine Based Ligands with N3 or N2O Donor Groups. Inorg. Chim. Acta 2014, 416, 35–48. 10.1016/j.ica.2014.02.040.

[ref113] Borges L. J. H.; Bull É. S.; Fernandes C.; Horn A.; Azeredo N. F.; Resende J. A. L. C.; Freitas W. R.; Carvalho E. C. Q.; Lemos L. S.; Jerdy H.; Kanashiro M. M. In Vitro and in Vivo Studies of the Antineoplastic Activity of Copper (II) Compounds against Human Leukemia THP-1 and Murine Melanoma B16-F10 Cell Lines. Eur. J. Med. Chem. 2016, 123, 128–140. 10.1016/j.ejmech.2016.07.018. 27474929

[ref114] Azeredo N. F. B.; Bull E. S.; Resende J. A. L. C.; Horn A.; Fernandes C. Crystal Structure and Behavior in Solution of [Cu(HBPA)2]Cl2·4H2O [HBPA = (2-Hydroxybenzyl-2-Pyridylmethyl)Amine]. J. Chem. Crystallogr. 2015, 45 (10–12), 476–483. 10.1007/s10870-015-0617-8.

[ref115] Nakon R.; Rechani P. R.; Angelici R. J. Copper(II) Complex Catalysis of Amino Acid Ester Hydrolysis. Correlation with Complex Stability. J. Am. Chem. Soc. 1974, 96 (7), 2117–2120. 10.1021/ja00814a021. 4833643

[ref116] Soibinet M.; Déchamps-Olivier I.; Mohamadou A.; Aplincourt M. X-Ray Crystal Structure, ESR and Potentiometric Studies of Copper(II) Complexes with (2-Pyridylmethyl, 3-Pyridylmethyl) Amine Ligand. Inorg. Chem. Commun. 2004, 7 (3), 405–409. 10.1016/j.inoche.2003.12.026.

[ref117] Roux A.; Talon R.; Alsalman Z.; Engilberge S.; D’Aléo A.; Di Pietro S.; Robin A.; Bartocci A.; Pilet G.; Dumont E.; Wagner T.; Shima S.; Riobé F.; Girard E.; Maury O. Influence of Divalent Cations in the Protein Crystallization Process Assisted by Lanthanide-Based Additives. Inorg. Chem. 2021, 60 (20), 15208–15214. 10.1021/acs.inorgchem.1c01635. 34597021

[ref118] Smits N. W. G.; Rademaker D.; Konovalov A. I.; Siegler M. A.; Hetterscheid D. G. H. Influence of the Spatial Distribution of Copper Sites on the Selectivity of the Oxygen Reduction Reaction. Dalton Transactions 2022, 51 (3), 1206–1215. 10.1039/D1DT03296H. 34951437 PMC8763313

[ref119] Tabbì G.; Giuffrida A.; Bonomo R. P. Determination of Formal Redox Potentials in Aqueous Solution of Copper(II) Complexes with Ligands Having Nitrogen and Oxygen Donor Atoms and Comparison with Their EPR and UV-Vis Spectral Features. J. Inorg. Biochem 2013, 128, 137–145. 10.1016/j.jinorgbio.2013.07.035. 23988848

[ref120] Horn A.; Fernandes C.; Bortoluzzi A. J.; Vugman N. V.; Herbst M. H. Coordination Chemistry of the New Ligand 1-(Bis-Pyridin-2-Ylmethyl-Amino)-3-Chloropropan-2-Ol (HPClNOL) with Copper(II). X-Ray Crystal Structure, Spectroscopic and Electrochemical Properties of the Complex [Cu(HPClNOL)(CH3CN)](ClO4)2. J. Mol. Struct. 2005, 749 (1–3), 96–102. 10.1016/j.molstruc.2005.03.045.

[ref121] Grujicic D.; Pesic B. Electrodeposition of Copper: The Nucleation Mechanisms. Electrochim. Acta 2002, 47 (18), 2901–2912. 10.1016/S0013-4686(02)00161-5.

[ref122] Smith J. R.; Campbell S. A.; Walsh F. C. Cyclic Voltammetry at Metal Electrodes. Transactions of the IMF 1995, 73 (2), 72–78. 10.1080/00202967.1995.11871062.

[ref125] Jomova K.; Baros S.; Valko M. Redox Active Metal-Induced Oxidative Stress in Biological Systems. Transition Metal Chemistry 2012, 37 (2), 127–134. 10.1007/s11243-012-9583-6.

[ref126] Sun Q.; Li Y.; Shi L.; Hussain R.; Mehmood K.; Tang Z.; Zhang H. Heavy Metals Induced Mitochondrial Dysfunction in Animals: Molecular Mechanism of Toxicity. Toxicology 2022, 469, 153136 10.1016/j.tox.2022.153136. 35202761

[ref127] Wang L.; Yin Y.-L.; Liu X.-Z.; Shen P.; Zheng Y.-G.; Lan X.-R.; Lu C.-B.; Wang J.-Z. Current Understanding of Metal Ions in the Pathogenesis of Alzheimer’s Disease. Transl Neurodegener 2020, 9 (1), 10 10.1186/s40035-020-00189-z. 32266063 PMC7119290

[ref128] Ben-Shushan S.; Miller Y. Neuropeptides: Roles and Activities as Metal Chelators in Neurodegenerative Diseases. J. Phys. Chem. B 2021, 125 (11), 2796–2811. 10.1021/acs.jpcb.0c11151. 33570949 PMC8389909

[ref129] Fawzi S. F.; Menze E. T.; Tadros M. G. Deferiprone Ameliorates Memory Impairment in Scopolamine-Treated Rats: The Impact of Its Iron-Chelating Effect on β-Amyloid Disposition. Behavioural Brain Research 2020, 378, 112314 10.1016/j.bbr.2019.112314. 31644927

[ref130] Ates G.; Goldberg J.; Currais A.; Maher P. CMS121, a Fatty Acid Synthase Inhibitor, Protects against Excess Lipid Peroxidation and Inflammation and Alleviates Cognitive Loss in a Transgenic Mouse Model of Alzheimer’s Disease. Redox Biol. 2020, 36, 101648 10.1016/j.redox.2020.101648. 32863221 PMC7394765

[ref131] Zuily L.; Lahrach N.; Fassler R.; Genest O.; Faller P.; Sénèque O.; Denis Y.; Castanié-Cornet M.-P.; Genevaux P.; Jakob U.; Reichmann D.; Giudici-Orticoni M.-T.; Ilbert M. Copper Induces Protein Aggregation, a Toxic Process Compensated by Molecular Chaperones. mBio 2022, 13 (2), e0325121 10.1128/mbio.03251-21. 35289645 PMC9040851

[ref132] Kang Z.; Qiao N.; Liu G.; Chen H.; Tang Z.; Li Y. Copper-Induced Apoptosis and Autophagy through Oxidative Stress-Mediated Mitochondrial Dysfunction in Male Germ Cells. Toxicology in Vitro 2019, 61, 104639 10.1016/j.tiv.2019.104639. 31491480

[ref133] Papa S.; Martino P. L.; Capitanio G.; Gaballo A.; De Rasmo D.; Signorile A.; Petruzzella V. The Oxidative Phosphorylation System in Mammalian Mitochondria. Adv. Exp. Med. Biol. 2012, 942, 3–37. 10.1007/978-94-007-2869-1_1. 22399416

[ref134] Lyublinskaya O.; Antunes F. Measuring Intracellular Concentration of Hydrogen Peroxide with the Use of Genetically Encoded H2O2 Biosensor HyPer. Redox Biol. 2019, 24, 101200 10.1016/j.redox.2019.101200. 31030065 PMC6482347

[ref135] Psomas G. Copper(II) and Zinc(II) Coordination Compounds of Non-Steroidal Anti-Inflammatory Drugs: Structural Features and Antioxidant Activity. Coord. Chem. Rev. 2020, 412, 213259 10.1016/j.ccr.2020.213259.

[ref136] Coulibaly K.; Thauvin M.; Melenbacher A.; Testard C.; Trigoni E.; Vincent A.; Stillman M. J.; Vriz S.; Policar C.; Delsuc N. A Di-Copper Peptidyl Complex Mimics the Activity of Catalase, a Key Antioxidant Metalloenzyme. Inorg. Chem. 2021, 60 (13), 9309–9319. 10.1021/acs.inorgchem.0c03718. 34109781

[ref137] Menezes L. B.; Segat B. B.; Tolentino H.; Pires D. C.; Mattos L. M. de M.; Hottum H. M.; Pereira M. D.; Latini A.; Horn A. Jr; Fernandes C. ROS Scavenging of SOD/CAT Mimics Probed by EPR and Reduction of Lipid Peroxidation in S. Cerevisiae and Mouse Liver, under Severe Hydroxyl Radical Stress Condition. J. Inorg. Biochem 2023, 239, 112062 10.1016/j.jinorgbio.2022.112062. 36403436

[ref138] Adjimani J. P.; Asare P. Antioxidant and Free Radical Scavenging Activity of Iron Chelators. Toxicol Rep 2015, 2, 721–728. 10.1016/j.toxrep.2015.04.005. 28962407 PMC5598521

[ref139] Gulcin İ.; Alwasel S. H. Metal Ions, Metal Chelators and Metal Chelating Assay as Antioxidant Method. Processes 2022, 10 (1), 132 10.3390/pr10010132.

[ref140] Cherrak S. A.; Mokhtari-Soulimane N.; Berroukeche F.; Bensenane B.; Cherbonnel A.; Merzouk H.; Elhabiri M. In Vitro Antioxidant versus Metal Ion Chelating Properties of Flavonoids: A Structure-Activity Investigation. PLoS One 2016, 11 (10), e0165575 10.1371/journal.pone.0165575. 27788249 PMC5082868

[ref141] AL Zahrani N. A.; El-Shishtawy R. M.; Asiri A. M. Recent Developments of Gallic Acid Derivatives and Their Hybrids in Medicinal Chemistry: A Review. Eur. J. Med. Chem. 2020, 204, 112609 10.1016/j.ejmech.2020.112609. 32731188

[ref142] Segat B. B.; Menezes L. B.; Cervo R.; Cargnelutti R.; Tolentino H.; Latini A.; Horn A. Jr; Fernandes C. Scavenging of Reactive Species Probed by EPR and Ex-Vivo Nanomolar Reduction of Lipid Peroxidation of Manganese Complexes. J. Inorg. Biochem 2023, 239, 112060 10.1016/j.jinorgbio.2022.112060. 36402588

[ref143] Mekhail M. A.; Smith K. J.; Freire D. M.; Pota K.; Nguyen N.; Burnett M. E.; Green K. N. Increased Efficiency of a Functional SOD Mimic Achieved with Pyridine Modification on a Pyclen-Based Copper(II) Complex. Inorg. Chem. 2023, 62 (14), 5415–5425. 10.1021/acs.inorgchem.2c04327. 36995929 PMC10820499

[ref144] Weder J. E.; Dillon C. T.; Hambley T. W.; Kennedy B. J.; Lay P. A.; Biffin J. R.; Regtop H. L.; Davies N. M. Copper Complexes of Non-Steroidal Anti-Inflammatory Drugs: An Opportunity yet to Be Realized. Coord. Chem. Rev. 2002, 232 (1–2), 95–126. 10.1016/S0010-8545(02)00086-3.

[ref145] Weser U.; Sellinger K.-H.; Lengfelder E.; Werner W.; Strähle J. Structure of Cu2(Indomethacin)4 and the Reaction with Superoxide in Aprotic Systems. Biochimica et Biophysica Acta (BBA) - General Subjects 1980, 631 (2), 232–245. 10.1016/0304-4165(80)90298-6. 6250635

[ref146] Anacona J. R.; Gutierrez C.; Rodriguez-Barbarin C. Crystal Structure and Superoxide Dismutase Activity of [Cu(Ethylenediamine)2Cl][PF6]. Monatsh. Chem. 2004, 135 (7), 785–792. 10.1007/s00706-003-0123-0.

[ref147] Adam S. M.; Wijeratne G. B.; Rogler P. J.; Diaz D. E.; Quist D. A.; Liu J. J.; Karlin K. D. Synthetic Fe/Cu Complexes: Toward Understanding Heme-Copper Oxidase Structure and Function. Chem. Rev. 2018, 118 (22), 10840–11022. 10.1021/acs.chemrev.8b00074. 30372042 PMC6360144

[ref148] Terra W. S.; Ferreira S. S.; Costa R. O.; Mendes L. L.; Franco R. W. A.; Bortoluzzi A. J.; Resende J. A. L. C.; Fernandes C.; Horn A. Evaluating the Influence of the Diamine Unit (Ethylenediamine, Piperazine and Homopiperazine) on the Molecular Structure, Physical Chemical Properties and Superoxide Dismutase Activity of Copper Complexes. Inorg. Chim. Acta 2016, 450, 353–363. 10.1016/j.ica.2016.06.024.

[ref149] Autzen S.; Korth H.; Boese R.; Groot H. de; Sustmann R. Studies of Pyridinyl-Containing 14-Membered Macrocyclic Copper(II) Complexes. Eur. J. Inorg. Chem. 2003, 2003 (7), 1401–1410. 10.1002/ejic.200390182.

[ref150] Guerreiro J. F.; Gomes M. A. G. B.; Pagliari F.; Jansen J.; Marafioti M. G.; Nistico C.; Hanley R.; Costa R. O.; Ferreira S. S.; Mendes F.; Fernandes C.; Horn A.; Tirinato L.; Seco J. Iron and Copper Complexes with Antioxidant Activity as Inhibitors of the Metastatic Potential of Glioma Cells. RSC Adv. 2020, 10 (22), 12699–12710. 10.1039/D0RA00166J. 35492123 PMC9051468

[ref151] Zhou Y.-H.; Tao J.; Lv Q.-C.; Jia W.-G.; Yun R.-R.; Cheng Y. Effect of the Amide Groups on Superoxide Dismutation Catalyzed by Copper(II) Complexes of Adamantane. Inorg. Chim. Acta 2015, 426, 211–220. 10.1016/j.ica.2014.11.019.

[ref152] O’Connor M.; Kellett A.; McCann M.; Rosair G.; McNamara M.; Howe O.; Creaven B. S.; McClean S.; Foltyn-Arfa Kia A.; O’Shea D.; Devereux M. Copper(II) Complexes of Salicylic Acid Combining Superoxide Dismutase Mimetic Properties with DNA Binding and Cleaving Capabilities Display Promising Chemotherapeutic Potential with Fast Acting in Vitro Cytotoxicity against Cisplatin Sensitive and Resistant Cancer Cell Lines. J. Med. Chem. 2012, 55 (5), 1957–1968. 10.1021/jm201041d. 22313179

[ref153] Müller J.; Felix K.; Maichle C.; Lengfelder E.; Strähle J.; Weser U. Phenyl-Substituted Copper Di-Schiff Base, a Potent Cu2Zn2 Superoxide Dismutase Mimic Surviving Competitive Biochelation. Inorg. Chim. Acta 1995, 233 (1–2), 11–19. 10.1016/0020-1693(94)04298-A.

[ref154] Luo Q.; Lu Q.; Dai A.; Huang L. A Study on the Structure and Properties of a New Model Compound of Cu(II)—Zn(II)-Superoxide Dismutase. J. Inorg. Biochem 1993, 51 (3), 655–662. 10.1016/0162-0134(93)85037-9.

[ref155] Weser U.; Schubotz L. M.; Lengfelder E. Imidazole-Bridged Copper Complexes as Cu2Zn2—Superoxide Dismutase Models. J. Mol. Catal. 1981, 13 (2), 249–261. 10.1016/0304-5102(81)85025-0.

[ref156] Suksrichavalit T.; Prachayasittikul S.; Piacham T.; Isarankura-Na-Ayudhya C.; Nantasenamat C.; Prachayasittikul V. Copper Complexes of Nicotinic-Aromatic Carboxylic Acids as Superoxide Dismutase Mimetics. Molecules 2008, 13 (12), 3040–3056. 10.3390/molecules13123040. 19078847 PMC6244828

[ref157] Quek S. Y.; Debnath S.; Laxmi S.; van Gastel M.; Krämer T.; England J. Sterically Stabilized End-On Superoxocopper(II) Complexes and Mechanistic Insights into Their Reactivity with O-H, N-H, and C-H Substrates. J. Am. Chem. Soc. 2021, 143 (47), 19731–19747. 10.1021/jacs.1c07837. 34783549

[ref158] Fujisawa K.; Tanaka M.; Moro-oka Y.; Kitajima N. A Monomeric Side-On Superoxocopper(II) Complex: Cu(O2)(HB(3-TBu-5-IPrpz)3). J. Am. Chem. Soc. 1994, 116 (26), 12079–12080. 10.1021/ja00105a069.

[ref159] Elwell C. E.; Gagnon N. L.; Neisen B. D.; Dhar D.; Spaeth A. D.; Yee G. M.; Tolman W. B. Copper-Oxygen Complexes Revisited: Structures, Spectroscopy, and Reactivity. Chem. Rev. 2017, 117 (3), 2059–2107. 10.1021/acs.chemrev.6b00636. 28103018 PMC5963733

[ref160] Valentine J. S.; Tatsuno Y.; Nappa M. Superoxotetraphenylporphinatozinc(1-). J. Am. Chem. Soc. 1977, 99 (10), 3522–3523. 10.1021/ja00452a066.

[ref161] Losada J.; del Peso I.; Beyer L. Electrochemical and Spectroelectrochemical Properties of Copper(II) Schiff-Base Complexes. Inorg. Chim. Acta 2001, 321 (1–2), 107–115. 10.1016/S0020-1693(01)00511-4.

[ref162] Faggi E.; Gavara R.; Bolte M.; Fajarí L.; Juliá L.; Rodríguez L.; Alfonso I. Copper(II) Complexes of Macrocyclic and Open-Chain Pseudopeptidic Ligands: Synthesis, Characterization and Interaction with Dicarboxylates. Dalton Trans. 2015, 44 (28), 12700–12710. 10.1039/C5DT01496D. 26085383

[ref163] Fielden E. M.; Roberts P. B.; Bray R. C.; Lowe D. J.; Mautner G. N.; Rotilio G.; Calabrese L. The Mechanism of Action of Superoxide Dismutase from Pulse Radiolysis and Electron Paramagnetic Resonance. Evidence That Only Half the Active Sites Function in Catalysis. Biochem. J. 1974, 139 (1), 49–60. 10.1042/bj1390049. 4377100 PMC1166250

[ref164] deAlvare L. R.; Goda K.; Kimura T. Mechanism of Superoxide Anion Scavenging Reaction by Bis-(Salicylato)-Copper(II) Complex. Biochem. Biophys. Res. Commun. 1976, 69 (3), 687–694. 10.1016/0006-291X(76)90930-X. 178316

[ref165] O'Young C.-L.; Lippard S. J. Reactions of Superoxide Anion with Copper(II) Salicylate Complexes. J. Am. Chem. Soc. 1980, 102, 4920–4924. 10.1021/ja00535a015.

[ref166] Zhao R.; Zhang B.-B.; Liu Z.; Cheng G.-J.; Wang Z.-X. DFT Mechanistic Insights into Aldehyde Deformylations with Biomimetic Metal-Dioxygen Complexes: Distinct Mechanisms and Reaction Rules. JACS Au 2022, 2 (3), 745–761. 10.1021/jacsau.2c00014. 35373207 PMC8970012

[ref167] Czaikowski M. E.; McNeece A. J.; Boyn J.-N.; Jesse K. A.; Anferov S. W.; Filatov A. S.; Mazziotti D. A.; Anderson J. S. Generation and Aerobic Oxidative Catalysis of a Cu(II) Superoxo Complex Supported by a Redox-Active Ligand. J. Am. Chem. Soc. 2022, 144 (34), 15569–15580. 10.1021/jacs.2c04630. 35977083 PMC10017013

[ref168] Würtele C.; Gaoutchenova E.; Harms K.; Holthausen M. C.; Sundermeyer J.; Schindler S. Crystallographic Characterization of a Synthetic 1:1 End-On Copper Dioxygen Adduct Complex. Angew. Chem., Int. Ed. 2006, 45 (23), 3867–3869. 10.1002/anie.200600351. 16671142

[ref169] Que L.; Tolman W. B. Biologically Inspired Oxidation Catalysis. Nature 2008, 455 (7211), 333–340. 10.1038/nature07371. 18800132

[ref170] Donoghue P. J.; Gupta A. K.; Boyce D. W.; Cramer C. J.; Tolman W. B. An Anionic, Tetragonal Copper(II) Superoxide Complex. J. Am. Chem. Soc. 2010, 132 (45), 15869–15871. 10.1021/ja106244k. 20977226 PMC3013377

[ref171] Bailey W. D.; Gagnon N. L.; Elwell C. E.; Cramblitt A. C.; Bouchey C. J.; Tolman W. B. Revisiting the Synthesis and Nucleophilic Reactivity of an Anionic Copper Superoxide Complex. Inorg. Chem. 2019, 58 (8), 4706–4711. 10.1021/acs.inorgchem.9b00090. 30901201 PMC6548509

[ref172] Baglia R. A.; Zaragoza J. P. T.; Goldberg D. P. Biomimetic Reactivity of Oxygen-Derived Manganese and Iron Porphyrinoid Complexes. Chem. Rev. 2017, 117 (21), 13320–13352. 10.1021/acs.chemrev.7b00180. 28991451 PMC6058703

[ref173] Jasniewski A. J.; Que L. Dioxygen Activation by Nonheme Diiron Enzymes: Diverse Dioxygen Adducts, High-Valent Intermediates, and Related Model Complexes. Chem. Rev. 2018, 118 (5), 2554–2592. 10.1021/acs.chemrev.7b00457. 29400961 PMC5920527

[ref174] Huang X.; Groves J. T. Oxygen Activation and Radical Transformations in Heme Proteins and Metalloporphyrins. Chem. Rev. 2018, 118 (5), 2491–2553. 10.1021/acs.chemrev.7b00373. 29286645 PMC5855008

[ref175] Jeong D.; Selverstone Valentine J.; Cho J. Bio-Inspired Mononuclear Nonheme Metal Peroxo Complexes: Synthesis, Structures and Mechanistic Studies toward Understanding Enzymatic Reactions. Coord. Chem. Rev. 2023, 480, 215021 10.1016/j.ccr.2023.215021.

[ref176] Davydov R.; Herzog A. E.; Jodts R. J.; Karlin K. D.; Hoffman B. M. End-On Copper(I) Superoxo and Cu(II) Peroxo and Hydroperoxo Complexes Generated by Cryoreduction/Annealing and Characterized by EPR/ENDOR Spectroscopy. J. Am. Chem. Soc. 2022, 144 (1), 377–389. 10.1021/jacs.1c10252. 34981938 PMC8785356

[ref177] Soibinet M.; Déchamps-Olivier I.; Mohamadou A.; Aplincourt M. X-Ray Crystal Structure, ESR and Potentiometric Studies of Copper(II) Complexes with (2-Pyridylmethyl, 3-Pyridylmethyl) Amine Ligand. Inorg. Chem. Commun. 2004, 7 (3), 405–409. 10.1016/j.inoche.2003.12.026.

[ref178] Liebhäuser P.; Keisers K.; Hoffmann A.; Schnappinger T.; Sommer I.; Thoma A.; Wilfer C.; Schoch R.; Stührenberg K.; Bauer M.; Dürr M.; Ivanović-Burmazović I.; Herres-Pawlis S. Record Broken: A Copper Peroxide Complex with Enhanced Stability and Faster Hydroxylation Catalysis. Chem. - Eur. J. 2017, 23 (50), 12171–12183. 10.1002/chem.201700887. 28425134

[ref179] Henson M. J.; Mukherjee P.; Root D. E.; Stack T. D. P.; Solomon E. I. Spectroscopic and Electronic Structural Studies of the Cu(III) _2_ Bis-μ-Oxo Core and Its Relation to the Side-On Peroxo-Bridged Dimer. J. Am. Chem. Soc. 1999, 121 (44), 10332–10345. 10.1021/ja9918425.

[ref180] Jacobson R. R.; Tyeklar Z.; Farooq A.; Karlin K. D.; Liu S.; Zubieta J. A Copper-Oxygen (Cu2-O2) Complex. Crystal Structure and Characterization of a Reversible Dioxygen Binding System. J. Am. Chem. Soc. 1988, 110 (11), 3690–3692. 10.1021/ja00219a071.

[ref181] Hoppe T.; Schaub S.; Becker J.; Würtele C.; Schindler S. Characterization of a Macrocyclic End-on Peroxido Copper Complex. Angew. Chem., Int. Ed. 2013, 52 (3), 870–873. 10.1002/anie.201205663. 23180566

[ref182] Vargo N. P.; Harland J. B.; Musselman B. W.; Lehnert N.; Ertem M. Z.; Robinson J. R. Calcium-Ion Binding Mediates the Reversible Interconversion of *Cis* and *Trans* Peroxido Dicopper Cores. Angew. Chem., Int. Ed. 2021, 60 (36), 19836–19842. 10.1002/anie.202105421. 34101958

[ref183] Brinkmeier A.; Dalle K. E.; D’Amore L.; Schulz R. A.; Dechert S.; Demeshko S.; Swart M.; Meyer F. Modulation of a μ-1,2-Peroxo Dicopper(II) Intermediate by Strong Interaction with Alkali Metal Ions. J. Am. Chem. Soc. 2021, 143 (42), 17751–17760. 10.1021/jacs.1c08645. 34658244

[ref184] Birben E.; Sahiner U. M.; Sackesen C.; Erzurum S.; Kalayci O. Oxidative Stress and Antioxidant Defense. World Allergy Organization Journal 2012, 5 (1), 9–19. 10.1097/WOX.0b013e3182439613. 23268465 PMC3488923

[ref185] Krystynik P. Advanced Oxidation Processes (AOPs) - Utilization of Hydroxyl Radical and Singlet Oxygen. Reactive Oxygen Species; InTech, 2022. 10.5772/intechopen.98189

[ref186] Akagawa M. Protein Carbonylation: Molecular Mechanisms, Biological Implications, and Analytical Approaches. Free Radic Res. 2021, 55 (4), 307–320. 10.1080/10715762.2020.1851027. 33183115

[ref187] Foret M. K.; Lincoln R.; Do Carmo S.; Cuello A. C.; Cosa G. Connecting the “Dots”: From Free Radical Lipid Autoxidation to Cell Pathology and Disease. Chem. Rev. 2020, 120 (23), 12757–12787. 10.1021/acs.chemrev.0c00761. 33211489

[ref188] Dizdaroglu M.; Jaruga P. Mechanisms of Free Radical-Induced Damage to DNA. Free Radic Res. 2012, 46 (4), 382–419. 10.3109/10715762.2011.653969. 22276778

[ref189] Nagarajan S.; Skillen N. C.; Fina F.; Zhang G.; Randorn C.; Lawton L. A.; Irvine J. T. S.; Robertson P. K. J. Comparative Assessment of Visible Light and UV Active Photocatalysts by Hydroxyl Radical Quantification. J. Photochem. Photobiol. A Chem. 2017, 334, 13–19. 10.1016/j.jphotochem.2016.10.034.

[ref190] Pham A. N.; Xing G.; Miller C. J.; Waite T. D. Fenton-like Copper Redox Chemistry Revisited: Hydrogen Peroxide and Superoxide Mediation of Copper-Catalyzed Oxidant Production. J. Catal. 2013, 301, 54–64. 10.1016/j.jcat.2013.01.025.

[ref191] Lipinski B. Hydroxyl Radical and Its Scavengers in Health and Disease. Oxid Med. Cell Longev 2011, 2011, 1–9. 10.1155/2011/809696. PMC3166784 21904647

[ref192] Kehrer J. P. The Haber-Weiss Reaction and Mechanisms of Toxicity. Toxicology 2000, 149 (1), 43–50. 10.1016/S0300-483X(00)00231-6. 10963860

[ref193] Prakash D.; Suri S.; Upadhyay G.; Singh B. N. Total Phenol, Antioxidant and Free Radical Scavenging Activities of Some Medicinal Plants. Int. J. Food Sci. Nutr 2007, 58 (1), 18–28. 10.1080/09637480601093269. 17415953

[ref194] Özyürek M.; Bektaşoǧlu B.; Güçlü K.; Apak R. Hydroxyl Radical Scavenging Assay of Phenolics and Flavonoids with a Modified Cupric Reducing Antioxidant Capacity (CUPRAC) Method Using Catalase for Hydrogen Peroxide Degradation. Anal. Chim. Acta 2008, 616 (2), 196–206. 10.1016/j.aca.2008.04.033. 18482604

[ref195] Cheng Z.; Ren J.; Li Y.; Chang W.; Chen Z. Study on the Multiple Mechanisms Underlying the Reaction between Hydroxyl Radical and Phenolic Compounds by Qualitative Structure and Activity Relationship. Bioorg. Med. Chem. 2002, 10 (12), 4067–4073. 10.1016/S0968-0896(02)00267-5. 12413860

[ref196] Moore J.; Yin J.-J.; Yu L. Novel Fluorometric Assay for Hydroxyl Radical Scavenging Capacity (HOSC) Estimation. J. Agric. Food Chem. 2006, 54 (3), 617–626. 10.1021/jf052555p. 16448158

[ref197] Hou W.-C.; Lin R.-D.; Cheng K.-T.; Hung Y.-T.; Cho C.-H.; Chen C.-H.; Hwang S.-Y.; Lee M.-H. Free Radical-Scavenging Activity of Taiwanese Native Plants. Phytomedicine 2003, 10 (2–3), 170–175. 10.1078/094471103321659898. 12725572

