# Supplementary material for: A Multipurpose Metallophore and Its Copper Complexes with Diverse Catalytic Antioxidant Properties to Deal with Metal and Oxidative Stress Disorders: A Combined Experimental, Theoretical, and In Vitro Study
Source: Inorg Chem. 2024 Jul 30;63(32):14827–50. doi: 10.1021/acs.inorgchem.4c00232 (PMC11323273; doi:10.1021/acs.inorgchem.4c00232)
Supplement: Supplementary file 1 — ic4c00232_si_001.pdf [file ic4c00232_si_001.pdf]

Supporting information

A multipurpose metallophore and its copper complexes with diverse catalytic antioxidant properties to deal with metal and oxidative stress disorders: A combined experimental, theoretical and *in vitro* study

Lucas B. Menezes,[a] Raquel M. S. N. Sampaio,[b] Lino Meurer,[a] Bruno Szpoganicz,[a] Rodrigo Cervo,[c] Roberta Cargnelutti,[c], Lukun Wang,[d] Jiawen Yang,[d] Rajeev Prabhakar,[d*] Christiane Fernandes, [a*] Adolfo Horn Jr.[a,*]

[a] Departamento de Química, Universidade Federal de Santa Catarina, 88040-900, Florianópolis, SC, Brazil.

[b] Laboratório de Ciências Químicas, Universidade Estadual do Norte Fluminense Darcy Ribeiro, 28013-602, Campos dos Goytacazes, RJ, Brazil

[c]Departamento de Química, Universidade Federal de Santa Maria, 97105-900, Santa Maria, RS, Brazil.

[d] Department of Chemistry, University of Miami, Coral Gables, FL 33146, USA.

*Corresponding authors:

Adolfo Horn Jr. (adolfo.junior@ufsc.br)

Christiane Fernandes (christiane.horn@ufsc.br)

Rajeev Prabhakar (rpr@miami.edu)

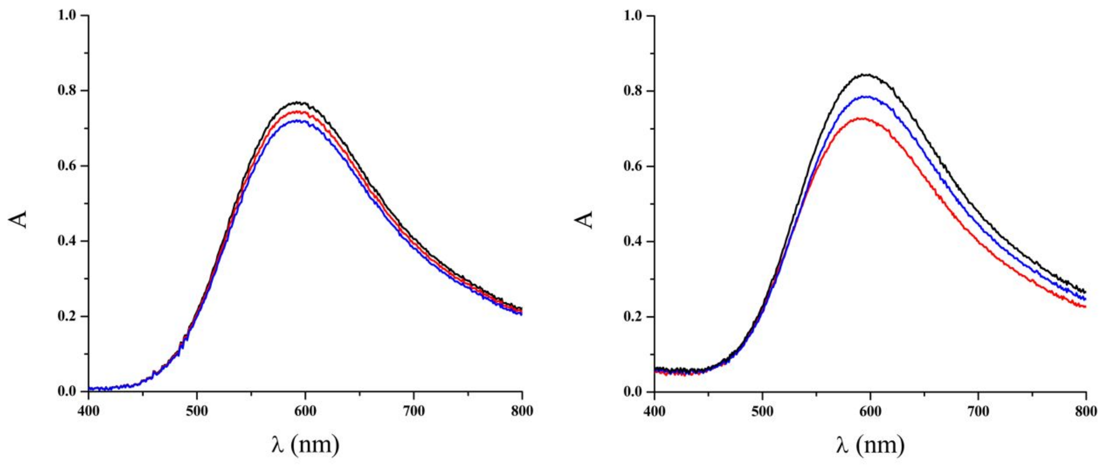

Figure S1. Evaluation of the stability of complexes **1** (left) and **2** (right) in PBS solution at 0 h (black line), 24 h (red line) and 48 h (blue line).

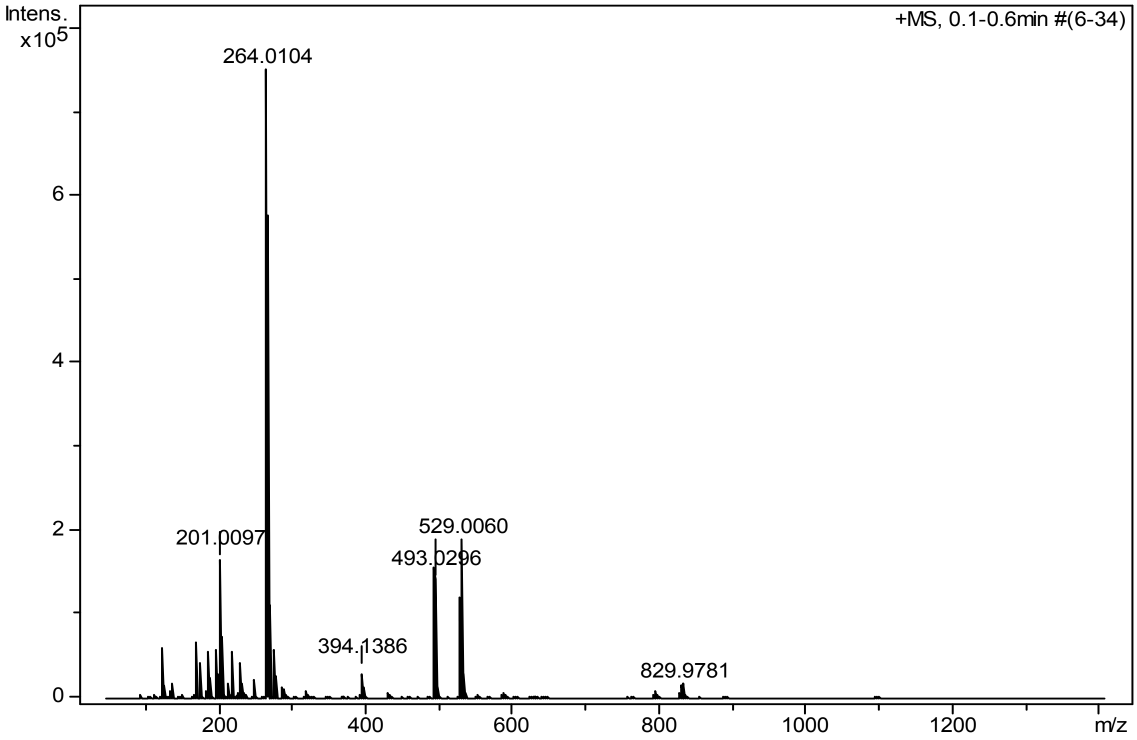

Figure S2. ESI-(+)-MS data of a MeOH:H_2O (1:1) solution of complex **1**.

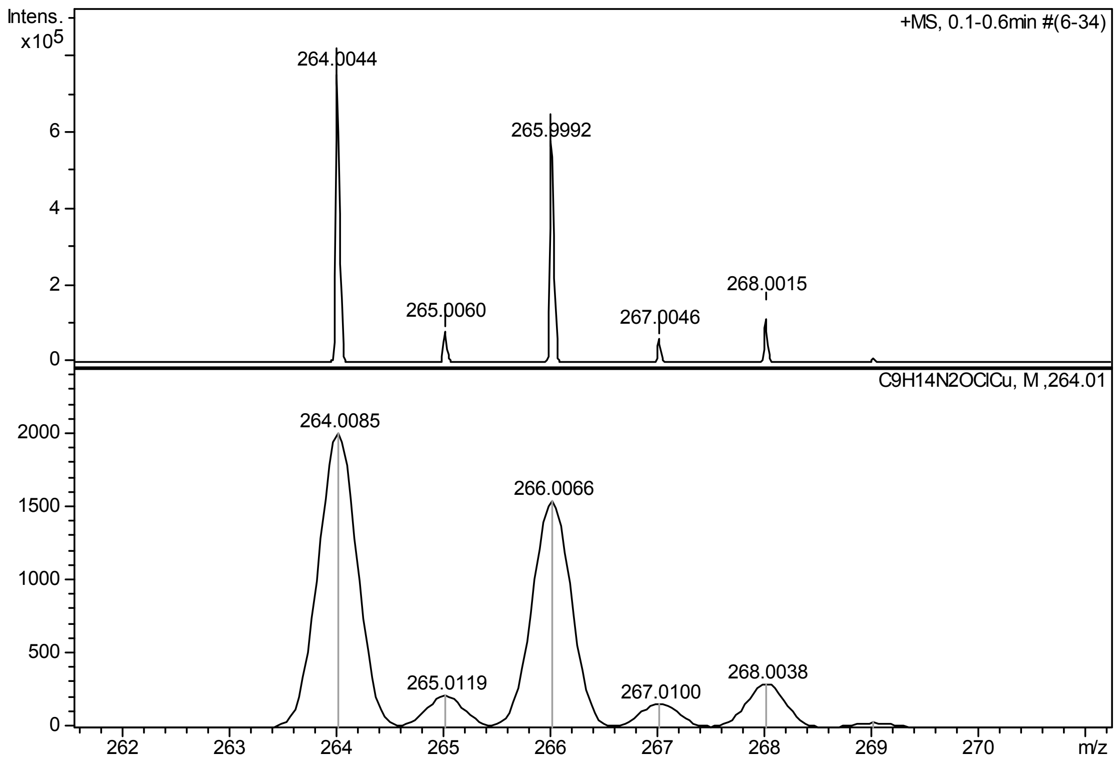

Figure S3. Experimental (top) and simulated (bottom) isotopic pattern for the ion at m/z 264.

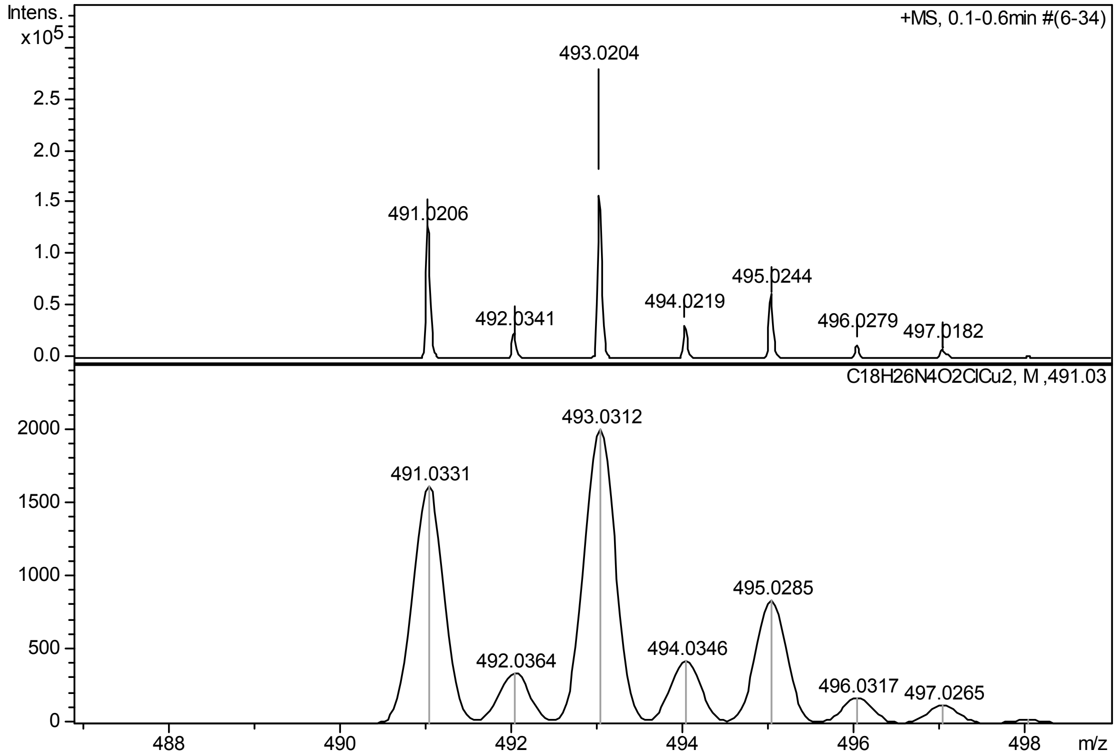

Figure S4. Experimental (top) and simulated (bottom) isotopic pattern for the ion at m/z 493.

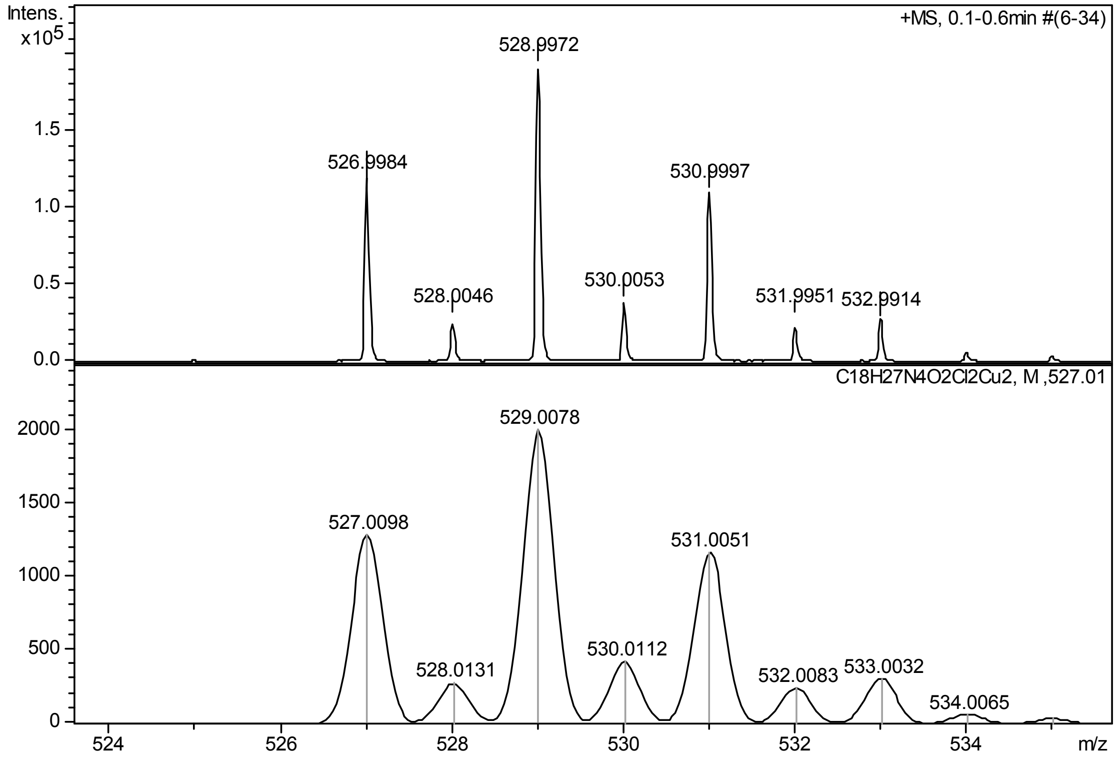

Figure S5. Experimental (top) and simulated (bottom) isotopic pattern for the ion at m/z 529.

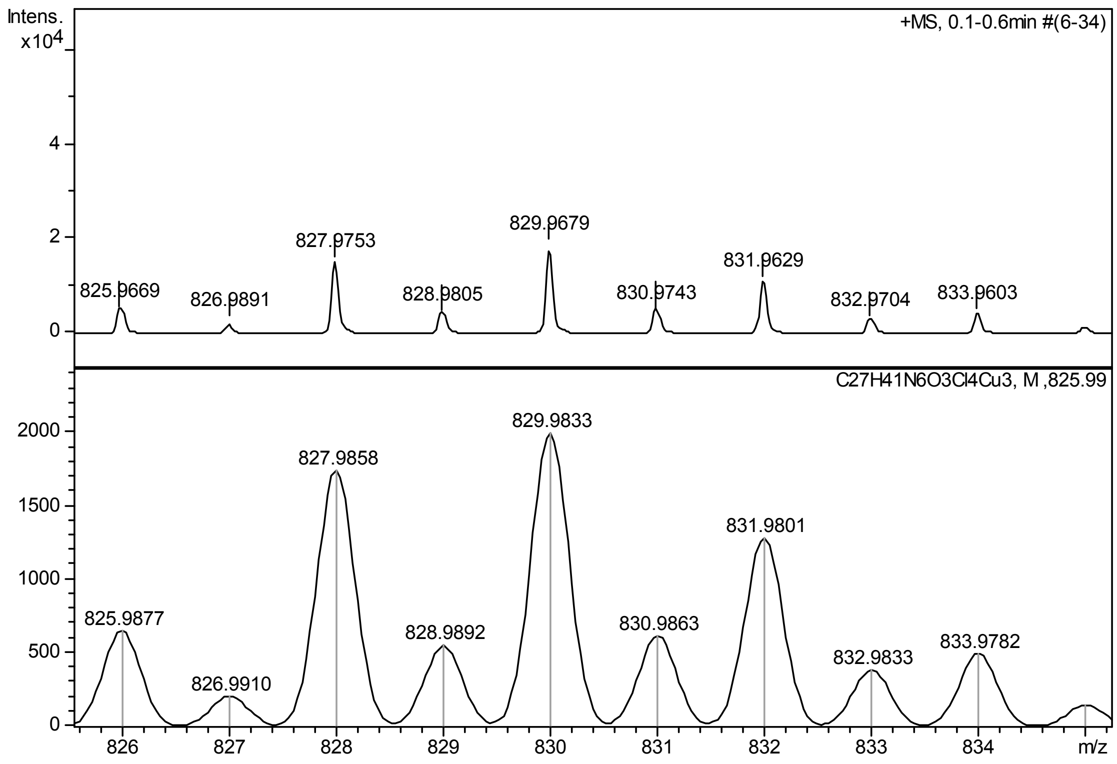

Figure S6. Experimental (top) and simulated (bottom) isotopic pattern for the ion at m/z 830.

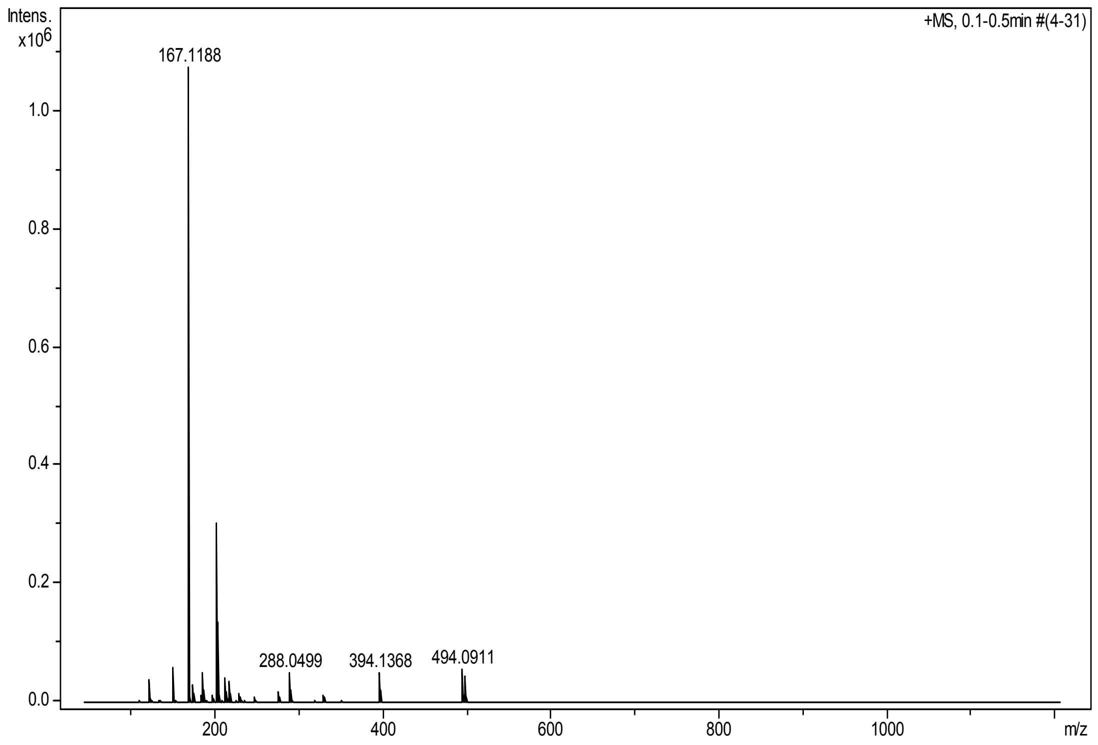

Figure S7. ESI-(+)-MS data of a MeOH:H_2O (1:1) solution of complex **2**.

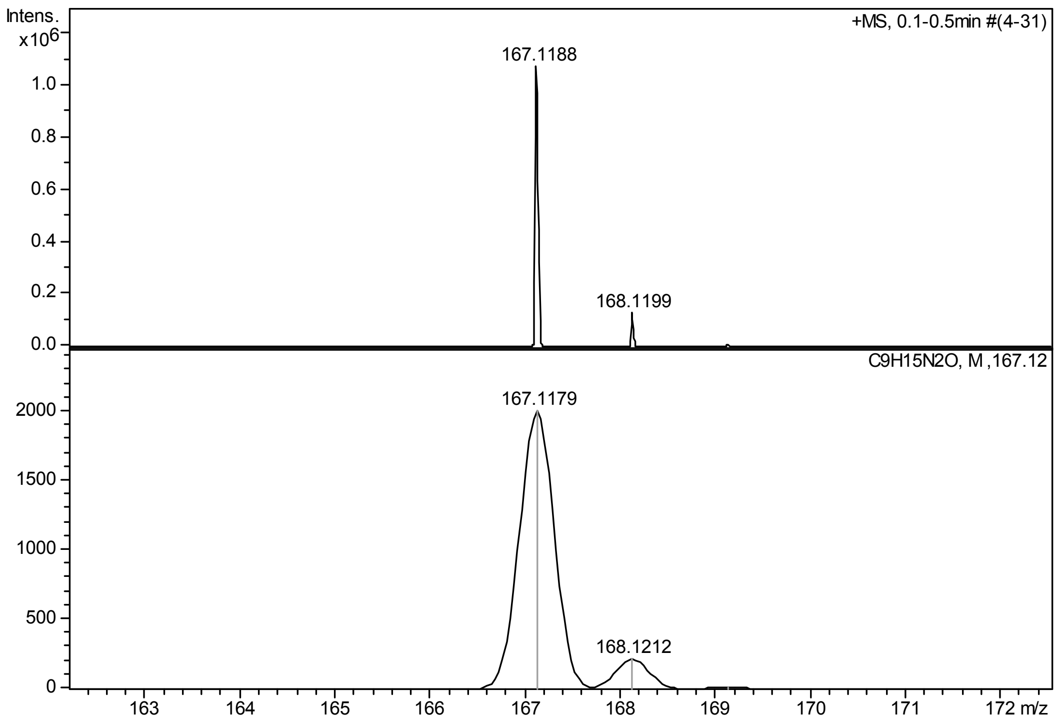

Figure S8. Experimental (top) and simulated (bottom) isotopic pattern for the ion at m/z 167.

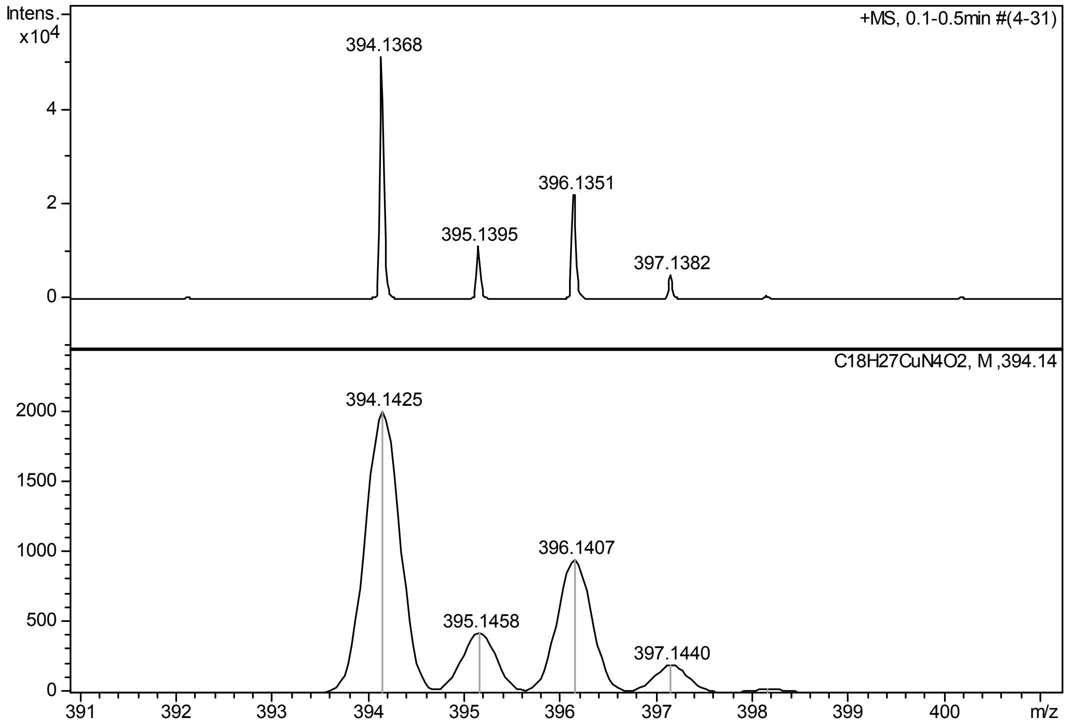

Figure S9. Experimental (top) and simulated (bottom) isotopic pattern for the ion at m/z 394.

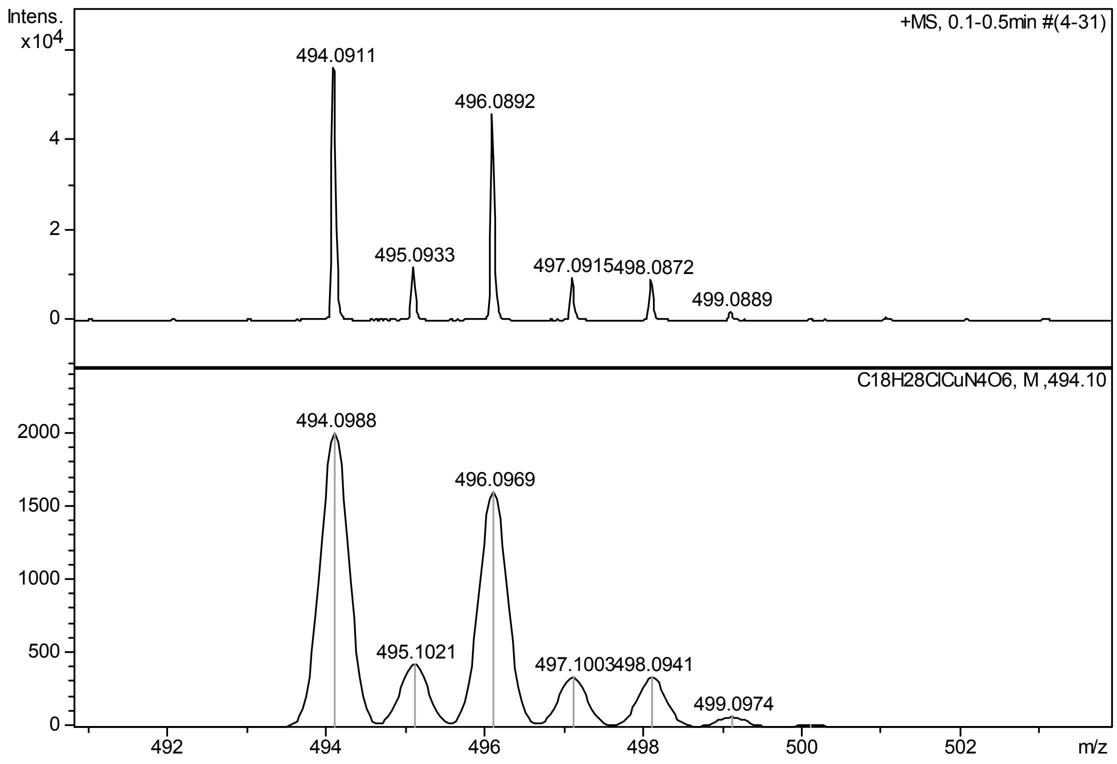

Figure S10. Experimental (top) and simulated (bottom) isotopic pattern for the ion at m/z 494.

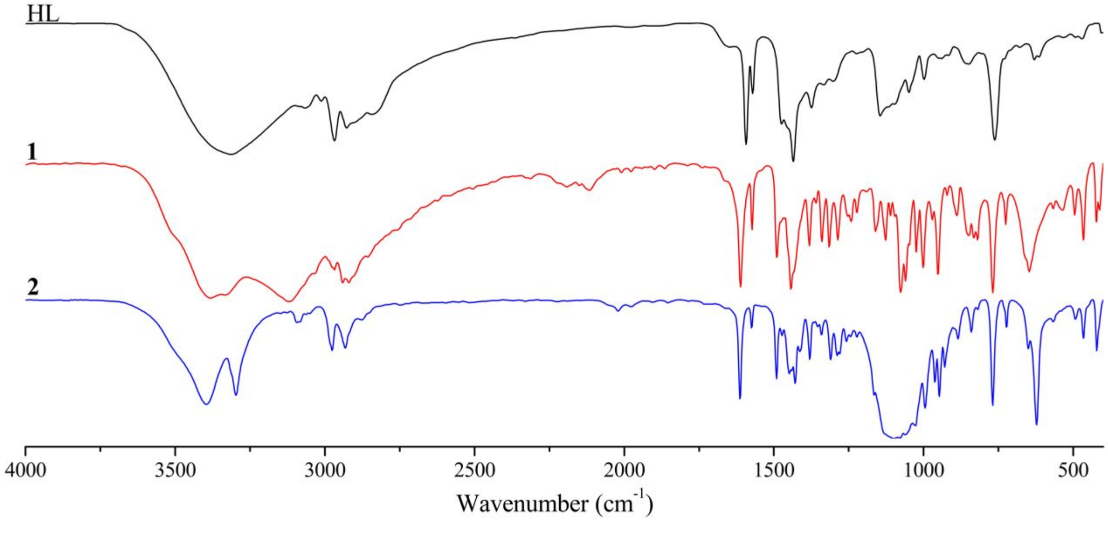

Figure S11. IR spectra of the ligand HL and its complexes **1** and **2**.

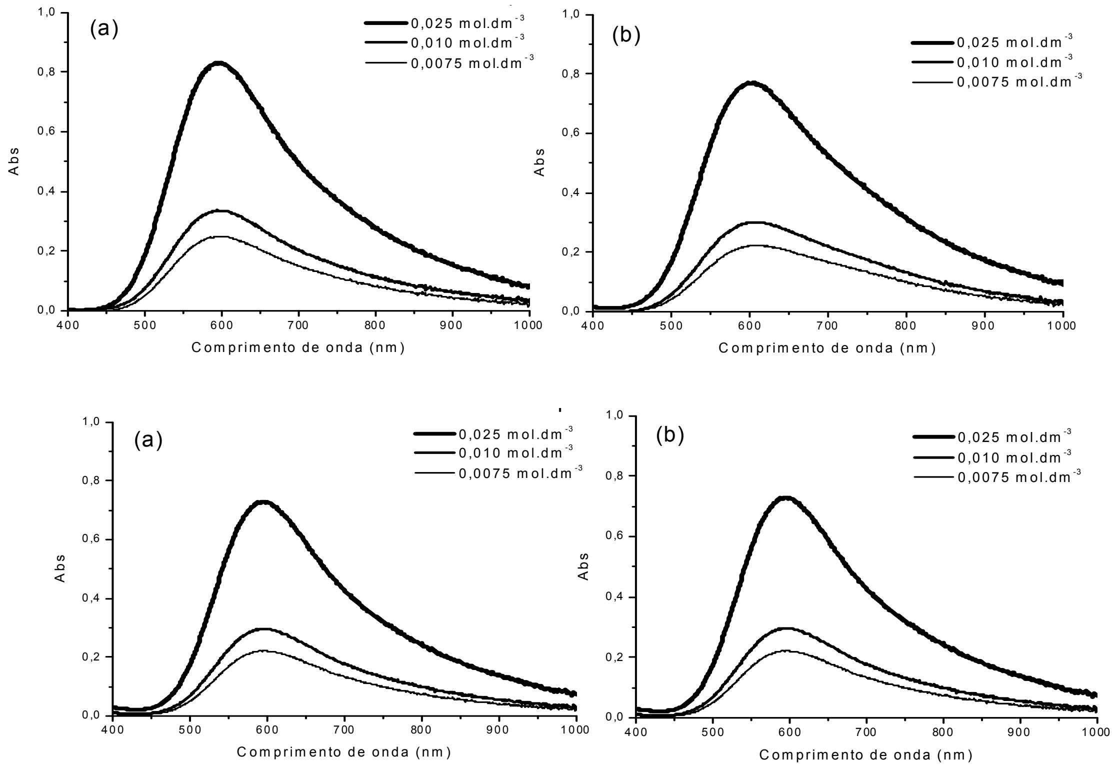

Figure S12. UV-Vis spectra of the compounds **1** (top) and **2** (bottom) at different concentrations in deionized water (a) and phosphate buffer (b) (0.1 mol L^{-1} , pH = 7.4).

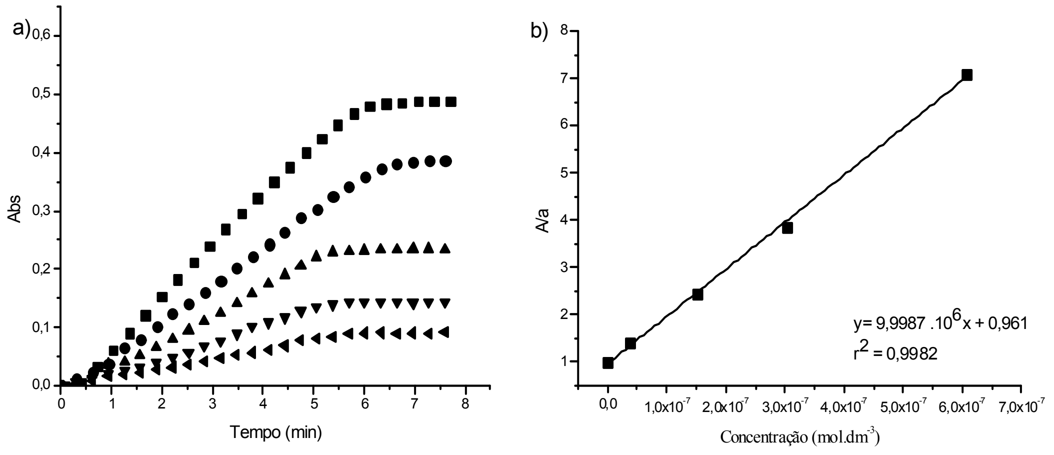

Figure S13. (a) Plot of the inhibition of NBT reduction by superoxide anion generated by xanthine/xanthine oxidase system measured at 560 nm at different concentrations of the compound **1** (■ no compound; ● $3.79x10^{-8}$; ▲ $1.51x10^{-7}$; ▼$3.03x10^{-7}$; ◄ $6.06x10^{-7}$ mol L^{-1}). (b) Plot of A/a *vs* complex concentration used for IC_{50} calculations, here "A" represents

the maximum absorption in the absence of the complex, while “a” represents the maximum absorption in the presence of the complex. The IC_{50} is computed at A/a = 2.

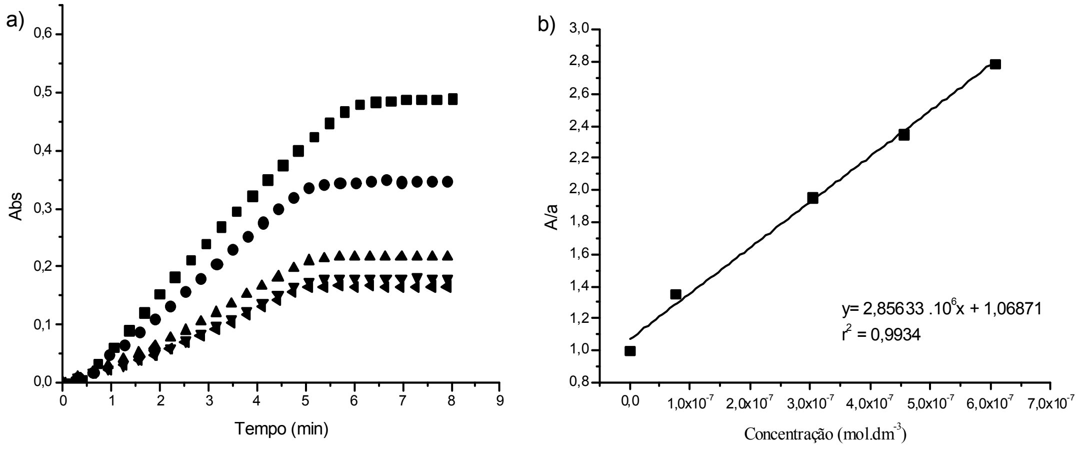

Figure S14. (a) Plot of the inhibition of NBT reduction by superoxide anion generated by xanthine/xanthine oxidase system measured at 560 nm at different concentrations of the compound **2** (■ no compound; ● $3.79x10^{-8}$; ▲ $1.51x10^{-7}$; ▼$3.03x10^{-7}$; ◄ $6.06x10^{-7}$ mol L^{-1}). (b) Plox’t of A/a *vs* complex concentration employed for IC_{50} calculation, here “A” represents the maximum absorption in the absence of the complex, while “a” represents the maximum absorption in the presence of the complex. The IC_{50} is computed at A/a = 2.

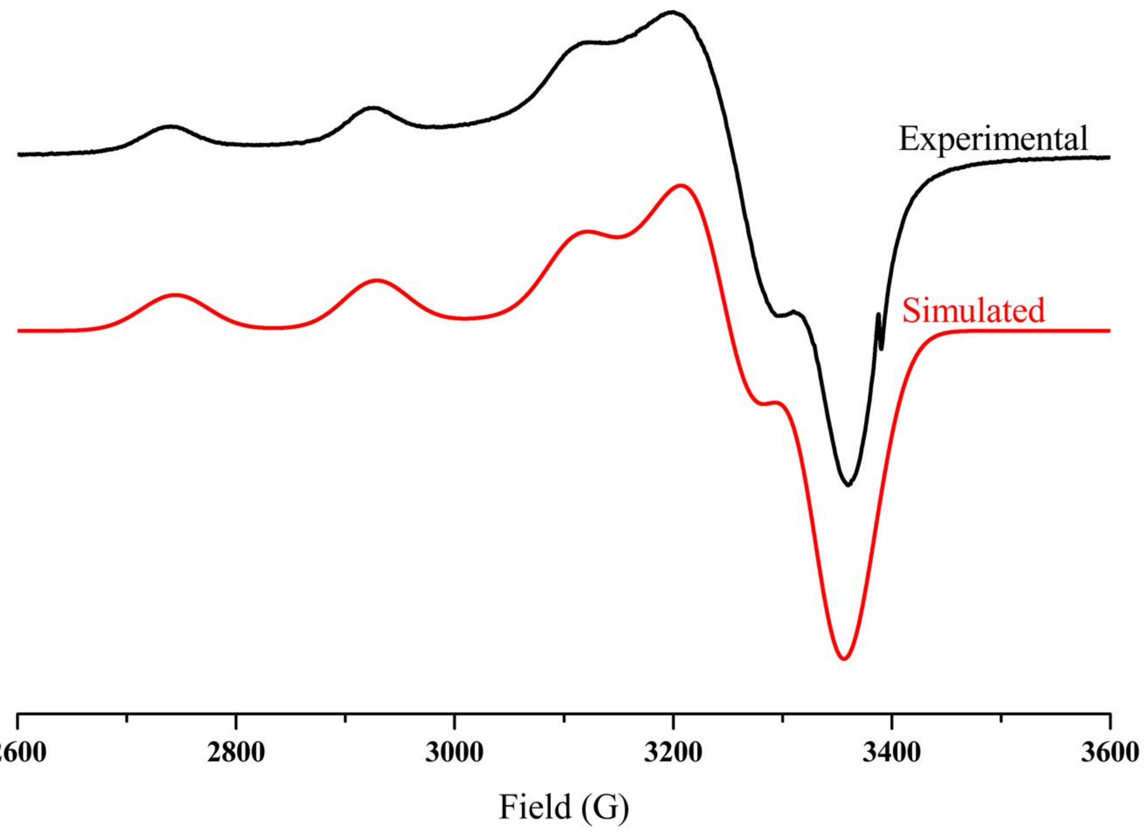

Figure S15. Experimental and simulated EPR spectra for complex **1** in PBS at 120 K. EPR parameters: gx = 2.067, gy= 2.098, and gz = 2.25 and Ax = 10.078 G, Ay = 8.86 G, and Az = 180.973 G .

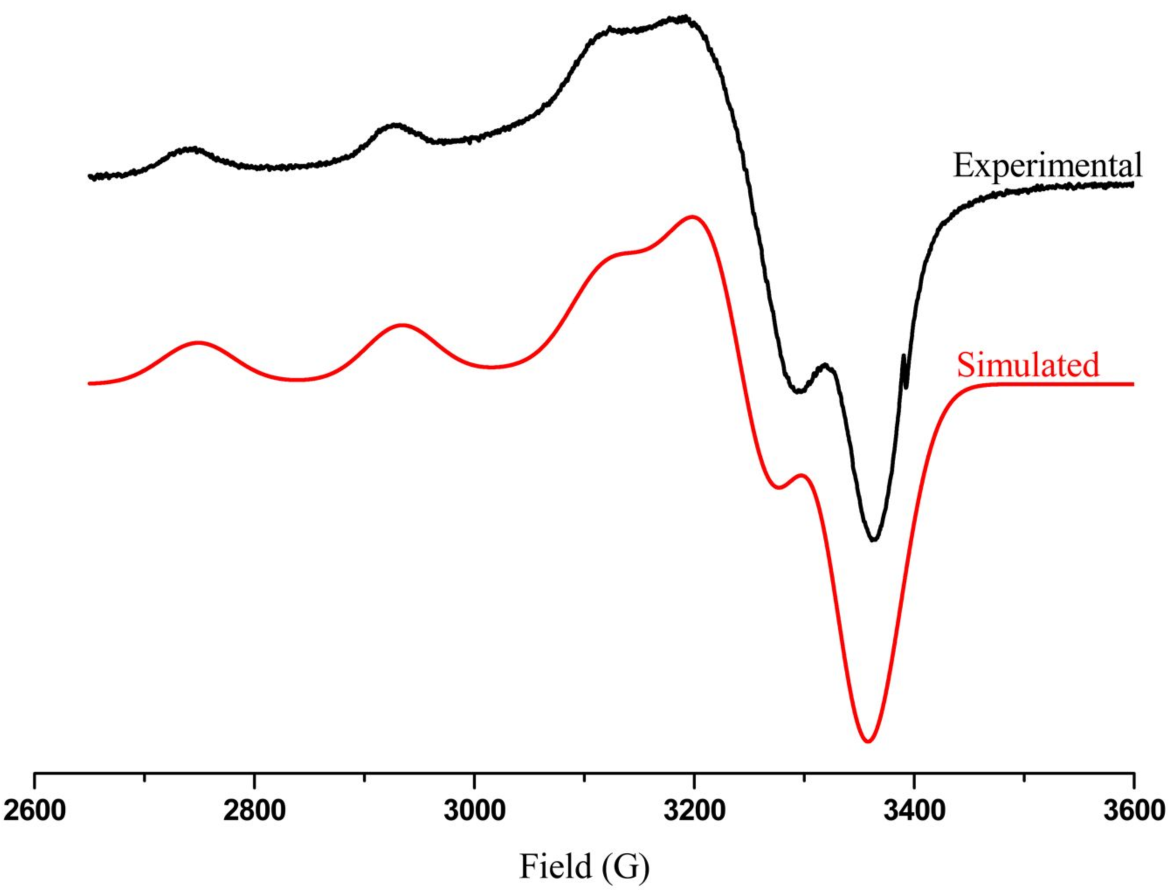

Figure S16. Experimental and simulated EPR spectra for complex **2** in PBS at 120 K. EPR parameters: gx = 2.102, gy= 2.069, and gz = 2.244; Ax = 0.867, Ay = 0.701, and Az = 170.

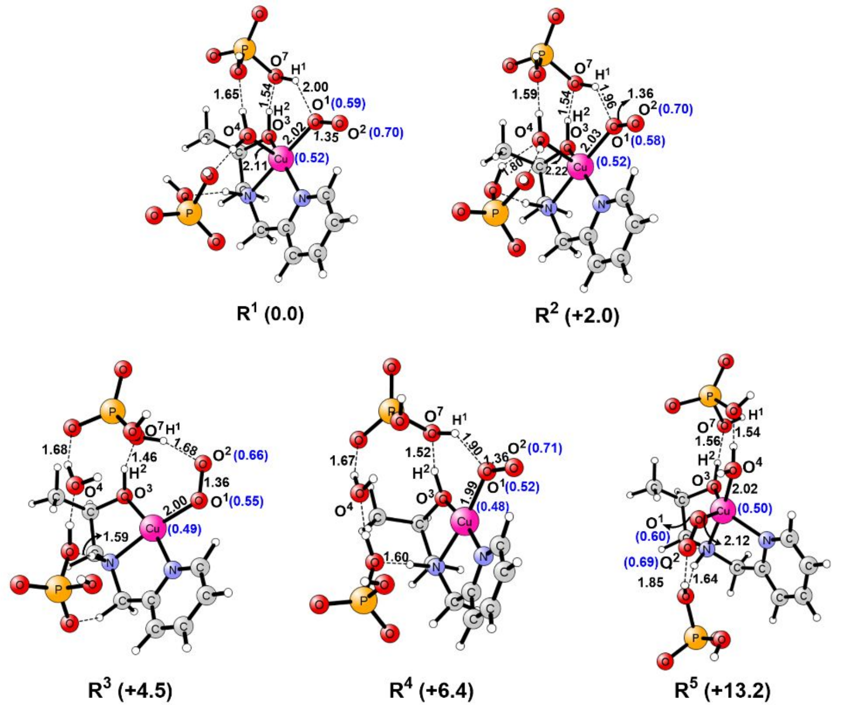

(a)

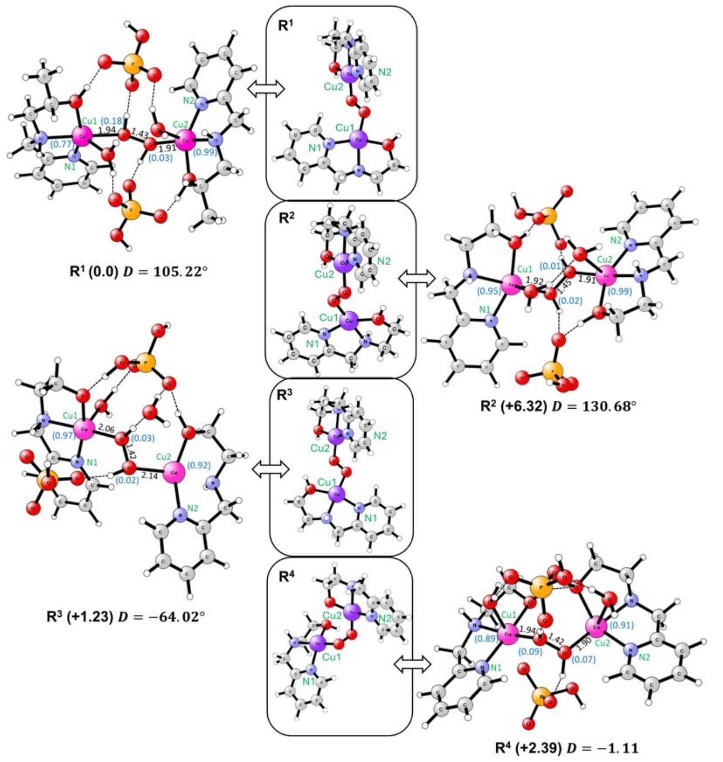

(b)

Figure S17. Different optimized conformations in the SOD **(a)** and CAT **(b)** activities of complex **1**.

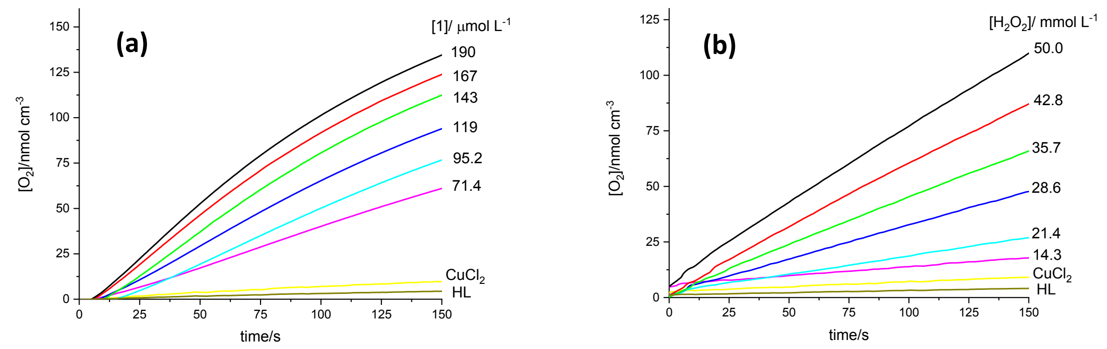

Figure S18. Plots of the catalase activity for compound **1** showing the dependence of the oxygen production with complex **1** (a) and H_2O_2 (b) concentration (as indicated). In panel (a) the concentration of H_2O_2 is constant (428 mmol L^{-1}) and in panel (b) the concentration of the complex is constant (119 µmol L^{-1}). Curves indicated as $CuCl_2$ and HL are related to the control reactions. The concentrations of the species in the control reactions ($CuCl_2$, HL) were equal to the highest complex **1** concentration (190 µmol L^{-1}) in panel (a) and equal to the concentration of **1** in panel (b) (119 µmol L^{-1}). In this latter, the highest concentration of H_2O_2 was used (50 mmol L^{-1}) in the control reaction.

Table S1. Crystallographic and structure refinement data for compounds **1** and **2.**

Compound	**1**	**2**
Formula	$C_9H_{14}Cl_2CuN_2O$	$C_{18}H_{28}Cl_2CuN_4O_{10}$
F.W. (g·mol^{-1})	300.66	594.88
Crystal system	Triclinic	Triclinic
Space group	*P*-1	*P*-1
a (Å)	7.6702(12)	8.737(4)
b (Å)	8.1209(10)	8.779(4)
c (Å)	9.938(2)	8.786(3)
α (°)	102.596(10)	75.512(9)
β (°)	93.483(10)	72.020(8)
γ (°)	103.038(14)	84.374(12)
T (K)	100(2)	300(2)
V (Å^3)	584.64(17)	620.5(4)
Z	2	1
$r_{calc.}$ (g·cm^{-3})	1.708	1.592
μ (mm^{-1})	2.299	1.155
F(000)	306	307
Reflections collected	29113	41884
Independent reflections	3589 [R(int) = 0.0232]	3810 [R(int) = 0.0294]
R_1 $[I > 2\sigma(I)]$	0.0192	0.0551
wR_2 [I > 2σ(I)]	0.0465	0.1524
R_1 (all data)[a]	0.0217	0.0633
wR_2 (all data)[b]	0.0477	0.1605
GOOF on F^2	1.101	1.044
Largest diff. peak and hole (e.Å^{-3})	0.456 and -0.537	1.625 and -0.727

[a]$R_1 = \sum||F_o|-|F_c||/\sum|F_o|$; [b]$wR_2 = \{\sum w(F_o^2-F_c^2)^2/\sum w(F_o^2)^2\}^{½}$

Steady-state treatment was employed to determine the elementary steps involved in the oxygen production in the reaction between 1 and H_2O_2 (see Scheme 3 in the article)

Considering a steady state approach, the O_2 production can be given by:

$d[O_2]/dt = k_3[[(HL1)Cu^{2+}\text{-}(H_2O_2)\text{-}Cu^{2+}(HL1)]^{4+}]$ (1)

The $[(HL1)Cu^{2+}\text{-}(H_2O_2)\text{-}Cu^{2+}(HL1)]$ is dependent on its formation rate, which may be obtained by:

$d[[(HL1)Cu^{2+}\text{-}(H_2O_2)\text{-}Cu^{2+}(HL1)]^{4+}]/dt = k_2[[Cu^{2+}(HL1)(H_2O_2)]^{2+}][[Cu^{2+}(HL1)]^{2+}] - k_3[[(HL1)Cu^{2+}\text{-}(H_2O_2)\text{-}Cu^{2+}(HL1)]^{4+}] = 0$

$k_2[[Cu^{2+}(HL1)(H_2O_2)]^{2+}][[Cu^{2+}(HL1)]^{2+}] = k_3[[(HL1)Cu^{2+}\text{-}(H_2O_2)\text{-}Cu^{2+}(HL1)]^{4+}]$

$[[(HL1)Cu^{2+}\text{-}(H_2O_2)\text{-}Cu^{2+}(HL1)]^{4+}] = k_2/k_3 [[Cu^{2+}(HL1)(H_2O_2)]^{2+}][[Cu^{2+}(HL1)]^{2+}]$ (2)

The $[Cu^{2+}(HL1)(H_2O_2)]^{2+}$ species is dependent on its rate formation, which may be obtained by:

$d[[Cu^{2+}(HL1)(H_2O_2)]^{2+}]/dt= k_1[[Cu(HL1)]^{2+}][H_2O_2] - k_2[[Cu^{2+}(HL1)(H_2O_2)]^{+}][Cu(HL1)]^{2+}] = 0$

$k_1[[Cu(HL1)]^{2+}] [H_2O_2] = k_2[[Cu^{2+}(HL1)(H_2O_2)]][[Cu(HL1)]^{2+}]$

$[[Cu^{2+}(HL1)(H_2O_2)]] = (k_1/k_2)[H_2O_2]$ (3)

Thus, replacing equation (3) in (2):

$[[(HL1)Cu^{2+}\text{-}(H_2O_2)\text{-}Cu^{2+}(HL1)]] = (k_2/k_3) [[Cu^{2+}(HL1)(H_2O_2)]][[Cu^{2+}(HL1)]^{2+}]$ (2)

$[[Cu^{2+}(HL1)(H_2O_2)]] = (k_1/k_2)[H_2O_2]$ (3)

$[[(HL1)Cu^{2+}\text{-}(H_2O_2)\text{-}Cu^{2+}(HL1)]^{4+}] = (k_2/k_3)(k_1/k_2)[H_2O_2][[Cu^{2+}(HL1)]^{2+}]$

$[[(HL1)Cu^{2+}\text{-}(H_2O_2)\text{-}Cu^{2+}(HL1)]^{4+}] = k_1/k_3[H_2O_2]\ [[Cu^{2+}(HL1)]^{2+}]$ (2a)

Replacing equation (2a) in (1):

$d[O_2]/dt = k_3[[(HL1)Cu^{2+}\text{-}(H_2O_2)\text{-}Cu^{2+}(HL1)]^{4+}]$ (1)

$[[(HL1)Cu^{2+}\text{-}(H_2O_2)\text{-}Cu^{2+}(HL1)]^{4+}] = k_1/k_3[H_2O_2]\ [[Cu^{2+}(HL1)]^{2+}]$ (2a)

$d[O_2]/dt = k_3k_1/k_3[H_2O_2]\ [[Cu^{2+}(HL1)]^{2+}]$

$\mathbf{d[O_2]/dt = k_1[H_2O_2][[Cu^{2+}(HL1)]^{2+}]}$
